# Magnetohydrodynamic double-diffusive peristaltic flow of radiating fourth-grade nanofluid through a porous medium with viscous dissipation and heat generation/absorption

**DOI:** 10.1038/s41598-023-39756-5

**Published:** 2023-08-11

**Authors:** R. A. Mohamed, S. M. Abo-Dahab, A. M. Abd-Alla, M. S. Soliman

**Affiliations:** 1 Mathematics Department, Faculty of Science, South Valley University, Qena, Egypt; 2 Mathematics Department, Faculty of Science, Sohag University, Sohag, Egypt

**Keywords:** Materials science, Physics

## Abstract

This article focuses on determining how to double diffusion affects the non-Newtonian fourth-grade nanofluids peristaltic motion within a symmetrical vertical elastic channel supported by a suitable porous medium as well as, concentrating on the impact of a few significant actual peculiarities on the development of the peristaltic liquid, such as rotation, initial pressure, non-linear thermal radiation, heat generation/absorption in the presence of viscous dissipation and joule heating with noting that the fluid inside the channel is subject to an externally induced magnetic field, giving it electromagnetic properties. Moreover, the constraints of the long-wavelength approximation and neglecting the wave number along with the low Reynolds number have been used to transform the nonlinear partial differential equations in two dimensions into a system of nonlinear ordinary differential equations in one dimension, which serve as the basic governing equations for fluid motion. The suitable numerical method for solving the new system of ordinary differential equations is the Runge–Kutta fourth-order numerical method with the shooting technique using the code MATLAB program. Using this code, a 2D and 3D graphical analysis was done to show how each physical parameter affected the distributions of axial velocity, temperature, nanoparticle volume fraction, solutal concentration, pressure gradients, induced magnetic field, magnetic forces, and finally the trapping phenomenon. Under the influence of rotation $\Omega$, heat Grashof number ${Gr}_{t}$, solutal Grashof number ${Gr}_{t}$, and initial stress ${P}^{*}$, the axial velocity distribution $u$ changes from increasing to decreasing, according to some of the study’s findings. On the other hand, increasing values of nonlinear thermal radiation $R$ and temperature ratio ${\theta }_{w}$ have a negative impact on the temperature distribution $\theta$ but a positive impact on the distributions of nanoparticle volume fraction $\Phi$ and solutal concentration $\gamma$. Darcy number $Da$ and mean fluid rate $F$ also had a positive effect on the distribution of pressure gradients, making it an increasing function.

## Introduction

The component of peristaltic pumping or the peristaltic movement of liquids inside living frameworks is perhaps the main strategy that certainly stands out to analysts in the field of biomechanics for its extraordinary significance and the significant job it plays inside the body of the creature. Latham^1^ was the first to study the transmission of fluids by the peristaltic pumping mechanism or the peristaltic wave method. Peristaltic pumping of fluids through flexible tubes is defined as the movement of fluids within these channels utilizing pressure gradients that push them making them move along these tubes, such as the movement of blood within the veins and arteries, the movement of food within the flexible esophageal channel, as well as the movement of food within the small and large intestines. On the other hand, urine moves from the kidney to the urinary bladder through the peristaltic pumping of urine through the flexible ureteral canal, as well as the movement of semen from the testicle to the outside through peristaltic pumping through the canal of the carrier vessel and many other forms of peristaltic movement within living systems. Akram and Razia^2^ concentrated on the crossover impacts of warm and focus convection on the peristaltic stream of fourth-grade nanofluids in an inclined tapered channel. According to the findings of this study, as the Brownian motion coefficient values rise, so does the fluid's temperature distribution. Kothandapani et al.^3^ investigated magnetic field impact on the peristaltic flow of a fourth-grade fluid in a tapered asymmetric channel. The impact of the magnetic field on the axial velocity distribution is nonuniform and changes among increment and reduction, as indicated by the aftereffects of this study. Abdulhadi and Ahmed^4^ examined the peristaltic flow of Walters-B fluid through a porous medium in a tapered asymmetric channel in the present magnetic field. It was found through this study that the temperature distribution turned into a rising function affected by the thermophoresis coefficient and the Dufour coefficient while the solute concentration distribution became a decreasing function under the influence of the Brownian motion coefficient and the Soret coefficient. Asha and Deepa^5^ analyzed the Joule heating and magnetic field effect on the peristaltic flow of a third-grade fluid in an asymmetric channel. Ranjit et al.^6^ illustrated Joule heating and zeta potential effect on peristaltic blood flow through porous micro-vessels altered by electrohydrodynamics. It was found from this study that the effect of the Brinkman number on the temperature distribution was positive.

It is realized that non-Newtonian liquids are one of the kinds of liquids that have an extraordinary nature, which is that they consolidate consistency and flexibility in their properties together. The viscosity of non-Newtonian fluids, on the other hand, varies depending on other factors like pressure and temperature. As a result, the viscosity of each model of non-Newtonian fluid is unique because the relationship between stress and strain for these fluids is nonlinear. In actuality, non-Newtonian fluids respond to external factors in a significant way. At the point when this liquid is exposed to a bunch of outside drives, it abandons a fluid substance to an adaptable, rubbery strong and when these powers are debilitated, it gets back to what it was previously. Non-Newtonian liquids assume a significant part in most significant applications that rely upon their properties including non-Newtonian liquid erosion decrease, oil-pipeline grating decrease, surfactant applications to huge scope warming and cooling frameworks, and stream tracers. It is additionally utilized in numerous enterprises, for example, paints, toothpaste, glue, lubricants, car oils, and foodstuffs such as ketchup and others. As a result, numerous researchers attempt to investigate the flow issues of various non-Newtonian fluids; El-Dabe et al.^7^ used a numerical approach to solve the MHD peristaltic flow of a non-Newtonian power-law nanofluid through a non-Darcy-porous-medium-inside-non-uniform inclined channel. This work found that the Darcy number and Reynolds number had a negative effect on the axial velocity distribution. Sayed et al.^8^ studied the peristaltic transport of nano non-Newtonian fluid with heat transfer through an inclined asymmetric duct with slip and convective boundary conditions. It was observed that the axial velocity distribution changed irregularly between decreasing and increasing under the influence of heat Grashof number one of the outcomes of that study. Ali et al.^9^ investigated the impact of the Hartmann boundary layer in peristaltic flow for viscoelastic fluid. Nadeem et al.^10^ provided an elliptic cross-sectional illustration of the peristaltic flow of a heated Jeffrey fluid within a duct that study revealed that an increase in the axial velocity distribution was caused by the mean flow rate parameter. Akram and Saleem^11^ discussed the analysis of heating effects and different waveforms on the peristaltic flow of Carreau fluid in a rectangular duct. It was found that the effect of the mean flow rate coefficient on the axial velocity distribution is positive, one of the outcomes of that study. Abbas et al.^12^ debated the peristaltic flow of Casson fluid with heat and mass transfer in the presence of Soret and Dufour effects with Hall currents through a non-Darcy porous medium inside a vertical channel. According to the findings of this study, both the temperature distribution and the nanoparticles volume fraction distribution were affected negatively by the Grashof thermal number. A peristaltic blood flow of non-Newtonian Carreau fluid with heat transfer phenomenon through a curved channel was discussed by Tanveer et al.^13^. The results of this study indicate that the axial velocity distribution is positively influenced by the Darcy number and the heat Grashof number. He and Mostapha^14^ agitated the significance of the Hall current and Joule heating impacts on Darcy–Forchheimer's peristaltic flow of a Rabinowitsch fluid through a tapered tube.

A substance with pores (voids) is known as a porous medium or porous material. The material's skeletal component is frequently referred to as the matrix or frame. The pores are normally loaded up with a liquid (fluid or gas). The skeletal material is typically strong, however, structures like froths are many times additionally helpfully investigated utilizing the idea of permeable media. A permeable medium is most frequently described by its porosity. Different properties of the medium (e.g., penetrability, rigidity, electrical conductivity) can in some cases get from the particular properties of its constituents (strong lattice and liquid) and the media porosity and pores structure, however, such a deduction is normally complicated. The idea of porous media, which is utilized in a lot of applied science and engineering fields: filtration, mechanics (acoustics, geomechanics, soil mechanics, rock mechanics), designing (oil designing, bio-remediation, development designing), geosciences (hydrogeology, petrol topography, geophysics), science and biophysics, material science, and so on. Liquid course through permeable media is a subject of most normal interest and has arisen in a different field of study. On the other hand, any natural and industrial areas are very interested in the flow through porous media. Models remember the progression of hydrocarbons for oil wells, groundwater courses through beds of rocks, as well as the vehicle of minerals and pollutants through the ground. One may likewise find this stream peculiarity straightforwardly significant in vaporous or fluid synergist and idle pressed bed reactors moves through separates channels, geothermal intensity the executives, softening, or cementing of double composites. Abou-zeid and Ouaf^15^ demonstrated the hall currents effect on squeezing the flow of non-Newtonian nanofluid through a porous medium between two parallel plates. Mccash et al.^16^ elucidated entropy analysis of the peristaltic flow of a hybrid nanofluid inside an elliptic duct with sinusoidally advancing boundaries. Ahmed et al.^17^ explained the effects of nonlinear thermal radiation, convective boundary conditions, and heat generation/absorption on magnetohydrodynamic Maxwell nanofluid flow over a stretching surface through a porous medium. It was found from this study that the effects of the magnetic field coefficient and Darcy number on the velocity distribution is negative. Mohamed et al.^18^ analyzed the nonlinear thermal radiation and heat generation/absorption effect on MHD Jeffrey nanofluid flow over a stretching sheet through a porous medium. This study included that the nanoparticles volume fraction distribution was an increasing function under the influence of the temperature ratio coefficient, while it became a decreasing function under the influence of the Brownian motion coefficient. The influences of thermal radiation and slip conditions on MHD Casson nanofluid flow over a stretching surface through a porous medium was studied by Mohamed et al.^19^. The temperature distribution in this study has become in a state of continuous increase under the influence of both the absorption and heat generation coefficient, as well as the thermal transfer coefficient. Bouslimi et al.^20^ dissected MHD Williamson nanofluid flow over a stretching sheet through a porous medium under the effects of Joule heating, nonlinear thermal radiation, heat generation/absorption, and chemical reaction. The velocity distribution decreases with an increase in the magnetic field coefficient, while the temperature distribution increases with an increase in this parameter, and this is one of the outcomes of that study. Rashed and Ahmed^21^ studied the peristaltic flow of dusty nanofluids in curved channels. This study proved that the heat Grashof number affects positively on the temperature distribution and negatively on the nanoparticles volume fraction distribution, while the effect of the nanoparticles Grashof number is the opposite. Ahmed and Rashed^22^ investigated magnetohydrodynamic dusty hybrid nanofluid peristaltic flow in curved channels. Mohamed et al.^23^ analyzed the thermal radiation and MHD effects on the free convective flow of a polar fluid through a porous medium in the presence of internal heat generation and chemical reaction. Abd-Alla et al.^24^ studied the effects of rotation and initial stress on peristaltic transport of fourth-grade fluid with heat transfer and induced magnetic field. The study shows that the axial velocity distribution decreases with an increase in the rotation coefficient, and increases with the enhancement in the heat generation and absorption coefficient. Abd-Alla et al.^25^ investigated the influence of heat and mass transfer, initial stress, and radially varying magnetic fields on the peristaltic flow in an annulus with a gravity field. Abd-Alla et al.^26^ illustrated the effect of rotation on the peristaltic flow of a micropolar fluid through a porous medium with an external magnetic field. Abd-Alla and Abo-Dahab^27^ elaborated on a magnetic field and rotation effects on the peristaltic transport of a Jeffrey fluid in an asymmetric channel. Abd-Alla et al.^28^ illustrated the peristaltic flow of a Jeffrey fluid under the effect of a radially varying magnetic field in a tube with an endoscope.

Nanofluids are a new class of fluids that was discovered by Choi and Eastman^29^. They are a basic fluid that is permeated with tiny metal particles called nanoparticles which are measured in nanometers. On the other hand, nanofluids play an important role in many modern technological applications as they are the most intelligent type of liquid. In the past few years, many researchers in the field of nanofluids have worked hard to understand the behavior of these fluids. From previous investigations, nanofluids have been found to possess enhanced thermophysical properties such as thermal conductivity, thermal diffusivity, viscosity, and convective heat transfer coefficients compared to those of base fluids like oil or water. Nanofluids can be used in a plethora of engineering applications ranging from use in the automotive industry to the medical arena to use in power plant cooling systems as well as computers. Ibrahim and Anbessa^30^ examined the three-dimensional MHD mixed convection flow of Casson nanofluid with hall and ion slip effects. Hayat et al.^31^ examined the impacts of slip in radiative MHD peristaltic flow of fourth-grade nanomaterial with chemical reaction. It was noted from the consequences of this study that the impact of the magnetic field coefficient on the axial velocity is negative, the temperature distribution has turned into a diminishing function affected by the non-linear thermal radiation coefficient, and the nanoparticles volume fraction diminishes with an expansion in the thermophoresis coefficient. Akram and Razia^32^ analyzed the hybrid effects of thermal and concentration convection on the peristaltic flow of fourth-grade nanofluids in an inclined tapered channel. A portion of the consequences of this study incorporated that the impact of the magnetic field coefficient on the axial velocity distribution was negative, and the temperature dispersion was a rising function affected by the thermophoresis coefficient. Kothandapani et al.^33^ discussed the effect of a magnetic field on the peristaltic flow of a fourth-grade fluid in a tapered asymmetric channel. It has been observed that the axial velocity distribution fluctuates between an increase and a decrease under the influence of the magnetic field coefficient. Haroun^34^ explained the non-linear peristaltic flow of a fourth-grade fluid in an inclined asymmetric channel. Akram et al.^35^ illustrated slip impact on double diffusion convection of magneto fourth-grade nanofluids with peristaltic propulsion through an inclined asymmetric channel. Hayat and Noreen^36^ investigated the peristaltic transport of fourth-grade fluid with heat transfer and an induced magnetic field. A portion of the consequences of this study showed that the heat Grashof number affects irregularly the axial velocity distribution and the induced magnetic field distribution. Mohamed et al.^37^ studied the MHD three-dimensional flow of a couple of stress nanofluids over a stretching sheet through a porous medium in the presence of heat generation/absorption and nonlinear thermal radiation. Abo-Dahab et al.^38^ discussed the Double-diffusive peristaltic MHD Sisko nanofluid flow through a porous medium in the presence of non-linear thermal radiation, heat generation/ absorption, and Joule heating. Abd-Alla et al.^39^ examined the rotation and initial stress effect on MHD peristaltic flow of reacting radiating fourth-grade nanofluid with viscous dissipation and Joule heating. Some results of this study showed that the distribution of axial velocity changes its effect between increase and decrease under the influence of rotation, initial stress, and magnetic field coefficient. Mohamed et al.^40^ analyzed the effects of nonlinear thermal radiation and heat generation/absorption on magnetohydrodynamic Carreau nanofluid flow on a nonlinear stretching surface through a porous medium. Raja et al.^41^ elaborated the integrated intelligent computing application for the effectiveness of Au nanoparticles coated over MWCNTs with velocity slip in curved channel peristaltic flow. Abdelhafez et al.^42^ illustrated the influence of an inclined magnetic field and heat and mass transfer on the peristaltic flow of blood in an asymmetric channel. Rafiq et al.^43^ studied the theoretical exploration of thermal transportation with Lorentz force for a fourth-grade fluid model obeying peristaltic mechanism. Abbas et al.^44^ investigated the rheology of peristaltic flow in a couple of stress fluids in an inclined tube. Abbas et al.^45^ analyzed the peristaltic transport of a Casson fluid in a non-uniform inclined tube with Roseland approximation and wall properties. Abbas and Rafiq^46^ examined the numerical simulation of thermal transportation with viscous dissipation for a peristaltic mechanism of micropolar-Casson fluid. Ghazanfari et al.^47^ sicussed the numerical study on the thermal performance of the shell and tube heat exchanger using twisted tubes and Al_2_O_3_ nanoparticles. Manh et al.^48^ elaborated the investigation of nanomaterial flow through non-parallel plates. Sheikholeslami et al.^49^ illustrated hybrid nanoparticles dispersion into water inside a porous wavy tank involving magnetic force. Awais et al.^50^ examined the MHD effects on ciliary-induced peristaltic flow coatings with rheological hybrid nanofluid, Ali et al.^51^ explained the entropy generation on MHD peristaltic flow of Cu–water nanofluid with slip conditions. Rooman et al.^52^ investigated the entropy generation analysis of magnetized radiative Ellis (Cu–TiO_2_/Engine Oil) nanofluid flow using Cattaneo–Christov heat flux model with viscous dissipation and Joule heating effects. Jameel et al.^53^ elaborated the statistical and entropy optimization modeling for radiative hybrid nanofluid flow with Hall effect over exponential stretching/shrinking plate. Asghar et al.^54^ analyzed the dual solutions of convective rotating flow of three-dimensional hybrid nanofluid across the linear stretching/shrinking sheet.

The double-diffusion of peristaltic flow of a non-Newtonian fourth-grade nanofluid through a vertically symmetric flexible channel through a porous medium was the subject of our investigation in this paper. The primary objective of this numerical study is to investigate the effects of rotation, initial stress, and induced magnetic field in the presence of non-linear thermal radiation, heat generation/absorption, and Joule heating with viscous dissipation. For the system of equations governing the peristaltic flow process, it is a set of partial differential equations that consist of the continuity equation, the momentum equation, the energy equation in addition to the solute concentration equation, the nanoparticles volume fraction equation, and the magnetic forces equation, and induced magnetic field equation. The limitations of the long-wavelength approximation were used to facilitate the solution of the aforementioned system of differential equations and thus the system of these differential equations was converted to the system of ordinary differential equations which was solved numerically using the fourth-order Runge–Kutta method with the shooting technique by MATLAB code. The effect of all important physical parameters resulting from the current study on important distributions such as velocity, temperature, solute concentration, nanoparticle volume fraction, stream function, and induced magnetic field was studied employing the graphs using the MATLAB program and the results are discussed with the physical meaning of the phenomenon.

## Discerption of the problem and mathematical formulation

**Figure 1:**
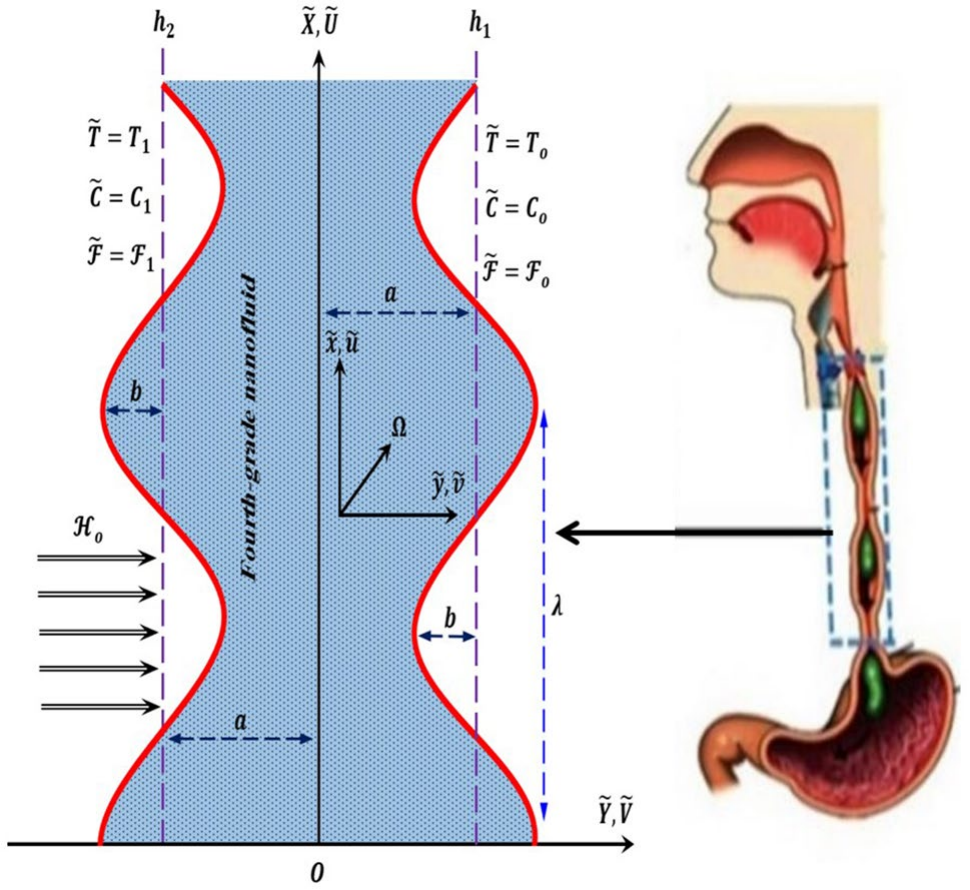
The geometry of the problem.

At the outset, it should be noted that the current numerical study is represented in the peristaltic motion of fluid inside the vertical symmetrical flexible channel called a fourth-grade nanofluid, A fourth-grade fluid is defined as a category of non-Newtonian fluids with very high viscosity and describe the shear thinning and shear thickening phenomena which cannot be expressed by the classical Navier–Stokes equations, and this study is a simulation of the movement of food and liquids from the mouth to the stomach through the flexible esophageal channel, food, and liquids work on the contraction and relaxation of the esophageal channel walls, which is what happens to the fourth-grade fluid movement inside the flexible channel used in the study additionally, the study of the model's non-Newtonian fluid peristaltic motion's natural properties in general. It is worth noting that the peristaltic movement of the fluid inside the channel occurs in the presence of a suitable porous medium with permeable properties that acts as a resistance force that affects the movement of the fluid inside the channel. The porous medium contains a group of holes or voids permeated by the fluid during its movement, In the simulation or applied study, it corresponds to the membrane lining the walls of the flexible esophageal channel, as it is a semi-permeable membrane, or food can be considered as a porous medium permeated with fluids also the size of the pores of the porous medium is appropriate to the size of the nanoparticles and that the mechanism of action of the porous medium is subject to Darcy's law, see^55^. Consider the magnetohydrodynamic (MHD) peristaltic steady flow of an incompressible fourth-grade nanofluid through a porous medium in a vertical two-dimensional symmetric channel of uniform thickness $2a$ subjected to Joule heating, initial stress, induced magnetic field, and rotation in presence of nonlinear thermal radiation, heat generation/absorption, and viscous dissipation, the fluid flow in the channel walls is produced, when the sinusoidal waves of the small amplitude $b$ propagate the speed of the channel walls. $c$ is the constant speed of the channel. In this current study, the Cartesian coordinate system will be used and will be selected $\widetilde{X}$ in the direction of wave propagation and $\widetilde{Y}$ transverse to it, see Fig. 1. A constant magnetic field of strength ${\mathcal{H}}_{o}$ acting in the transverse direction results in an induced magnetic field and the total magnetic is $\mathcal{H}\left({\widetilde{h}}_{\widetilde{X}}\left(\widetilde{X},\widetilde{Y},\widetilde{t}\right),{\widetilde{h}}_{\widetilde{X}}\left(\widetilde{X},\widetilde{Y},\widetilde{t}\right),0\right),{\mathcal{H}}^{+}\left({\widetilde{h}}_{\widetilde{X}}\left(\widetilde{X},\widetilde{Y},\widetilde{t}\right), {\zeta }_{o}+{\widetilde{h}}_{\widetilde{X}}\left(\widetilde{X},\widetilde{Y},\widetilde{t}\right),0\right).$

The geometry of the symmetric channel can be written as follows^56^;

1 $${\widetilde{h}}_{1}\left(\widetilde{X},\widetilde{t}\right)=b+a\mathrm{cos}\left(\frac{2\pi }{\lambda }\left(\widetilde{X}-c\widetilde{t}\right)\right),$$

2 $${\widetilde{h}}_{2}\left(\widetilde{X},\widetilde{t}\right)=-b-a\mathrm{cos}\left(\frac{2\pi }{\lambda }\left(\widetilde{X}-c\widetilde{t}\right)\right).$$

The velocity field $\overrightarrow{q}$ in the two-dimensional flow can be written as follows;

3 $$\overrightarrow{q}=\left(\widetilde{U},\widetilde{V},0\right).$$

4 $$\widetilde{U}=\widetilde{U}\left(\widetilde{X},\widetilde{Y},0\right),\widetilde{V}= \widetilde{V}\left(\widetilde{X},\widetilde{Y},0\right)$$

The governing equations of the non-Newtonian nanofluid motion can be written as follows^24,39,56^;

5 $$\nabla \cdot \overrightarrow{q}=0,$$

6 $$\begin{aligned}{\rho }_{f}\frac{\partial \overrightarrow{q}}{\partial \widetilde{t}}&+{\rho }_{f}\left[\overrightarrow{\Omega }\times \left(\overrightarrow{\Omega }\times \overrightarrow{q}\right)+2\overrightarrow{\Omega }\times \overrightarrow{q}\right]\\&=div\left(\overline{\mathbf{T} }\right)+{\mu }_{e}\left[\left(\nabla \times {\mathcal{H}}^{+}\right)\times {\mathcal{H}}^{+}-\nabla {{\mathcal{H}}^{+}}^{2}/2\right]\\ &\quad +\left(1-{C}_{o}\right){\rho }_{f}g\left[{\beta }_{T}\left(\widetilde{T}-{T}_{o}\right)+{\beta }_{\mathcal{F}}\left(\widetilde{\mathcal{F}}-{\mathcal{F}}_{o}\right)\right]-g\left({\rho }_{p}-{\rho }_{f}\right)\left(\widetilde{C}-{C}_{o}\right)-\frac{\mu }{{K}_{p}}\overrightarrow{q}\\& \quad +{P}^{*}\nabla {\omega }_{3},\end{aligned}$$

7 $$\begin{aligned}{\left(\rho {c}_{P}\right)}_{f}\left[\frac{\partial \widetilde{T}}{\partial \widetilde{t}}+\overrightarrow{q}\cdot \nabla \widetilde{T}\right]&=\nabla \cdot \left(k\nabla \widetilde{T}\right)+{\left(\rho {c}_{P}\right)}_{p}\left[{D}_{B}\nabla \widetilde{C}\cdot \nabla \widetilde{T}+{D}_{T}\frac{\nabla \widetilde{T}\cdot \nabla \widetilde{T}}{{T}_{m}}\right]\\& \quad +\nabla \cdot \left({D}_{T\mathcal{F}}\nabla \widetilde{\mathcal{F}}\right)+{Q}_{o}\left(\widetilde{T}-{T}_{o}\right)+\nabla \cdot {\overrightarrow{q}}_{r}{+\mu \nabla {q}^{2}+J}_{he},\end{aligned}$$

8 $$\frac{\partial \widetilde{\mathcal{F}}}{\partial t}+\overrightarrow{q}\cdot \nabla \widetilde{\mathcal{F}}=\nabla \cdot \left[{D}_{S}\nabla \widetilde{\mathcal{F}}+{D}_{\mathcal{F}T}\nabla \widetilde{T}\right],$$

9 $$\frac{\partial \widetilde{C}}{\partial t}+\overrightarrow{q}\cdot \nabla \widetilde{C}=\nabla \cdot \left[{D}_{B}\nabla \widetilde{C}+{D}_{T}\frac{\nabla \widetilde{T}}{{T}_{o}}\right],$$

10 $$\frac{d{\mathcal{H}}^{+}}{dt}=\nabla \times \left(\overrightarrow{q}\times {\mathcal{H}}^{+}\right)+\frac{1}{\varsigma }{\nabla }^{2}{\mathcal{H}}^{+}.$$

where the Eqs. (5)–(10) in general are the continuity equation, the momentum equation, the energy equation, the solute concentration equation, the nanoparticles volume fraction equation, and the magnetic force equation respectively.

Cauchy stress tensor $\overline{\mathbf{T} }$ for the non-Newtonian fourth-grade fluid can be written as follows^24^:

11 $$\begin{aligned}\overline{\mathbf{T} }&=-P\overline{I }+\mu {\overline{A} }_{1}+{\alpha }_{1}{\overline{A} }_{2}+{\alpha }_{2}{\left({\overline{A} }_{1}\right)}^{2}+{\beta }_{1}{\overline{A} }_{3}+{\beta }_{2}\left[{\overline{A} }_{1}{\overline{A} }_{2}+{\overline{A} }_{2}{\overline{A} }_{1}\right]+{\beta }_{3}\left[tr\left({{\overline{A} }_{1}}^{2}\right){\overline{A} }_{1}\right]+{\gamma }_{1}{\overline{A} }_{4}\\&\quad +{\gamma }_{2}\left[{\overline{A} }_{1}{\overline{A} }_{3}+{\overline{A} }_{3}{\overline{A} }_{1}\right]+{\gamma }_{3}\left[{\left({\overline{A} }_{2}\right)}^{2}\right]+{\gamma }_{4}\left({\overline{A} }_{2}{\left({\overline{A} }_{1}\right)}^{2}+{\left({\overline{A} }_{1}\right)}^{2}{\overline{A} }_{2}\right)+{\gamma }_{5}\left[\left(tr\left({\overline{A} }_{2}\right){\overline{A} }_{2}\right)\right]\\&\quad +{\gamma }_{6}\left[tr\left({\overline{A} }_{2}\right){\left({\overline{A} }_{1}\right)}^{2}\right]+\left[{\gamma }_{7}\left(tr\left({\overline{A} }_{3}\right)\right)+{\gamma }_{8}\left(tr\left({\overline{A} }_{2}{\overline{A} }_{1}\right)\right)\right]{\overline{A} }_{1}.\end{aligned}$$

where ${\alpha }_{i} (i=1, 2)$,${\beta }_{i }(i=1, 2, 3)$ and ${\gamma }_{i} \left(i=\mathrm{1,2},\dots ,8\right)$, The Rivlin–Ericksen tensors ${\overline{A} }_{i} (i=1 to 4)$ are defined as follows^24,39,56^:

12 $${\overline{A} }_{1}=\left(\nabla \overrightarrow{q}\right)+{\left(\nabla \overrightarrow{q}\right)}^{\widetilde{\tau }},$$

13 $${\overline{A} }_{i}=\frac{d}{dt}{\overline{A} }_{i-1}+{\overline{A} }_{i-1}\left(\nabla \overrightarrow{q}\right)+{\left(\nabla \overrightarrow{q}\right)}^{\widetilde{\tau }}{\overline{A} }_{i-1}, i=\mathrm{2,3},4,$$

In magnetic fluid mechanics Maxwell’s equations in the absence of displacement current are presented as follows^24^;

14 $$\nabla .\overrightarrow{B}=0, \nabla .\overrightarrow{D}=0, \nabla \times \overrightarrow{\mathcal{H}}=\overrightarrow{J}, \nabla \times \overrightarrow{E}=-\frac{\partial \overrightarrow{B}}{\partial t},\overrightarrow{B}={\mu }_{e}{\overrightarrow{\mathcal{H}}}_{o},\overrightarrow{D}={\varepsilon }_{r}\overrightarrow{E},\overrightarrow{J}=\sigma \left(\overrightarrow{E}+\overrightarrow{q}\times \overrightarrow{B}\right).$$

Modified Darcy’s law obeys^55^

15 $${R}_{K}=-\frac{{\varepsilon }^{*}}{{K}_{P}}{\mu }_{app}\overrightarrow{q}.$$

Now, $\left(\widetilde{x},\widetilde{y}\right)$ represent the wave frame moving with velocity $c$ away from the fixed frame $\left(\widetilde{X},\widetilde{Y}\right)$ by the transformations^24^;

16 $$\widetilde{x}=\widetilde{X}-c\widetilde{t}, \quad \widetilde{y}=\widetilde{Y}, \quad \widetilde{u}\left(\widetilde{x},\widetilde{y}\right)=\widetilde{U}-c, \quad \widetilde{v}\left(\widetilde{x},\widetilde{y}\right)=\widetilde{V,}$$

Now, the basic governing equations of MHD non-Newtonian fourth-grade nanofluid can be written mathematically as follows^24,39,48^;

17 $$\frac{\partial \widetilde{u}}{\partial \widetilde{x}}+\frac{\partial \widetilde{v}}{\partial \widetilde{y}}=0,$$

18 $$\begin{aligned}\rho \left(\widetilde{u}\frac{\partial \widetilde{u}}{\partial \widetilde{x}}+\widetilde{v}\frac{\partial \widetilde{u}}{\partial \widetilde{y}}\right)&=-\frac{\partial P}{\partial \widetilde{x}}+\rho {\Omega }^{2}\widetilde{u}+\frac{\partial {\overline{S} }_{xx}}{\partial x}+\frac{\partial {\overline{S} }_{xy}}{\partial y}-\frac{{\mu }_{e}}{2}\left(\frac{\partial {{\mathcal{H}}^{+}}^{2}}{\partial \widetilde{x}}\right)\\&\quad +{\mu }_{e}\left({\widetilde{h}}_{\widetilde{x}}\frac{\partial {\widetilde{h}}_{y}}{\partial \widetilde{x}}+{\widetilde{h}}_{\widetilde{y}}\frac{\partial {\widetilde{h}}_{y}}{\partial \widetilde{y}}+{\mathcal{H}}_{o}\frac{\partial {\widetilde{h}}_{y}}{\partial \widetilde{y}}\right)+{P}^{*}\frac{\partial }{\partial \widetilde{y}}\left(\frac{\partial \widetilde{v}}{\partial \widetilde{x}}-\frac{\partial \widetilde{u}}{\partial \widetilde{y}}\right)\\&\quad +\left(1-{C}_{o}\right)\rho g\left({\beta }_{T}\left(\widetilde{T}-{T}_{o}\right)+{\beta }_{c}\left(\widetilde{\mathcal{F}}-{\mathcal{F}}_{o}\right)\right)-g\left(\rho -{\rho }_{p}\right)\left(\widetilde{C}-{C}_{o}\right)-\frac{\mu }{{K}_{p}}\widetilde{u},\end{aligned}$$

19 $$\begin{aligned}\rho \left(\widetilde{u}\frac{\partial \widetilde{v}}{\partial \widetilde{x}}+\widetilde{v}\frac{\partial \widetilde{v}}{\partial \widetilde{y}}\right)&=-\frac{\partial P}{\partial \widetilde{y}}+\rho {\Omega }^{2}\widetilde{v}+\frac{\partial {\overline{S} }_{xy}}{\partial \widetilde{x}}+\frac{\partial {\overline{S} }_{yy}}{\partial \widetilde{y}}-\frac{{\mu }_{e}}{2}\left(\frac{\partial {{\mathcal{H}}^{+}}^{2}}{\partial \widetilde{y}}\right)\\&\quad +{\mu }_{e}\left({\widetilde{h}}_{\widetilde{x}}\frac{\partial {\widetilde{h}}_{y}}{\partial \widetilde{x}}+{\widetilde{h}}_{\widetilde{y}}\frac{\partial {\widetilde{h}}_{y}}{\partial \widetilde{y}}+{\mathcal{H}}_{o}\frac{\partial {\widetilde{h}}_{y}}{\partial \widetilde{y}}\right)+{P}^{*}\frac{\partial }{\partial \widetilde{x}}\left(\frac{\partial \widetilde{v}}{\partial \widetilde{x}}-\frac{\partial \widetilde{u}}{\partial \widetilde{y}}\right)-\frac{\mu }{{K}_{p}}\widetilde{v},\end{aligned}$$

20 $$\begin{aligned}{\left(\rho {c}_{P}\right)}_{f}\left(\widetilde{u}\frac{\partial \widetilde{T}}{\partial \widetilde{x}}+\widetilde{v}\frac{\partial \widetilde{T}}{\partial \widetilde{y}}\right)&=k\left(\frac{{\partial }^{2}\widetilde{T}}{\partial {\widetilde{x}}^{2}}+\frac{{\partial }^{2}\widetilde{T}}{\partial {\widetilde{y}}^{2}}\right)+{\left(\rho {c}_{P}\right)}_{p}\left(\begin{array}{c}{D}_{B}\left(\frac{\partial \widetilde{C}}{\partial \widetilde{x}}\frac{\partial \widetilde{T}}{\partial \widetilde{x}}+\frac{\partial \widetilde{C}}{\partial \widetilde{y}}\frac{\partial \widetilde{T}}{\partial \widetilde{y}}\right)\\ +\left(\frac{{D}_{T}}{{T}_{\infty }}\right)\left({\left(\frac{\partial \widetilde{T}}{\partial \widetilde{x}}\right)}^{2}+{\left(\frac{\partial \widetilde{T}}{\partial \widetilde{y}}\right)}^{2}\right)\end{array}\right) \\ &\quad+{D}_{T\mathcal{F}}\left(\frac{{\partial }^{2}\widetilde{\mathcal{F}}}{\partial {\widetilde{x}}^{2}}+\frac{{\partial }^{2}\widetilde{\mathcal{F}}}{\partial {\widetilde{y}}^{2}}\right)+{Q}_{o}\left(\widetilde{T}-{T}_{o}\right)+\sigma {B}_{o}^{2}{\widetilde{u}}^{2}+\mu {\left(\frac{\partial \widetilde{u}}{\partial \widetilde{y}}\right)}^{2}-\frac{\partial {q}_{r}}{\partial \widetilde{y}},\end{aligned}$$

21 $$\widetilde{u}\frac{\partial \widetilde{\mathcal{F}}}{\partial \widetilde{x}}+\widetilde{v}\frac{\partial \widetilde{\mathcal{F}}}{\partial \widetilde{y}}={D}_{S}\left(\frac{{\partial }^{2}\widetilde{\mathcal{F}}}{\partial {\widetilde{x}}^{2}}+\frac{{\partial }^{2}\widetilde{\mathcal{F}}}{\partial {\widetilde{y}}^{2}}\right)+{D}_{\mathcal{F}T}\left(\frac{{\partial }^{2}\widetilde{T}}{\partial {\widetilde{x}}^{2}}+\frac{{\partial }^{2}\widetilde{T}}{\partial {\widetilde{y}}^{2}}\right),$$

22 $$\widetilde{u}\frac{\partial \widetilde{C}}{\partial \widetilde{x}}+\widetilde{v}\frac{\partial \widetilde{C}}{\partial \widetilde{y}}={D}_{B}\left(\frac{{\partial }^{2}\widetilde{C}}{\partial {\widetilde{x}}^{2}}+\frac{{\partial }^{2}\widetilde{C}}{\partial {\widetilde{y}}^{2}}\right)+\left(\frac{{D}_{T}}{{T}_{o}}\right)\left(\frac{{\partial }^{2}\widetilde{T}}{\partial {\widetilde{x}}^{2}}+\frac{{\partial }^{2}\widetilde{T}}{\partial {\widetilde{y}}^{2}}\right).$$

where Eqs. (17)–(22) represent continuity equation, momentum equation, energy equation, solutal concentration equation, nanoparticles volume fraction equation respectively the wave frame $\left(\widetilde{x},\widetilde{y}\right)$, ${q}_{r}=-4{\sigma }^{*}\left(\frac{\partial {\widetilde{T}}^{4}}{\partial \widetilde{y}}\right)/3{k}^{*}$ and ${\omega }_{3}=\frac{\partial v}{\partial x}-\frac{\partial u}{\partial y}$.

Using non-dimensional quantities as follows;

$$\begin{aligned} & \widetilde{x}=x\lambda ,\quad \widetilde{y}=ya,\quad P=\frac{{a}^{2}}{\lambda \mu c}\widetilde{P},\quad {S}_{ij}=\frac{a{\overline{S} }_{ij}}{\mu c},\quad u=\frac{\widetilde{u}}{c},\quad v=\frac{\widetilde{v}}{c},\quad k=\alpha {\left(\rho {c}_{p}\right)}_{f}\\&Hr=Re{S}^{2}{R}_{m},\quad Re=\frac{{d}_{1}c\rho }{\mu },\quad S=\sqrt{{\mathcal{H}}_{o}^{2}{\mu }_{e}/{c}^{2}\rho },\quad \phi =\frac{\widetilde{\phi }}{{d}_{1}{\mathcal{H}}_{o}},\quad {\widetilde{h}}_{\widetilde{x}}={\widetilde{\phi }}_{\widetilde{y}}, \\&Ec=\frac{{c}^{2}}{{c}_{P}\left({{T}_{1}-T}_{o}\right)},\quad Pr=\frac{\mu {c}_{P}}{k},\quad \theta =\frac{\widetilde{T}-{T}_{o}}{{T}_{1}-{T}_{o}},\quad {Gr}_{t}=\frac{\left(1-{C}_{o}\right)\rho {a}^{2}g{\beta }_{T}\left({{T}_{1}-T}_{o}\right)}{c\mu },\\ &\Phi =\frac{\widetilde{C}-{C}_{o}}{{{C}_{1}-C}_{o}},\quad Nb=\frac{{\left(\rho {c}_{P}\right)}_{p}{D}_{B}\left({{C}_{1}-C}_{o}\right)}{k},\quad Nt=\frac{{\left(\rho {c}_{P}\right)}_{p}{D}_{T}\left({{T}_{1}-T}_{o}\right)}{k{T}_{o}},\quad {\widetilde{h}}_{\widetilde{y}}=-{\widetilde{\phi }}_{\widetilde{x}},\\ &\delta =\frac{a}{\lambda },\quad {R}_{m}=\sigma ac{\mu }_{e},\quad E=-\frac{\widetilde{E}}{{\mathcal{H}}_{o}ac{\mu }_{e}},\quad \beta =\frac{{Q}_{o}{a}^{2}}{\mu {c}_{P}},\quad M=\frac{\sigma {B}_{o}^{2}{a}^{2}}{\mu },\quad { \theta }_{w}=\frac{{T}_{o}}{{{T}_{1}-T}_{o}}\\ &R=\frac{16{\sigma }^{*}{\left({{T}_{1}-T}_{o}\right)}^{3}}{3k{k}^{*}},\quad {Gr}_{p}=\frac{\left({\rho }_{np}-\rho \right){a}^{2}g\left({{C}_{1}-C}_{o}\right)}{c\mu },\quad Da=\frac{{a}^{2}}{{K}_{p}},\quad \gamma =\frac{\widetilde{\mathcal{F}}-{\mathcal{F}}_{o}}{{{\mathcal{F}}_{1}-\mathcal{F}}_{o}},\\ &{Gr}_{c}=\frac{\left(1-{C}_{o}\right)\rho {a}^{2}g{\beta }_{c}\left({\mathcal{F}}_{1}-{\mathcal{F}}_{o}\right)}{c\mu },\quad {N}_{\mathcal{F}T}=\frac{{D}_{\mathcal{F}T}\left({{T}_{1}-T}_{o}\right)}{{D}_{S}\left({{\mathcal{F}}_{1}-\mathcal{F}}_{o}\right)},\quad {N}_{T\mathcal{F}}=\frac{{D}_{T\mathcal{F}}\left({{\mathcal{F}}_{1}-\mathcal{F}}_{o}\right)}{k\left({{T}_{1}-T}_{o}\right)},\quad Bn=Ec\cdot Pr\end{aligned}$$

The stream function $\psi$, velocity fields, and the dimensionless axial induced magnetic field are defined as follows;

23 $$u=\frac{\partial \psi }{\partial y}, v=-\delta \frac{\partial \psi }{\partial x}, {h}_{x}=a{\mathcal{H}}_{o}\frac{\partial \phi }{\partial y}, {h}_{x}=-a{\delta \mathcal{H}}_{o}\frac{\partial \phi }{\partial x}.$$

Substituting Eq. (23) in Eqs. (18)–(22) will produce

24 $$\begin{aligned}Re\delta \left(\frac{\partial \psi }{\partial y}\frac{{\partial }^{2}\psi }{\partial x\partial y}-\frac{\partial \psi }{\partial x}\frac{{\partial }^{2}\psi }{\partial {y}^{2}}\right)&=-\frac{\partial P}{\partial x}+\frac{{\Omega }^{2}{a}^{2}\rho }{\mu }\frac{\partial \psi }{\partial y}+\delta \frac{\partial {S}_{xx}}{\partial x}+\frac{\partial {S}_{xy}}{\partial y}+\delta Re{S}^{2}{a}^{2}\left(\frac{\partial \phi }{\partial y}\frac{{\partial }^{2}\phi }{\partial x\partial y}-\frac{\partial \phi }{\partial x}\frac{{\partial }^{2}\phi }{\partial {y}^{2}}\right)\\&\quad +Re{S}^{2}{a}^{2}\frac{{\partial }^{2}\phi }{\partial {y}^{2}}-\frac{{p}^{*}}{\mu }\left({\delta }^{2}\frac{{\partial }^{3}\psi }{\partial y\partial {x}^{2}}+\frac{{\partial }^{3}\psi }{\partial {y}^{3}}\right)+{Gr}_{t}\theta +{Gr}_{c}\gamma -{Gr}_{p}\Phi -Da\frac{\partial \psi }{\partial y} ,\end{aligned}$$

25 $$\begin{aligned}Re{\delta }^{3}\left(\frac{\partial \psi }{\partial y}\frac{{\partial }^{2}\psi }{\partial x\partial y}-\frac{\partial \psi }{\partial x}\frac{{\partial }^{2}\psi }{\partial {y}^{2}}\right)&=-\frac{\partial P}{\partial y}+\frac{{\Omega }^{2}{a}^{2}\rho }{\mu }\frac{\partial \psi }{\partial x}+\delta \left(\delta \frac{\partial {S}_{xy}}{\partial x}+\frac{\partial {S}_{yy}}{\partial y}\right)+{\delta }^{3}Re{S}^{2}m\left(\frac{\partial \phi }{\partial y}\frac{{\partial }^{2}\phi }{\partial {x}^{2}}-\frac{\partial \phi }{\partial x}\frac{{\partial }^{2}\phi }{\partial x\partial y}\right)\\&\quad +{\delta }^{2}Re{S}^{2}{a}^{2}\frac{{\partial }^{2}\phi }{\partial x\partial y}-\frac{{\delta p}^{*}}{\lambda \mu }\left({\delta }^{2}\frac{{\partial }^{3}\psi }{\partial {x}^{3}}+\frac{{\partial }^{3}\psi }{\partial x\partial {y}^{2}}\right)+Da\delta \frac{\partial \psi }{\partial x},\end{aligned}$$

26 $$\begin{aligned}RePr\delta \left(\frac{\partial \psi }{\partial y}\frac{\partial \theta }{\partial x}-\frac{\partial \psi }{\partial x}\frac{\partial \theta }{\partial y}\right)&=\left({\delta }^{2}\frac{{\partial }^{2}\theta }{\partial {x}^{2}}+\frac{{\partial }^{2}\theta }{\partial {y}^{2}}\right)+Nb\left({\delta }^{2}\frac{\partial \theta }{\partial x}\frac{\partial\Phi }{\partial x}+\frac{\partial \theta }{\partial y}\frac{\partial\Phi }{\partial y}\right)+Bn M{\left(\frac{\partial \psi }{\partial y}\right)}^{2}+ \beta \theta \\&\quad + Nt\left({\delta }^{2}{\left(\frac{\partial \theta }{\partial x}\right)}^{2}+{\left(\frac{\partial \theta }{\partial y}\right)}^{2}\right)+ {N}_{T\mathcal{F}}\left({\delta }^{2}\frac{{\partial }^{2}\gamma }{\partial {x}^{2}}+\frac{{\partial }^{2}\gamma }{\partial {y}^{2}}\right)+Bn\left(\frac{{\partial }^{2}\psi }{\partial {y}^{2}}\right)\\&\quad + R{\left[{\left({\theta }_{w}+\theta \right)}^{3}\left(\frac{\partial \theta }{\partial y}\right)\right]}{\prime},\end{aligned}$$

27 $$Re\delta \left(\frac{\partial \psi }{\partial y}\frac{\partial\upgamma }{\partial x}-\frac{\partial \psi }{\partial x}\frac{\partial\upgamma }{\partial y}\right)=\left({\delta }^{2}\frac{{\partial }^{2}\upgamma }{\partial {x}^{2}}+\frac{{\partial }^{2}\upgamma }{\partial {y}^{2}}\right)+{N}_{\mathcal{F}T}\left({\delta }^{2}\frac{{\partial }^{2}\theta }{\partial {x}^{2}}+\frac{{\partial }^{2}\theta }{\partial {y}^{2}}\right),$$

28 $$Re\delta \left(\frac{\partial \psi }{\partial y}\frac{\partial\Phi }{\partial x}-\frac{\partial \psi }{\partial x}\frac{\partial\Phi }{\partial y}\right)=\left({\delta }^{2}\frac{{\partial }^{2}\Phi }{\partial {x}^{2}}+\frac{{\partial }^{2}\Phi }{\partial {y}^{2}}\right)+\frac{Nt}{Nb}\left({\delta }^{2}\frac{{\partial }^{2}\theta }{\partial {x}^{2}}+\frac{{\partial }^{2}\theta }{\partial {y}^{2}}\right),$$

29 $$E=-\delta \left(\frac{\partial \psi }{\partial y}\frac{\partial \phi }{\partial x}-\frac{\partial \psi }{\partial x}\frac{\partial \phi }{\partial y}\right)+\frac{1}{{R}_{m}}\left({\delta }^{2}\frac{{\partial }^{2}\phi }{\partial {x}^{2}}+\frac{{\partial }^{2}\phi }{\partial {y}^{2}}\right).$$

It is important to note that the values of each ${S}_{xx},{ S}_{xy}$, and ${S}_{yy}$ can be changed in Eqs. (11)–(13) respectively. The final form of the Eqs. (26)–(30) is as follows when using the long-wavelength approximation and disregarding the wave number and low Reynolds number in turn:

30 $$\frac{\partial P}{\partial x}=\frac{{\Omega }^{2}{a}^{2}\rho }{\mu }\frac{\partial \psi }{\partial y}+\frac{\partial {S}_{xy}}{\partial y}+Re{S}^{2}{a}^{2}\frac{{\partial }^{2}\phi }{\partial {y}^{2}}-\frac{{p}^{*}}{\mu }\left(\frac{{\partial }^{3}\psi }{\partial {y}^{3}}\right)+{Gr}_{t}\theta +{Gr}_{c}\gamma -{Gr}_{p}\Phi -Da\frac{\partial \psi }{\partial y},$$

31 $$\frac{\partial P}{\partial y}=0,$$

32 $$\frac{{\partial }^{2}\theta }{\partial {y}^{2}}=-\left(\begin{array}{c} Nb\left(\frac{\partial \theta }{\partial y}\frac{\partial\Phi }{\partial y}\right)+ Nt{\left(\frac{\partial \theta }{\partial y}\right)}^{2}+Bn M{\left(\frac{\partial \psi }{\partial y}\right)}^{2}+ \beta \theta \\ +Bn{\left(\frac{{\partial }^{2}\psi }{\partial {y}^{2}}\right)}^{2}+{N}_{T\mathcal{F}}\left(\frac{{\partial }^{2}\gamma }{\partial {y}^{2}}\right)+ R{\left({\left({\theta }_{w}+\theta \right)}^{3}\left(\frac{\partial \theta }{\partial y}\right)\right)}{\prime}\end{array}\right),$$

33 $$\frac{{\partial }^{2}\gamma }{\partial {y}^{2}}=-{N}_{\mathcal{F}T}\left(\frac{{\partial }^{2}\theta }{\partial {y}^{2}}\right),$$

34 $$\frac{{\partial }^{2}\Phi }{\partial {y}^{2}}=-\frac{Nt}{Nb}\left(\frac{{\partial }^{2}\theta }{\partial {y}^{2}}\right),$$

35 $$E=\frac{1}{{R}_{m}}\left(\frac{{\partial }^{2}\phi }{\partial {y}^{2}}\right).$$

The boundary conditions for the dimensionless in the wave frame are given as follows;

36 $$\psi =-\frac{F}{2},\; \frac{\partial \psi }{\partial y}=0,\; \theta =0,\; \Phi =0,\; \gamma =0,\; \phi =0,\; \mathrm{at} y={h}_{2}=-1-\varepsilon cos\left(2\pi x\right).$$

37 $$\psi =\frac{F}{2},\; \frac{\partial \psi }{\partial y}=0,\; \theta =1,\; \Phi =1,\; \gamma =1,\; \phi =1,\; \mathrm{at} y={h}_{1}=1+\varepsilon cos\left(2\pi x\right),$$

Now, the extra stress tensor for fourth-grade fluid can be written as follows^31^:

38 $${S}_{xy}=\mu \frac{{\partial }^{2}\psi }{\partial {y}^{2}}-{\alpha }_{1}\frac{{\partial }^{3}\psi }{\partial {y}^{3}}+{\beta }_{1}\frac{{\partial }^{4}\psi }{\partial {y}^{4}}+2{\beta }_{2}{\left(\frac{{\partial }^{2}\psi }{\partial {y}^{2}}\right)}^{3}-{\gamma }_{1}\frac{{\partial }^{5}\psi }{\partial {y}^{5}}-{\gamma }^{*}{\frac{{\partial }^{3}\psi }{\partial {y}^{3}}\left(\frac{{\partial }^{2}\psi }{\partial {y}^{2}}\right)}^{2}.$$

where ${\alpha }_{1},{\beta }_{1},{\beta }^{*},{\gamma }_{1}$ and ${\gamma }^{*}$ are material constants and $\mu$ is the coefficient of viscosity. Substituting Eq. (38) into Eq. (31) results in that

39 $$\begin{aligned}\frac{\partial P}{\partial x}&=\frac{{\Omega }^{2}{a}^{2}\rho }{\mu }\left(\frac{\partial \psi }{\partial y}\right)+\frac{\partial }{\partial y}\left(\begin{array}{c}\frac{{\partial }^{2}\psi }{\partial {y}^{2}}-{\alpha }_{1}\frac{{\partial }^{3}\psi }{\partial {y}^{3}}+{\beta }_{1}\frac{{\partial }^{4}\psi }{\partial {y}^{4}}+2{\beta }_{2}{\left(\frac{{\partial }^{2}\psi }{\partial {y}^{2}}\right)}^{3}\\ -{\gamma }_{1}\frac{{\partial }^{5}\psi }{\partial {y}^{5}}-{\gamma }^{*}{\frac{{\partial }^{3}\psi }{\partial {y}^{3}}\left(\frac{{\partial }^{2}\psi }{\partial {y}^{2}}\right)}^{2}\end{array}\right)+Re{S}^{2}E{R}_{m}-\frac{{p}^{*}}{\mu }\frac{{\partial }^{3}\psi }{\partial {y}^{3}}\\&\quad +{Gr}_{t}\theta +{Gr}_{c}\gamma -{Gr}_{p}\Phi -Da\frac{\partial \psi }{\partial y}.\end{aligned}$$

Thus, the final form of Eq. (39) becomes the form

40 $$\begin{aligned}\frac{\partial P}{\partial x}&=\frac{{\Omega }^{2}{a}^{2}\rho }{\mu }\frac{\partial \psi }{\partial y}+\mu \frac{{\partial }^{3}\psi }{\partial {y}^{3}}-{\alpha }_{1}\frac{{\partial }^{4}\psi }{\partial {y}^{4}}+{\beta }_{1}\frac{{\partial }^{5}\psi }{\partial {y}^{5}}+6{\beta }_{2}\frac{{\partial }^{3}\psi }{\partial {y}^{3}}{\left(\frac{{\partial }^{2}\psi }{\partial {y}^{2}}\right)}^{2}-{\gamma }_{1}\frac{{\partial }^{6}\psi }{\partial {y}^{6}}\\&\quad -{2\gamma }^{*}{\frac{{\partial }^{2}\psi }{\partial {y}^{2}}\left(\frac{{\partial }^{3}\psi }{\partial {y}^{3}}\right)}^{2}-{\gamma }^{*}{\frac{{\partial }^{4}\psi }{\partial {y}^{4}}\left(\frac{{\partial }^{2}\psi }{\partial {y}^{2}}\right)}^{2}+Re{S}^{2}E{R}_{m}-\frac{{p}^{*}}{\mu }\frac{{\partial }^{3}\psi }{\partial {y}^{3}}\\&\quad +{Gr}_{t}\theta +{Gr}_{c}\gamma -{Gr}_{p}\Phi -Da\frac{\partial \psi }{\partial y}.\end{aligned}$$

By Eqs. (31) and (40) we get

41 $$\begin{aligned}\frac{{\Omega }^{2}{a}^{2}\rho }{\mu }\frac{{\partial }^{2}\psi }{\partial {y}^{2}}&+\mu \frac{{\partial }^{4}\psi }{\partial {y}^{4}}-{\alpha }_{1}\frac{{\partial }^{5}\psi }{\partial {y}^{5}}+{\beta }_{1}\frac{{\partial }^{6}\psi }{\partial {y}^{6}}+12{\beta }^{*}\frac{{\partial }^{2}\psi }{\partial {y}^{2}}{\left(\frac{{\partial }^{3}\psi }{\partial {y}^{3}}\right)}^{2}+6{\beta }^{*}\frac{{\partial }^{4}\psi }{\partial {y}^{4}}{\left(\frac{{\partial }^{2}\psi }{\partial {y}^{2}}\right)}^{2}-{\gamma }_{1}\frac{{\partial }^{7}\psi }{\partial {y}^{7}}\\& -{2\gamma }^{*}{\left(\frac{{\partial }^{3}\psi }{\partial {y}^{3}}\right)}^{3}-6{\gamma }^{*}\frac{{\partial }^{2}\psi }{\partial {y}^{2}}\frac{{\partial }^{3}\psi }{\partial {y}^{3}}\frac{{\partial }^{4}\psi }{\partial {y}^{4}}-{\gamma }^{*}\frac{{\partial }^{2}\psi }{\partial {y}^{2}}\frac{{\partial }^{5}\psi }{\partial {y}^{5}}-\frac{{p}^{*}}{\mu }\frac{{\partial }^{4}\psi }{\partial {y}^{4}}+{Gr}_{t}\frac{\partial \theta }{\partial y}+{Gr}_{c}\frac{\partial \gamma }{\partial y}\\& -{Gr}_{p}\frac{\partial\Phi }{\partial y}-Da\frac{{\partial }^{2}\psi }{\partial {y}^{2}}=0.\end{aligned}$$

## Rate of volume flow and boundary conditions

The dimensional rate of fluid flow in the laboratory frame is

42 $$\xi =2\underset{0}{\overset{{\widetilde{h}}_{\widetilde{x}}}{\int }}\widetilde{U}\left(\widetilde{X},\widetilde{Y}\right)d\widetilde{Y}.$$

And in wave frame, the above equation is as follows

43 $$\zeta =2\underset{0}{\overset{{\widetilde{h}}_{\widetilde{x}}}{\int }}\widetilde{u}\left(\widetilde{x},\widetilde{y}\right)d\widetilde{y}.$$

By using Eqs. (20), (33), and (34) it can be seen that

44 $$\xi =\zeta +c\overline{h }.$$

The time-mean flow over a period $T$ at a fixed position $X$ is given as follows

45 $$\widetilde{\xi }=\frac{2}{\widetilde{T}}\underset{0}{\overset{{\widetilde{h}}_{\widetilde{x}}}{\int }}\xi dt.$$

After using Eq. (36) the above expression becomes as follows

46 $$\widetilde{\xi }=\zeta +ca.$$

The dimensionless time-mean flow $\vartheta$ and $F$ can be defined in the laboratory and wave frame respectively, as follows;

47 $$\vartheta =\frac{\widetilde{\xi }}{ac},F=\frac{\zeta }{ac}\Rightarrow \vartheta =F+1, \mathrm{and}\,F=2\underset{0}{\overset{{h}_{1}}{\int }}\frac{\partial \psi }{\partial y}dy.$$

## Numerical solution

The Runge–Kutta numerical method is one of the most important numerical analysis methods that are used in solving a system of ordinary differential equations, there are different formulas for the solution of the numerical method of Runge–Kutta method; for example, there is the Runge–Kutta method of the fourth order and also the Runge–Kutta method of the fifth order, and the most used method is the Runge–Kutta method of the fourth-order with shooting technique because it gives accurate results and is easy to use, its derivation depends on the Euler method, and the error rate in the solutions is very small and this is greatly required for the credibility of the results and also for their ease. Accordingly, the system of Eqs. (32)–(35) and (41) is a new system of nonlinear ordinary differential equations that describe the study of the fourth-grade nanofluid inside the flexible channel in addition to the boundary conditions (36) and (37). This system of equations has been solved numerically using the fourth-order Runge–Kutta method with the shooting technique using MTLAB program, this method depends on the basic step size used in the calculation is $\Delta y=0.001$ so that this value was reached after conducting many numerical experiments to reach the independence of the network and uses a variable step size to calculate the sharp changes in the variables in the areas dominated by viscosity inside the channel and where the limits the beginning and the end depend on the size of the basic step, so they are taken $-1.5\le y\le 1.5$ it has been obtained by substituting in ${h}_{2}$ and ${h}_{1}$ in the boundary conditions (36) and (37) by $\varepsilon =0.5$ and $x=1$. Our equations are dealt with in MATLAB because each nth-order equation is mutated into *n* of the first-order equations, then using the bvp4c function (in general, bvp4c is a finite difference code that implements the three-stage lobatto IIIa formula. This is a collocation formula and the collocation polynomial provides a C1-continuous solution that is fourth-order accurate uniformly. Mesh selection and error control are based on the residual of the continuous solution) to solve these first-order equations. The governing system of ordinary differential equations can be written in simplest form as follows;

48 $$\frac{{\partial }^{7}\psi }{\partial {y}^{7}}= \left(\begin{array}{c}\frac{{\Omega }^{2}{a}^{2}\rho }{\mu }\frac{{\partial }^{2}\psi }{\partial {y}^{2}}+\mu \frac{{\partial }^{4}\psi }{\partial {y}^{4}}-{\alpha }_{1}\frac{{\partial }^{5}\psi }{\partial {y}^{5}}+{\beta }_{1}\frac{{\partial }^{6}\psi }{\partial {y}^{6}}+12{\beta }_{2}\frac{{\partial }^{2}\psi }{\partial {y}^{2}}{\left(\frac{{\partial }^{3}\psi }{\partial {y}^{3}}\right)}^{2}\\ +6{\beta }_{2}\frac{{\partial }^{4}\psi }{\partial {y}^{4}}{\left(\frac{{\partial }^{2}\psi }{\partial {y}^{2}}\right)}^{2}-{2\gamma }^{*}{\left(\frac{{\partial }^{3}\psi }{\partial {y}^{3}}\right)}^{3}-6{\gamma }^{*}\frac{{\partial }^{2}\psi }{\partial {y}^{2}}\frac{{\partial }^{3}\psi }{\partial {y}^{3}}\frac{{\partial }^{4}\psi }{\partial {y}^{4}}-{\gamma }^{*}\frac{{\partial }^{2}\psi }{\partial {y}^{2}}\frac{{\partial }^{5}\psi }{\partial {y}^{5}}\\ -\frac{{P}^{*}}{\mu }\frac{{\partial }^{4}\psi }{\partial {y}^{4}}+{Gr}_{t}\frac{\partial \theta }{\partial y}+{Gr}_{c}\frac{\partial \gamma }{\partial y}-{Gr}_{p}\frac{\partial\Phi }{\partial y}-Da\frac{{\partial }^{2}\psi }{\partial {y}^{2}}\end{array}\right)/{\gamma }_{1}=0,$$

49 $$\frac{{\partial }^{2}\theta }{\partial {y}^{2}}=-\left(\begin{array}{c} Nb\left(\frac{\partial \theta }{\partial y}\right)\left(\frac{\partial\Phi }{\partial y}\right)+ Nt{\left(\frac{\partial \theta }{\partial y}\right)}^{2}+Bn M{\left(\frac{\partial \psi }{\partial y}\right)}^{2}\\ +Bn{\left(\frac{{\partial }^{2}\psi }{\partial {y}^{2}}\right)}^{2}+\beta \theta +3 R{\left({\theta }_{w}+\theta \right)}^{2}{\left(\frac{\partial \theta }{\partial y}\right)}^{2}\end{array}\right)/\left(\begin{array}{c}1-{ N}_{T\mathcal{F}} {N}_{\mathcal{F}T}\\ {+ R\left({\theta }_{w}+\theta \right)}^{3}\end{array}\right),$$

50 $$\frac{{\partial }^{2}\gamma }{\partial {y}^{2}}=-{N}_{\mathcal{F}T}\left(\frac{{\partial }^{2}\theta }{\partial {y}^{2}}\right),$$

51 $$\frac{{\partial }^{2}\Phi }{\partial {y}^{2}}=-\frac{Nt}{Nb}\left(\frac{{\partial }^{2}\theta }{\partial {y}^{2}}\right),$$

52 $$E=\frac{1}{{R}_{m}}\left(\frac{{\partial }^{2}\phi }{\partial {y}^{2}}\right).$$

The system of differential Eqs. (48)–(52) has been inserted into the MATLAB program as follows

$$\begin{aligned} &\mathcal{B}\left(1\right)=\mathcal{Y}\left(2\right),\; \mathcal{B}\left(2\right)=\mathcal{Y}\left(3\right),\; \mathcal{B}\left(3\right)=\mathcal{Y}\left(4\right),\; \mathcal{B}\left(4\right)=\mathcal{Y}\left(5\right),\; \mathcal{B}\left(5\right)=\mathcal{Y}\left(6\right),\; \mathcal{B}\left(6\right)=\mathcal{Y}\left(7\right),\\& \mathcal{B}\left(7\right)=\left(\begin{array}{c}{\Omega }^{2}{a}^{2}\rho Y\left(3\right)/\mu +\mu Y\left(5\right)-{\alpha }_{1} Y\left(6\right)+{\beta }_{1}Y\left(7\right)+12{\beta }_{2}Y\left(3\right){\left(\mathcal{Y}\left(4\right)\right)}^{2}\\ +6{\beta }_{2}Y\left(5\right){\left(\mathcal{Y}\left(3\right)\right)}^{2}-{2\gamma }^{*}{\left(\mathcal{Y}\left(4\right)\right)}^{3}-6{\gamma }^{*}Y\left(3\right)Y\left(4\right)Y\left(5\right)-{\gamma }^{*}Y\left(6\right)Y\left(3\right)\\ -{P}^{*}Y\left(5\right)/\mu +{Gr}_{t} Y\left(9\right)+{Gr}_{c} Y\left(11\right)-{Gr}_{p} Y\left(13\right)-Da Y\left(3\right)\end{array}\right)/{\gamma }_{1},\\ & \mathcal{B}\left(8\right)=\mathcal{Y}\left(9\right),\\ & \mathcal{B}\left(9\right)=-\left(\begin{array}{c}Nb Y\left(9\right)y\left(13\right)+Nt {\left(\mathcal{Y}\left(9\right)\right)}^{2}+Bn M {\left(\mathcal{Y}\left(2\right)\right)}^{2}\\ +Bn{\left(\mathcal{Y}\left(3\right)\right)}^{2}+ \beta Y\left(8\right)+3 R{ {\left(\mathcal{Y}\left(9\right)\right)}^{2}\left({\theta }_{w}+\mathcal{Y}\left(8\right)\right)}^{2}\end{array}\right)/\left(1-{N}_{T\mathcal{F}} {N}_{\mathcal{F}T}{+ R\left({\theta }_{w}+\mathcal{Y}\left(8\right)\right)}^{3}\right),\\ &\mathcal{B}\left(10\right)=\mathcal{Y}\left(11\right),\\ &\mathcal{B}\left(11\right)={-N}_{\mathcal{F}T}\mathcal{B}\left(9\right), \\ &\mathcal{B}\left(12\right)=\mathcal{Y}\left(13\right),\\ &\mathcal{B}\left(13\right)=-\frac{Nt}{Nb} F\left(9\right),\\ &\mathcal{B}\left(14\right)=\mathcal{Y}\left(15\right),\\ & \mathcal{B}\left(15\right)=E {R}_{m}.\end{aligned}$$

The boundary conditions can be written as follows

$$\begin{aligned} &{\mathcal{Y}}_{a}\left(1\right)=-\frac{F}{2},\quad {\mathcal{Y}}_{a}\left(2\right)=0,\quad {\mathcal{Y}}_{a}\left(8\right)=0,\quad {\mathcal{Y}}_{a}\left(10\right)=0,\quad {\mathcal{Y}}_{a}\left(12\right)=0,\quad {\mathcal{Y}}_{a}\left(14\right)=0,\quad at\, y=-1.5,\\ &{\mathcal{Y}}_{b}\left(1\right)=\frac{F}{2},\quad {\mathcal{Y}}_{b}\left(2\right)=0,\quad {\mathcal{Y}}_{b}\left(8\right)=1,\quad {\mathcal{Y}}_{b}\left(10\right)=1,\quad {\mathcal{Y}}_{b}\left(12\right)=1,\quad {\mathcal{Y}}_{b}\left(14\right)=1,\quad at\, y=1.5.\end{aligned}$$

Taking into consideration

$$\begin{aligned} &\psi = \mathcal{Y}\left(1\right),\quad \frac{\partial \psi }{\partial y}=\mathcal{Y}\left(2\right),\quad \frac{{\partial }^{2}\psi }{\partial {y}^{2}}=\mathcal{Y}\left(3\right),\quad \frac{{\partial }^{3}\psi }{\partial {y}^{3}}=\mathcal{Y}\left(4\right),\quad \frac{{\partial }^{4}\psi }{\partial {y}^{4}}=\mathcal{Y}\left(5\right),\quad \frac{{\partial }^{5}\psi }{\partial {y}^{5}}=\mathcal{Y}\left(6\right),\\ &\frac{{\partial }^{4}\psi }{\partial {y}^{4}}=\mathcal{Y}\left(5\right),\quad \frac{{\partial }^{5}\psi }{\partial {y}^{5}}=\mathcal{Y}\left(6\right),\quad \frac{{\partial }^{6}\psi }{\partial {y}^{6}}=\mathcal{Y}\left(7\right),\quad \frac{{\partial }^{7}\psi }{\partial {y}^{7}}=\mathcal{B}\left(7\right),\quad \theta = \mathcal{Y}\left(8\right),\quad \frac{\partial \theta }{\partial y}=\mathcal{Y}\left(9\right),\\ &\frac{{\partial }^{2}\theta }{\partial {y}^{2}}=\mathcal{B}\left(9\right),\quad \gamma = \mathcal{Y}\left(10\right),\quad \frac{\partial \gamma }{\partial y}=\mathcal{Y}\left(11\right),\quad \frac{{\partial }^{2}\gamma }{\partial {y}^{2}}=\mathcal{B}\left(11\right),\quad \Phi =\mathcal{Y}\left(12\right),\quad \frac{\partial\Phi }{\partial y}=\mathcal{Y}\left(13\right),\\ &\frac{{\partial }^{2}\Phi }{\partial {y}^{2}}=\mathcal{B}\left(13\right),\quad \phi = \mathcal{Y}\left(14\right),\quad \frac{\partial \phi }{\partial y}=\mathcal{Y}\left(15\right),\quad \frac{{\partial }^{2}\phi }{\partial {y}^{2}}=\mathcal{B}\left(15\right),\quad {\mathcal{Y}}_{a}\left(1\right)={\psi }_{y=-1.5}{\mathcal{Y}}_{b}\left(1\right)={\psi }_{y=1.5} \\ &{\mathcal{Y}}_{a}\left(2\right)={\left(\frac{\partial \psi }{\partial y}\right)}_{y=-1.5}{\mathcal{Y}}_{b}\left(2\right)={\left(\frac{\partial \psi }{\partial y}\right)}_{y=1.5}{\mathcal{Y}}_{a}\left(8\right)={\left(\theta \right)}_{y=-1.5}{\mathcal{Y}}_{b}\left(8\right)={\left(\theta \right)}_{y=1.5}{\mathcal{Y}}_{a}\left(10\right)={\left(\gamma \right)}_{y=-1.5}{\mathcal{Y}}_{b}\left(10\right)={\left(\gamma \right)}_{y=1.5} \\ &{\mathcal{Y}}_{a}\left(12\right)={\left(\Phi \right)}_{y=-1.5}{\mathcal{Y}}_{b}\left(12\right)={\left(\Phi \right)}_{y=1.5}{\mathcal{Y}}_{a}\left(14\right)={\left(\phi \right)}_{y=-1.5}{\mathcal{Y}}_{b}\left(14\right)={\left(\phi \right)}_{y=1.5}\end{aligned}$$

## Results and discussion

Table 1 represents a comparison between previous works that considered some parameters used in the present estimation to show the differences between the related topics. The current study resulted in the emergence of a set of physical parameters that you, dear reader, can identify when looking at the symbol table. The effect of each of the physical parameters resulting from the study on the distributions of axial velocity $u$, temperature $\theta$, solute concentration $\gamma$, nanoparticles volume fraction $\Phi$, pressure gradients $\frac{\partial P}{\partial x}$, magnetic force $\phi$, and finally induced magnetic field ${h}_{x}$ has been studied taking into account, the study of the behavior of the streamlines responsible for the occurrence of the trapping phenomenon under the influence of some of these parameters. It should be noted that when studying the effect of any parameter on any of the aforementioned distributions, the values of the rest of the other parameters remain constant at the following values $\Omega =0.5, a=1, E=0.6, {Gr}_{T}={Gr}_{p}={Gr}_{c}=0.5, {R}_{m}=1, {P}^{*}=\rho =\mu =1.6, S=0.3, Nb=Nt=M=Ec={\theta }_{w}=Re=R=Da=0.5, {N}_{T\mathcal{F}}={N}_{\mathcal{F}T}=0.4,{ \gamma }^{*}=0.003,{ \alpha }_{1}={\beta }_{1}={\beta }_{2}={\gamma }_{1}=0.4,\beta =-0.1, F=1$. In some detail, the results of this study will be discussed as follows.

**Table 1:** A comparison between some previous works.

	Nano-fluid	Rotation	Initial stress	Magnetic field	Thermal radiation	Heat source	Double diffusion	Viscous dissipation	Joule heating
Hayat et al.^31^	No	No	No	Yes	Yes	Yes	No	No	Yes
Akram and Razia^32^	Yes	No	No	No	No	No	Yes	No	No
Kothandapani et al.^33^	No	No	No	Yes	No	No	No	No	No
Haroun^34^	No	No	No	No	No	No	No	No	No
Akram et al.^35^	Yes	No	No	Yes	No	No	No	No	No
Hayat and Noreen^36^	No	No	No	No	No	Yes	No	No	No
Abd-Alla et al.^24^	No	Yes	Yes	Yes	No	No	No	No	No
Present study	Yes	Yes	Yes	Yes	Yes	Yes	Yes	Yes	Yes

### Axial velocity distribution

**Figure 2:**
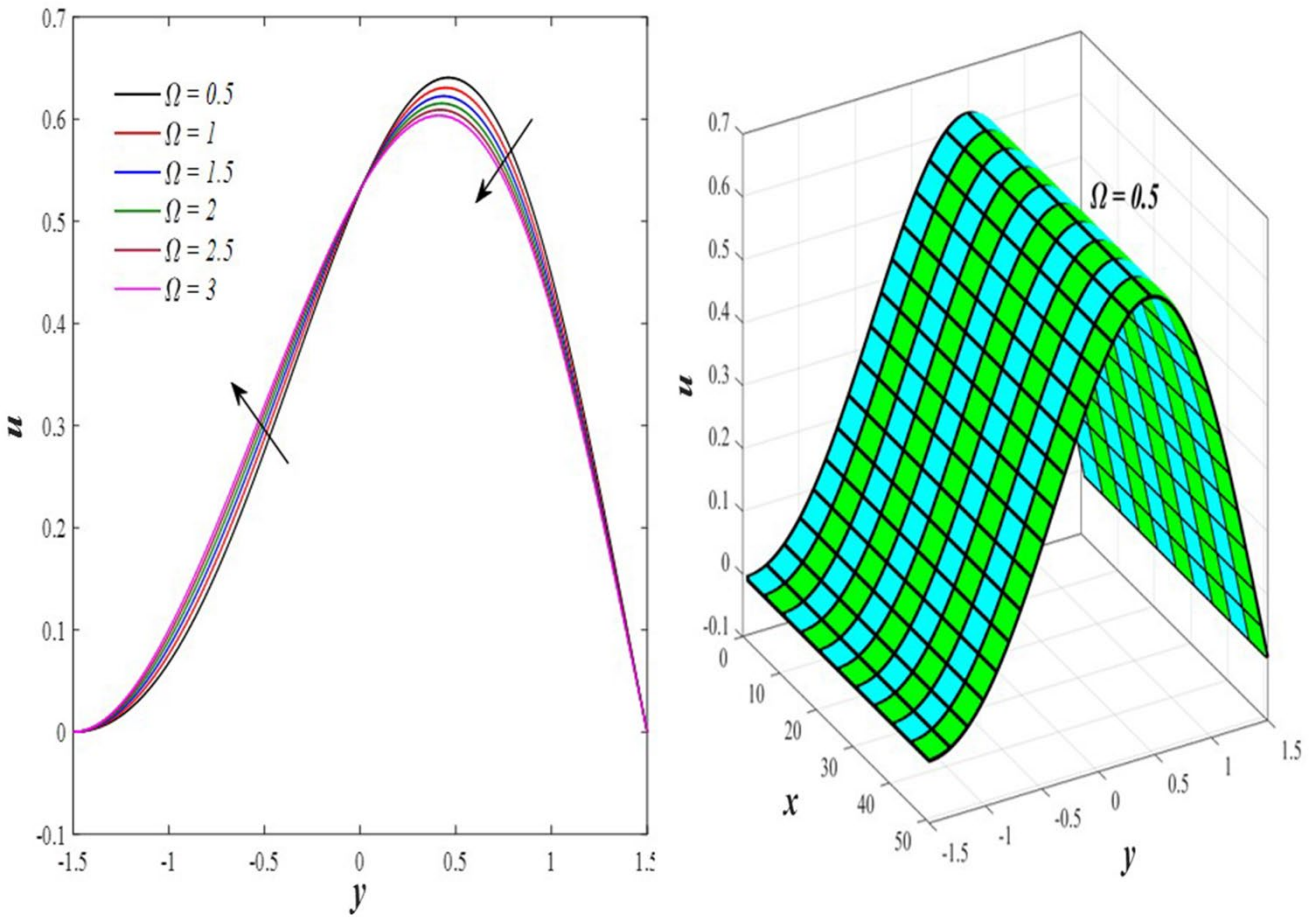
Effect of Ω on the axial velocity distribution *u* (2D and 3D).

**Figure 3:**
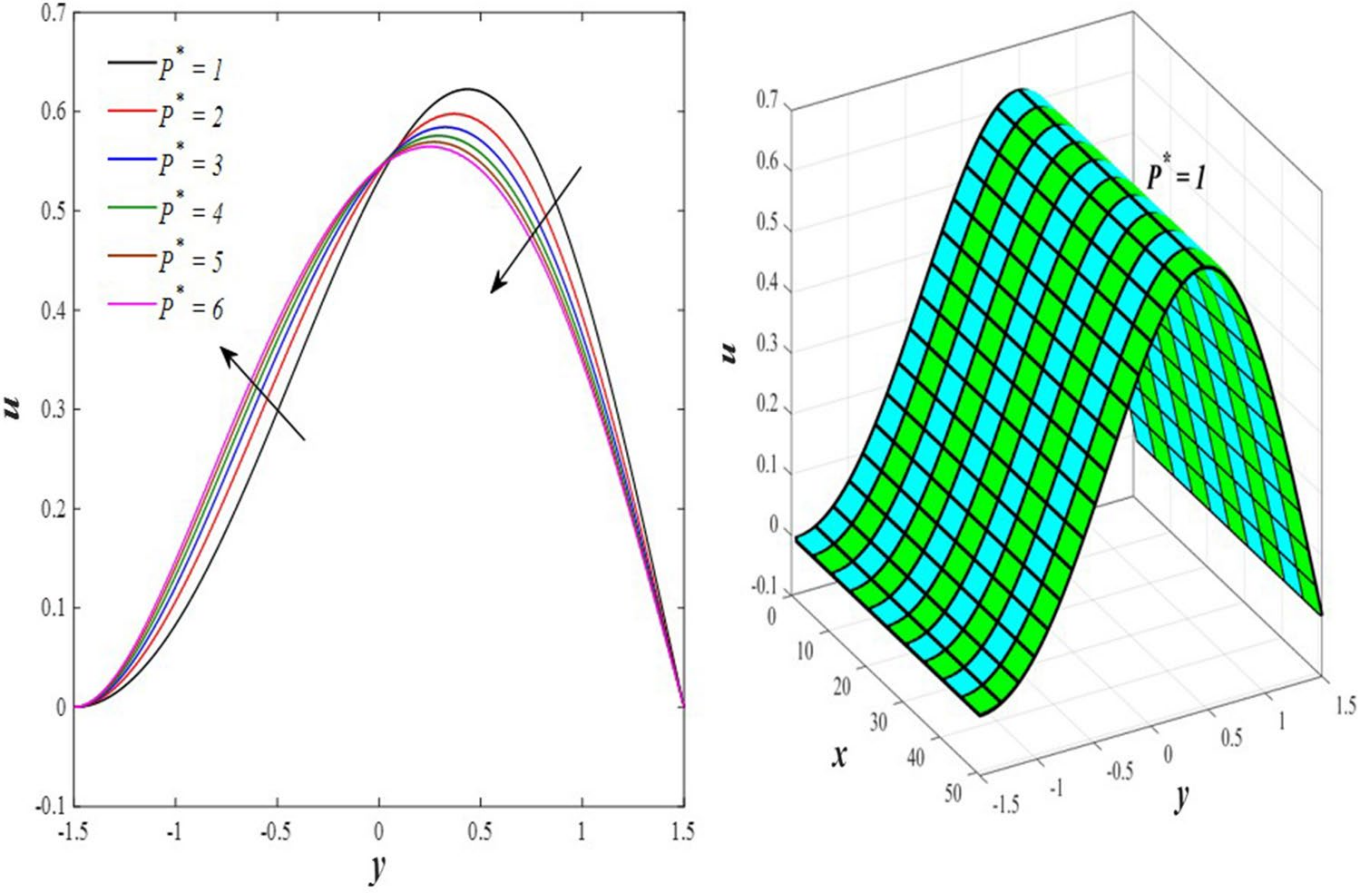
Effect of P^*^ on the axial velocity distribution *u* (2D and 3D).

**Figure 4:**
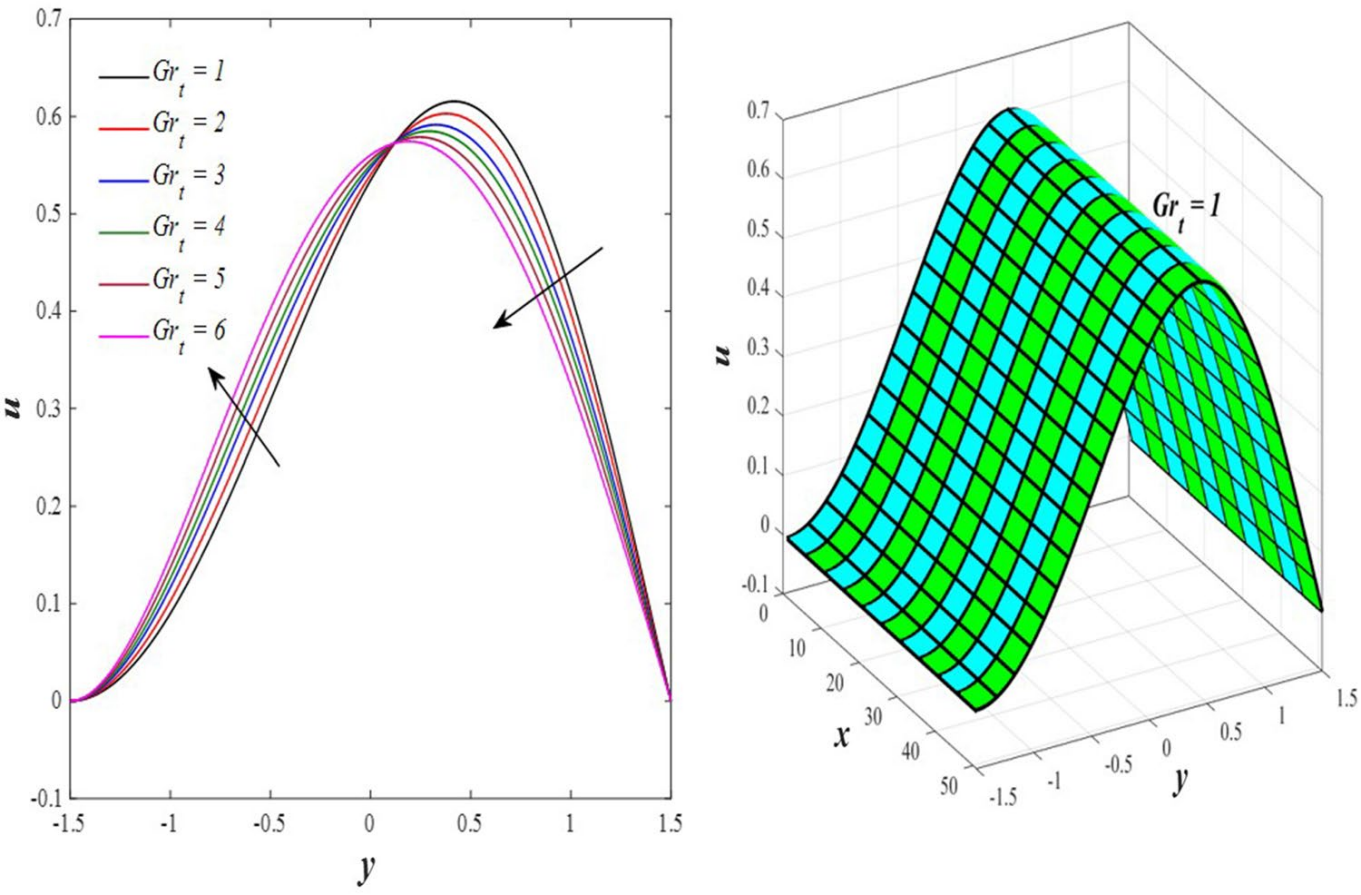
Effect of *Gr*_t_ on the axial velocity distribution *u* (2D and 3D).

**Figure 5:**
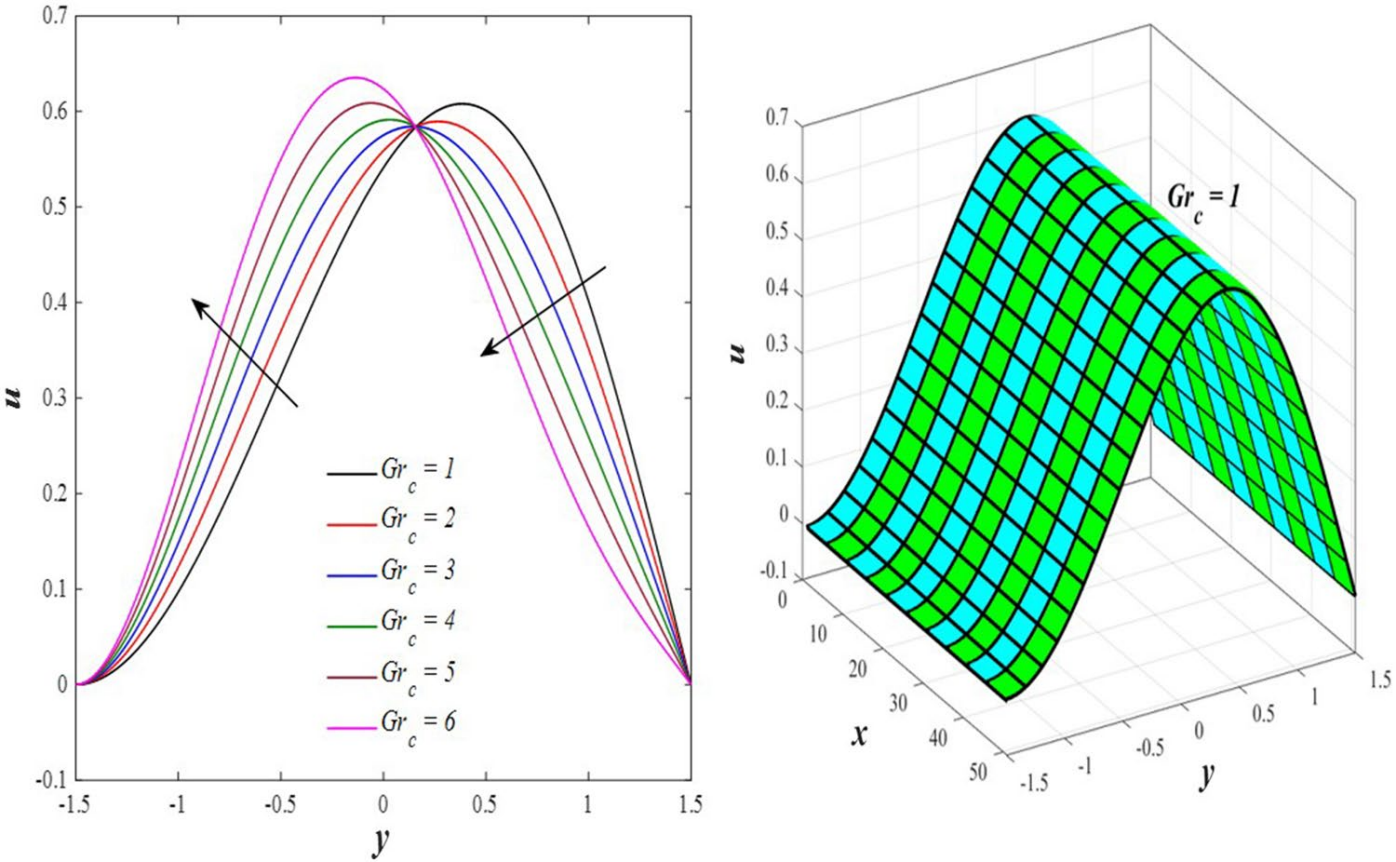
Effect of *Gr*_c_ on the axial velocity distribution *u* (2D and 3D).

**Figure 6:**
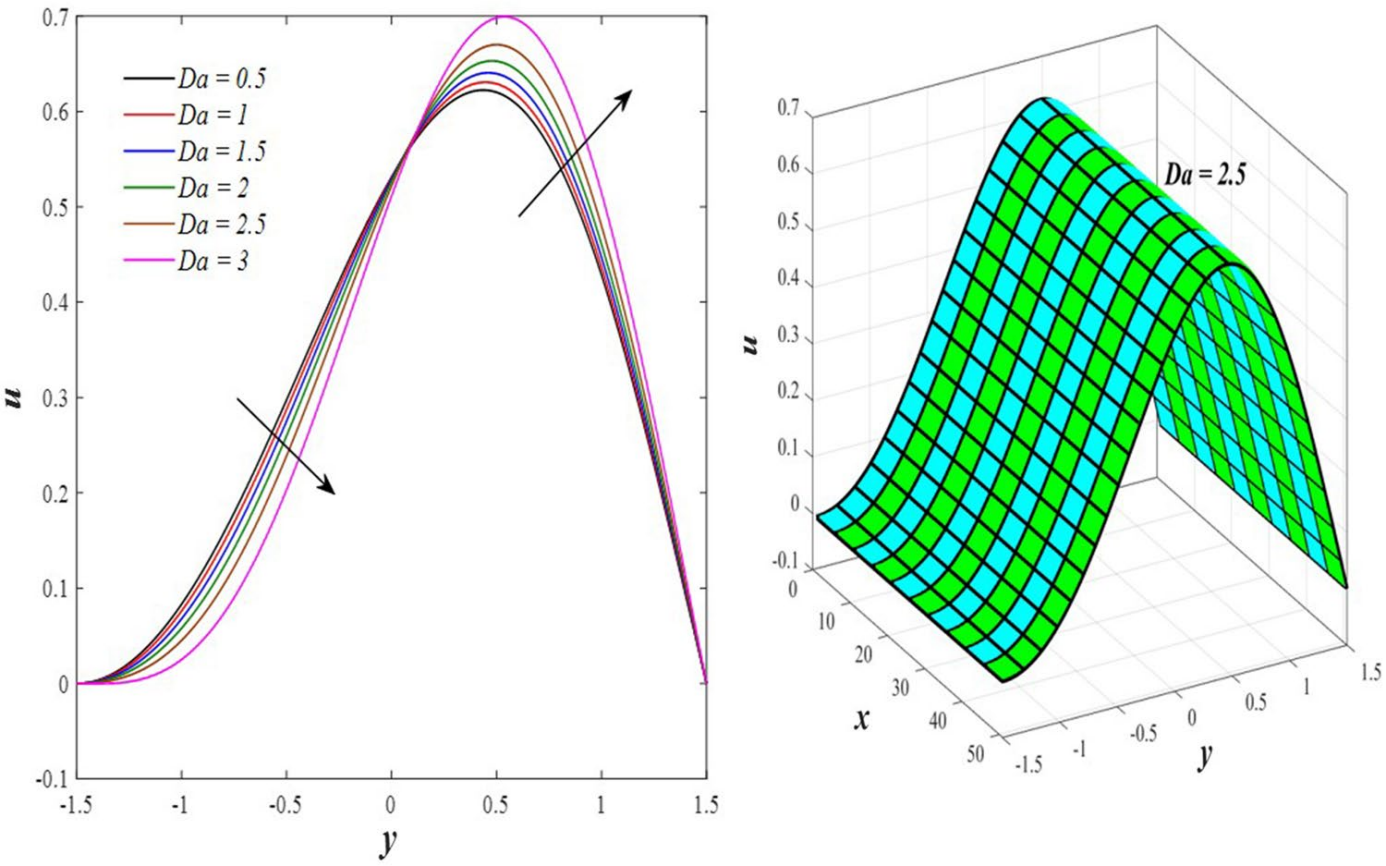
Effect of *Da* on the axial velocity distribution *u* (2D and 3D).

**Figure 7:**
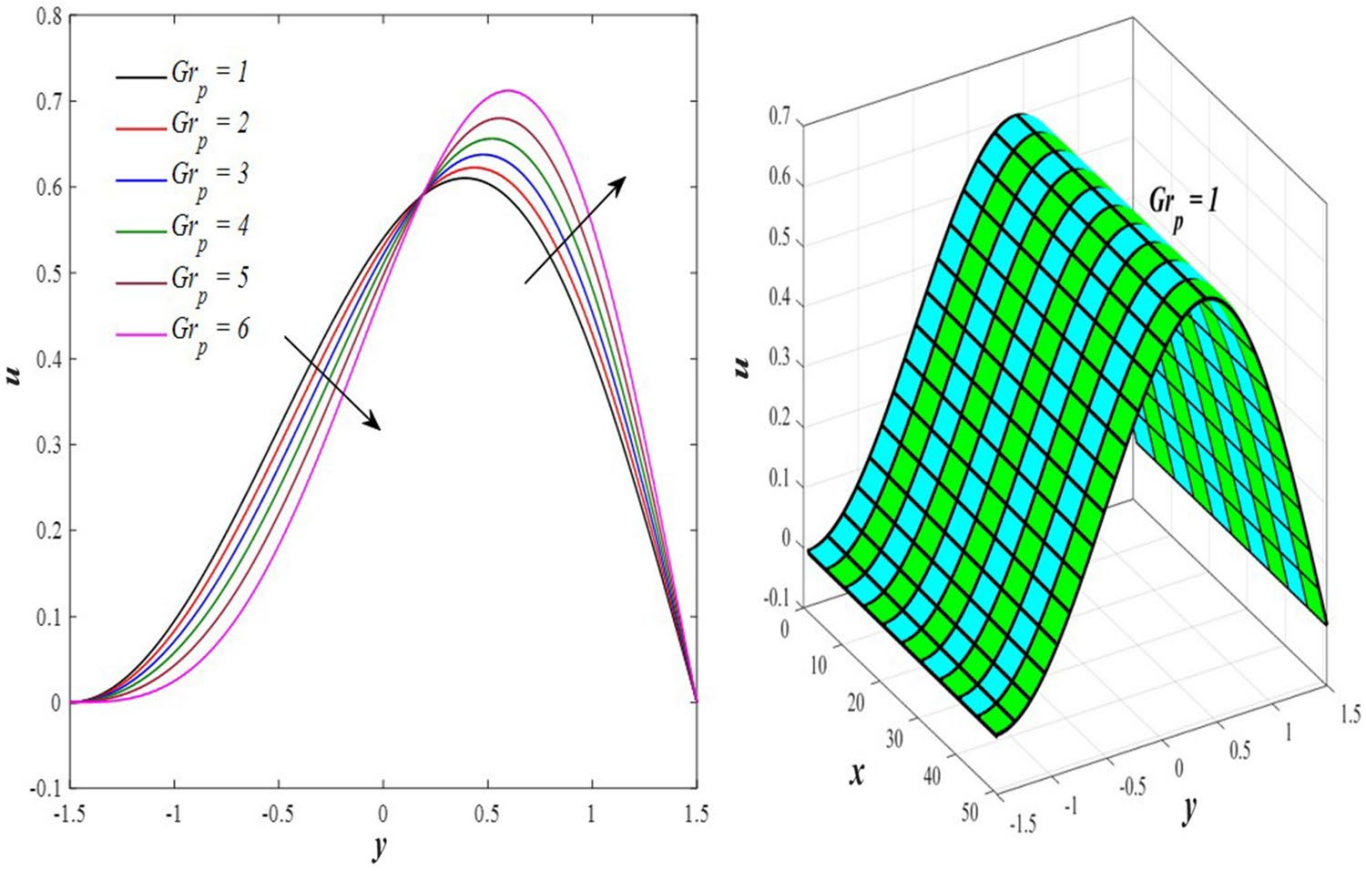
Effect of *Gr*_p_ on the axial velocity distribution *u* (2D and 3D).

**Figure 8:**
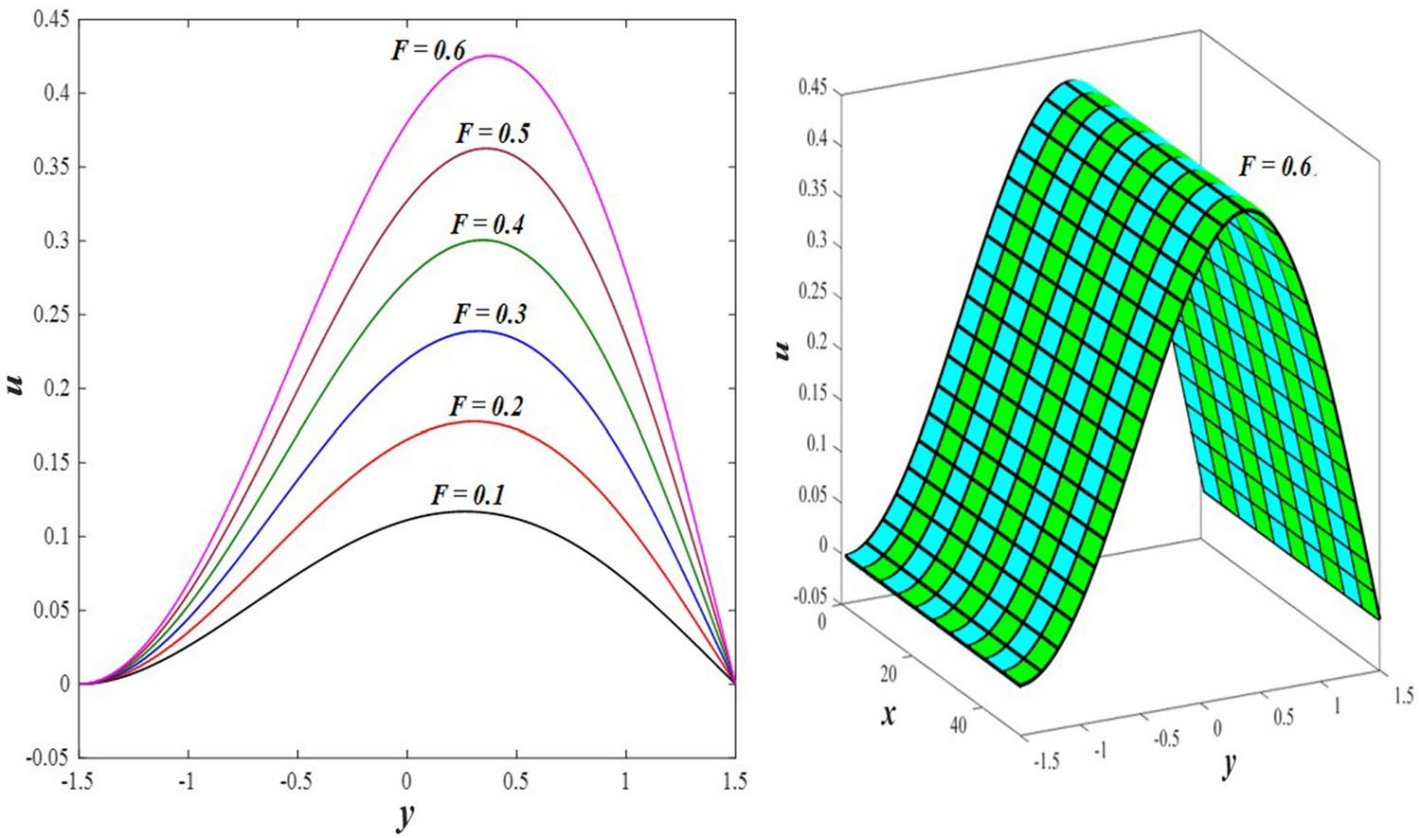
Effect of *F* on the axial velocity distribution *u* (2D and 3D).

The effects of the rotation $0.5\le \Omega \le 3$ and the initial stress $1\le {P}^{*}\le 6$ on the axial velocity distribution u are depicted in Figs. 2 and 3 (2D and 3D). It was discovered that their impact was positive in the distance $-1.5\le y\le 0.25$ and soon turned negative in the distance $0.25\le y\le 1.5$, as they caused the axial velocity distribution $u$ to exhibit irregular behavior. The fact that the direction of rotation $\Omega$ is perpendicular to the axis of the flexible channel and the fluid is subjected to rotation around the axis of the tube when applied is the reason why rotation $\Omega$ causes an irregular behavior in the axial fluid velocity on a physical level. A flow is rotational if its vortices vector is not zero in all of its regions while the fluid elements are rotating about their axis as it flows along streamlines. Also, the fluid that is moving inside the channel responds strongly to initial stress ${P}^{*}$, causing turbulence between the fluid layers and making the fluid's velocity and movement irregular. In contrast, Figs. 4 and 5 (2D and 3D) can be used to explain the effects of heat Grashof number $1\le {Gr}_{t}\le 6$ and solutal Grashof number $1\le {Gr}_{c}\le 6$ on the axial velocity distribution $u$. You will notice that both numbers cause an enhancement in the axial velocity distribution in the interval $-1.5\le y\le 0.25$, while a reduction of this distribution occurs in the interval $0.25\le y\le 1.5$. This indicates that both numbers also produce irregular behavior in the axial velocity distribution $u$. Physically, it is important to note that the ratio between the fluid's thermal buoyancy force and its viscous hydrodynamic force is not fixed. This means that an increase in the fluid's thermal buoyancy force directly causes a decrease in the fluid's viscosity, making it easier for the fluid to move, and the other way around. According to what was said, the heat Grashof number ${Gr}_{t}$ describes the relative relationship between the fluid's thermal buoyancy force and its viscous hydrodynamic force. Therefore, an increase in the fluid velocity distribution is accompanied by a decrease in the fluid viscosity rate at a distance of $-1.5\le y\le 0.25$ from the channel, while the opposite is true at a distance of $0.25\le y\le 1.5$ from the channel. The behavior of the solutal Grashof number ${Gr}_{c}$ is identical to that of the heat Grashof number ${Gr}_{t}$. Plots of the changes in the axial velocity distribution u caused by Darcy number $0.5\le Da\le 3$ and nanoparticles Grashof number ${1\le Gr}_{p}\le 6$ are shown in Figs. 6 and 7 (2D and 3D). It was found that both numbers caused a decrease in the axial velocity distribution $u$ in the range $-1.5\le y\le 0.25$ of the channel, but that behavior quickly changed to an increase in the same distribution in the range $0.25\le y\le 1.5$ of the channel. Physically, it is well known that a porous medium is a resistive medium that is permeated by many voids or pores that the fluid permeates during its flow. Its presence inside the flexible channel acts as a force that slows down the movement of the fluid. The thickness of this medium is inversely proportional to the fluid velocity; the larger the thickness, the smaller the fluid velocity in the range $-1.5\le y\le 0.25$, and the smaller the thickness, the greater the fluid velocity in the range $0.25\le y\le 1.5$. On the other hand, the relationship between the fluid's viscosity and the thermal buoyancy force exerted by the nanoparticles and its impact on the fluid's axial velocity distribution $u$ is significantly influenced by the nanoparticle Grashof number ${Gr}_{p}$. As a result, the axial velocity distribution of viscosity rise against the buoyancy force of nanoparticles shows a slight decrease in the range $-1.5\le y\le 0.25$, while the opposite occurs in the range $0.25\le y\le 1.5$. Lastly, the axial velocity distribution u experiences a regular, positive behavior as a result of the influence of the mean flow coefficient $0.1\le F\le 0.6$. To put it another way, the axial velocity distribution u experiences regular increases as a result of the influence of the mean flow coefficient $0.1\le F\le 0.6$. This is due to the strong positive relationship that exists between this coefficient and this distribution. As shown in Fig. 8 (2D and 3D), this coefficient is crucial to the consolidation and strengthening of the axial fluid velocity within the channel.

### Temperature, solutal concentration, and nanoparticles volume fraction distributions

**Figure 9:**
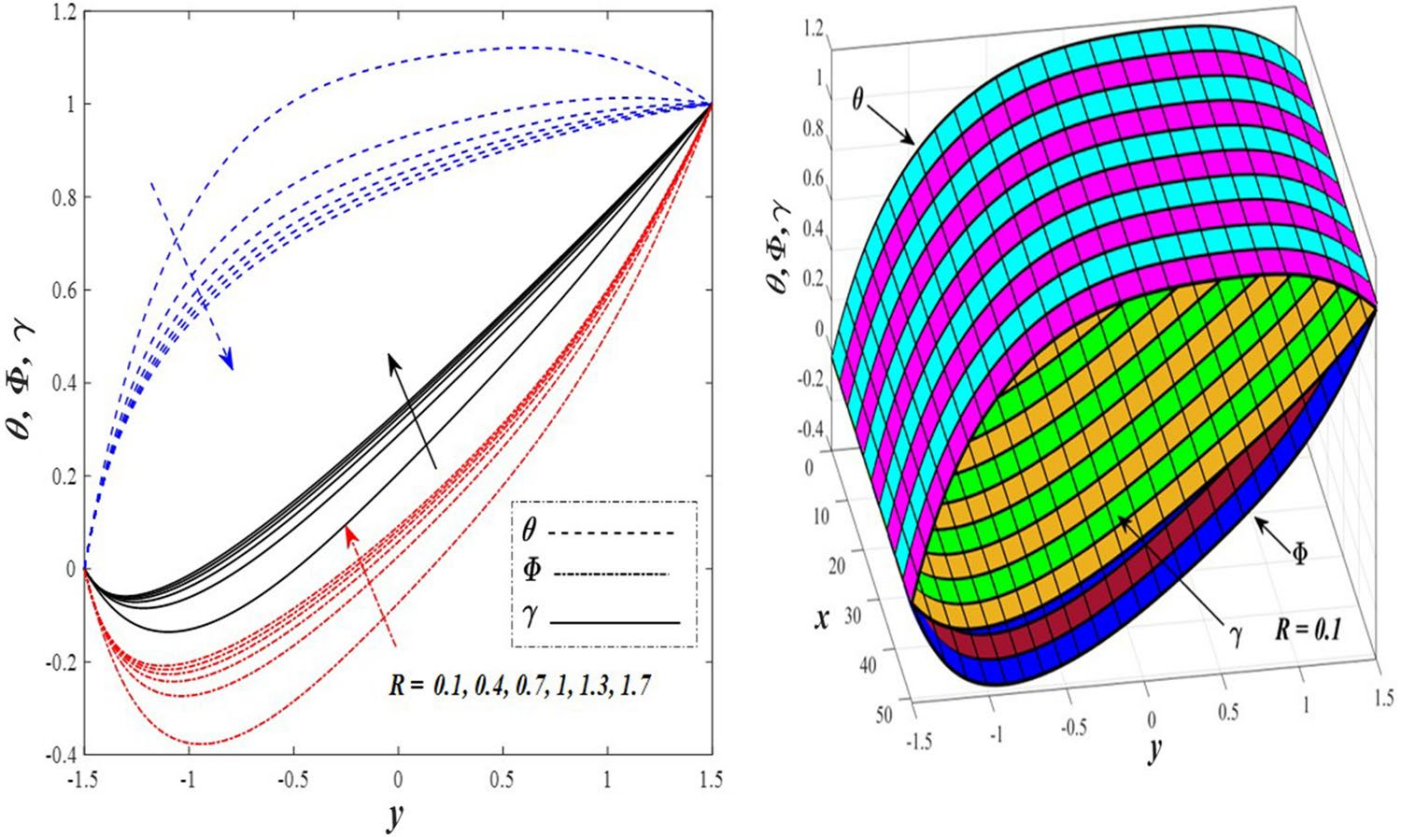
Effect of *R* on the distributions of temperature, solutal concentration, and nanoparticles volume fraction (2D and 3D).

**Figure 10:**
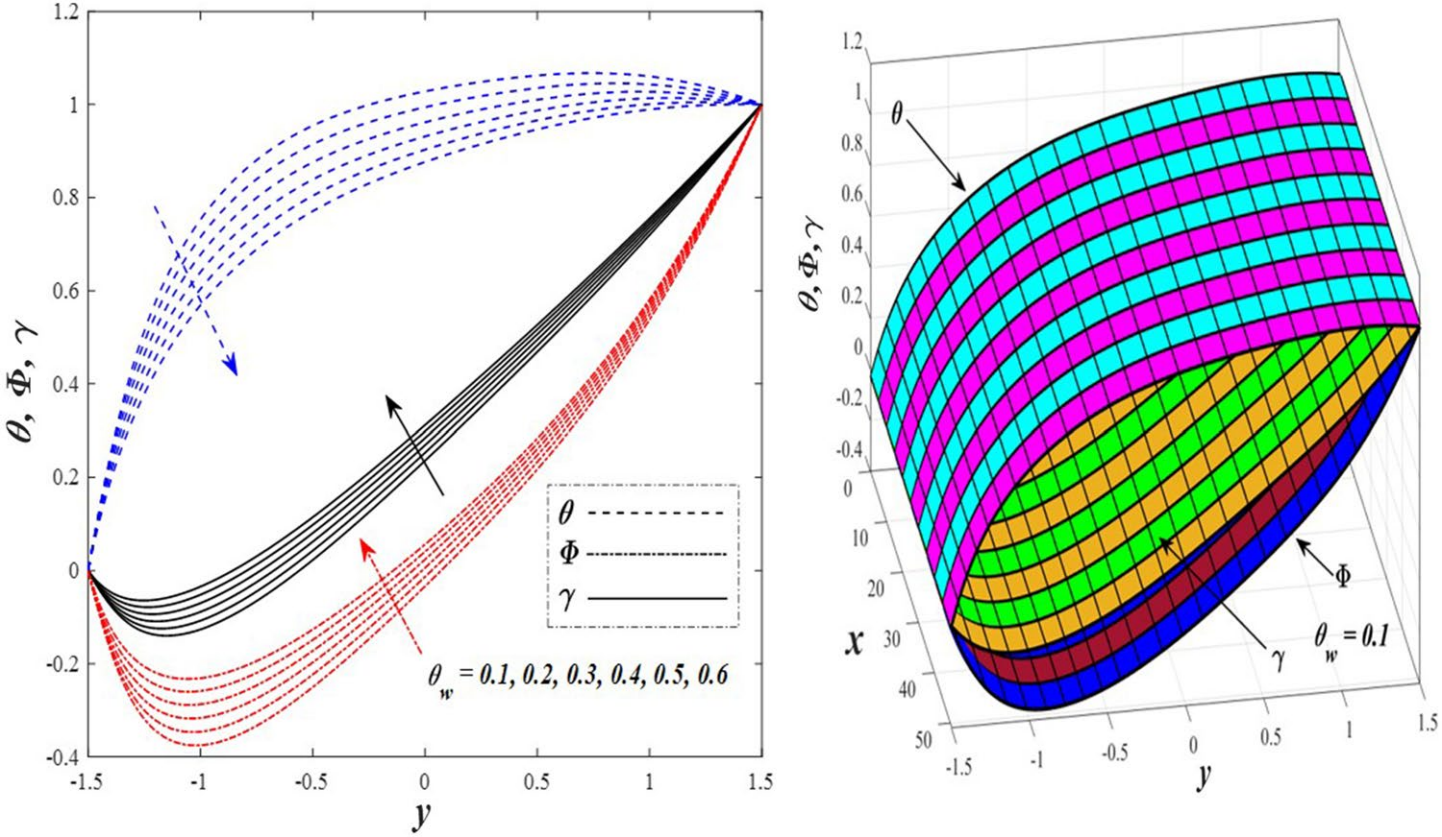
Effect of *θ*_*w*_ on the distributions of temperature, solutal concentration, and nanoparticles volume fraction (2D and 3D).

**Figure 11:**
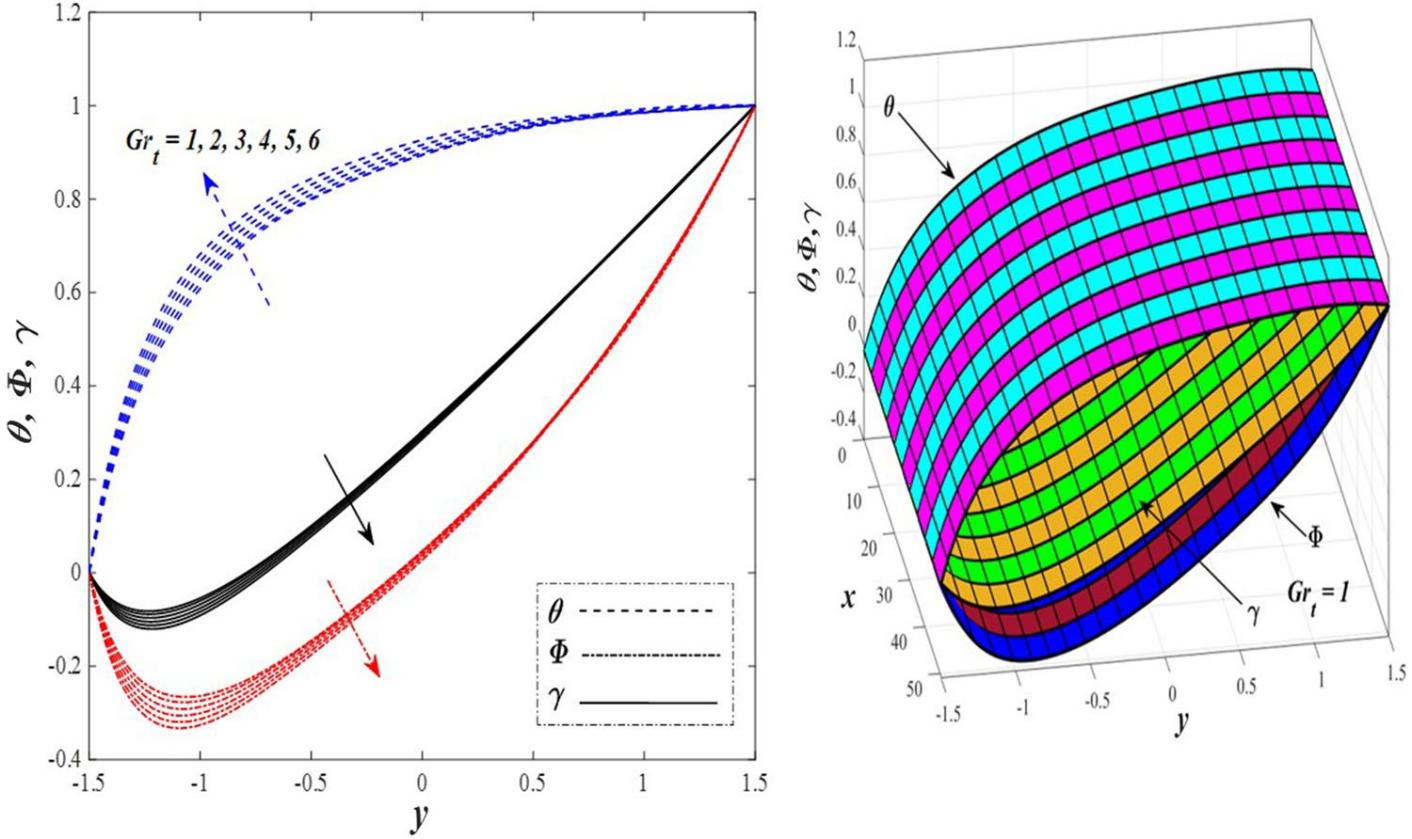
Effect of *Gr*_t_ on the distributions of temperature, solutal concentration, and nanoparticles volume fraction (2D and 3D).

**Figure 12:**
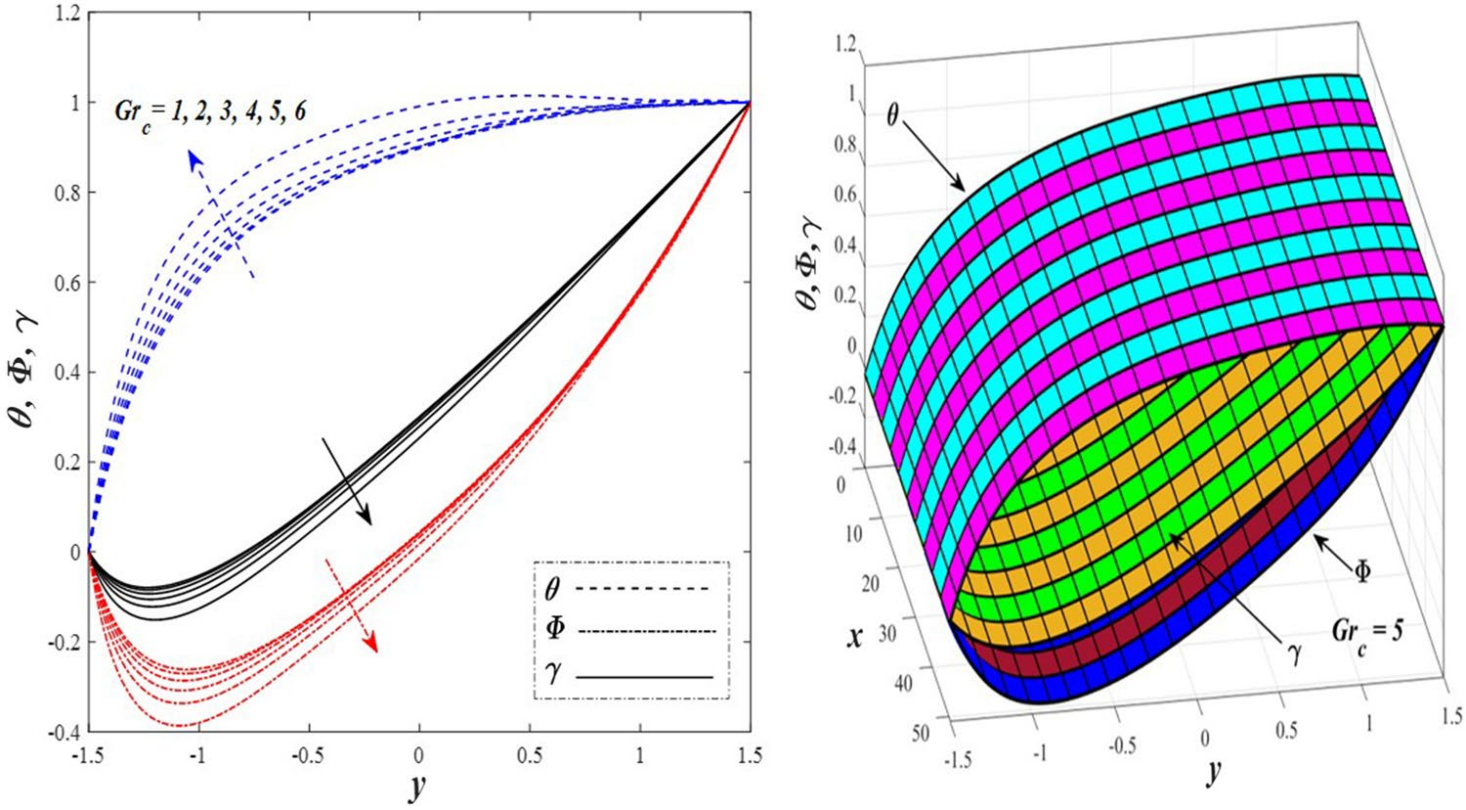
Effect of *Gr*_c_ on the distributions of temperature, solutal concentration, and nanoparticles volume fraction (2D and 3D).

**Figure 13:**
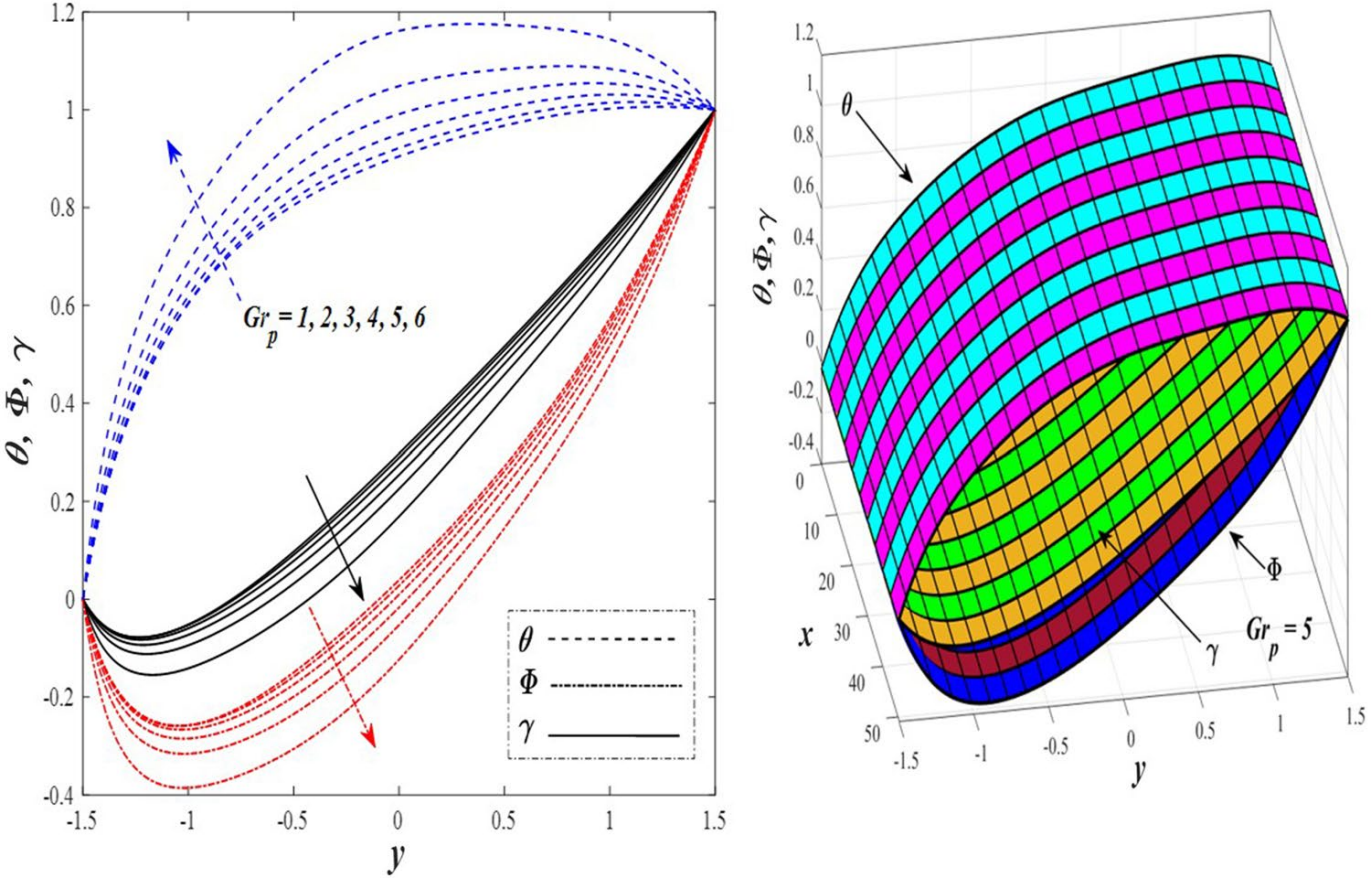
Effect of *G*r_p_ on the distributions of temperature, solutal concentration, and nanoparticles volume fraction (2D and 3D).

**Figure 14:**
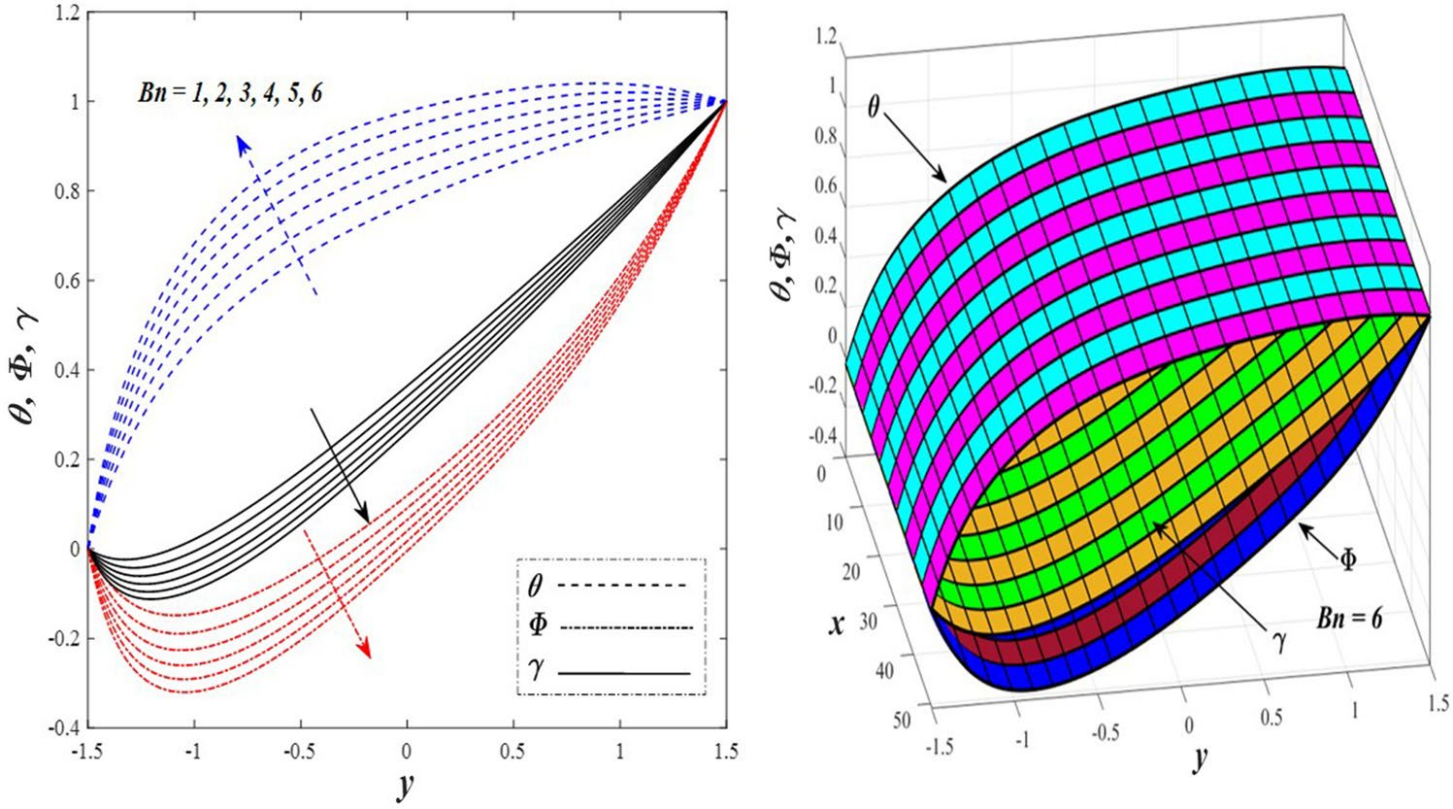
Effect of *Bn* on the distributions of temperature, solutal concentration, and nanoparticles volume fraction (2D and 3D).

**Figure 15:**
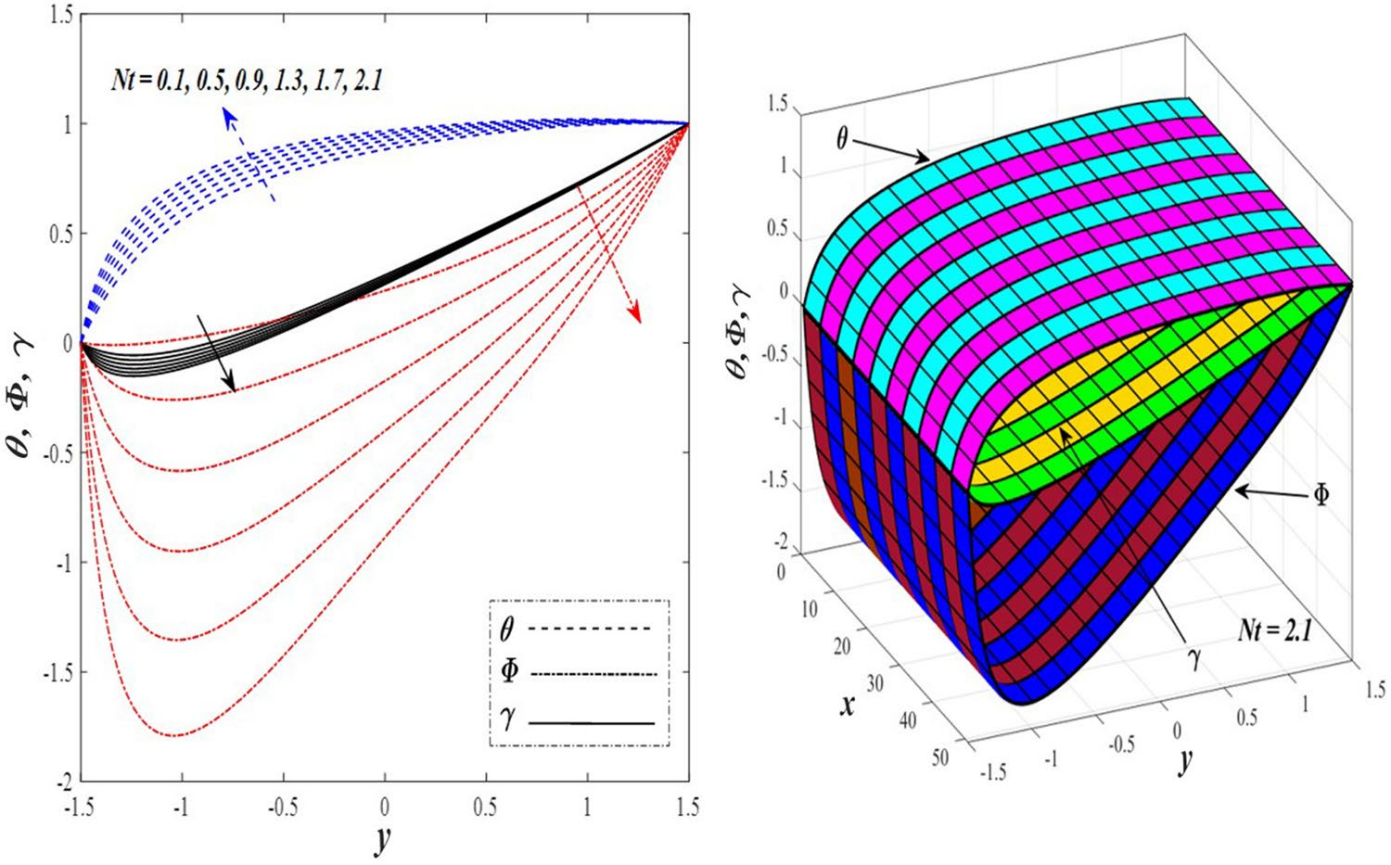
Effect of *Nt* on the distributions of temperature, solutal concentration, and nanoparticles volume fraction (2D and 3D).

**Figure 16:**
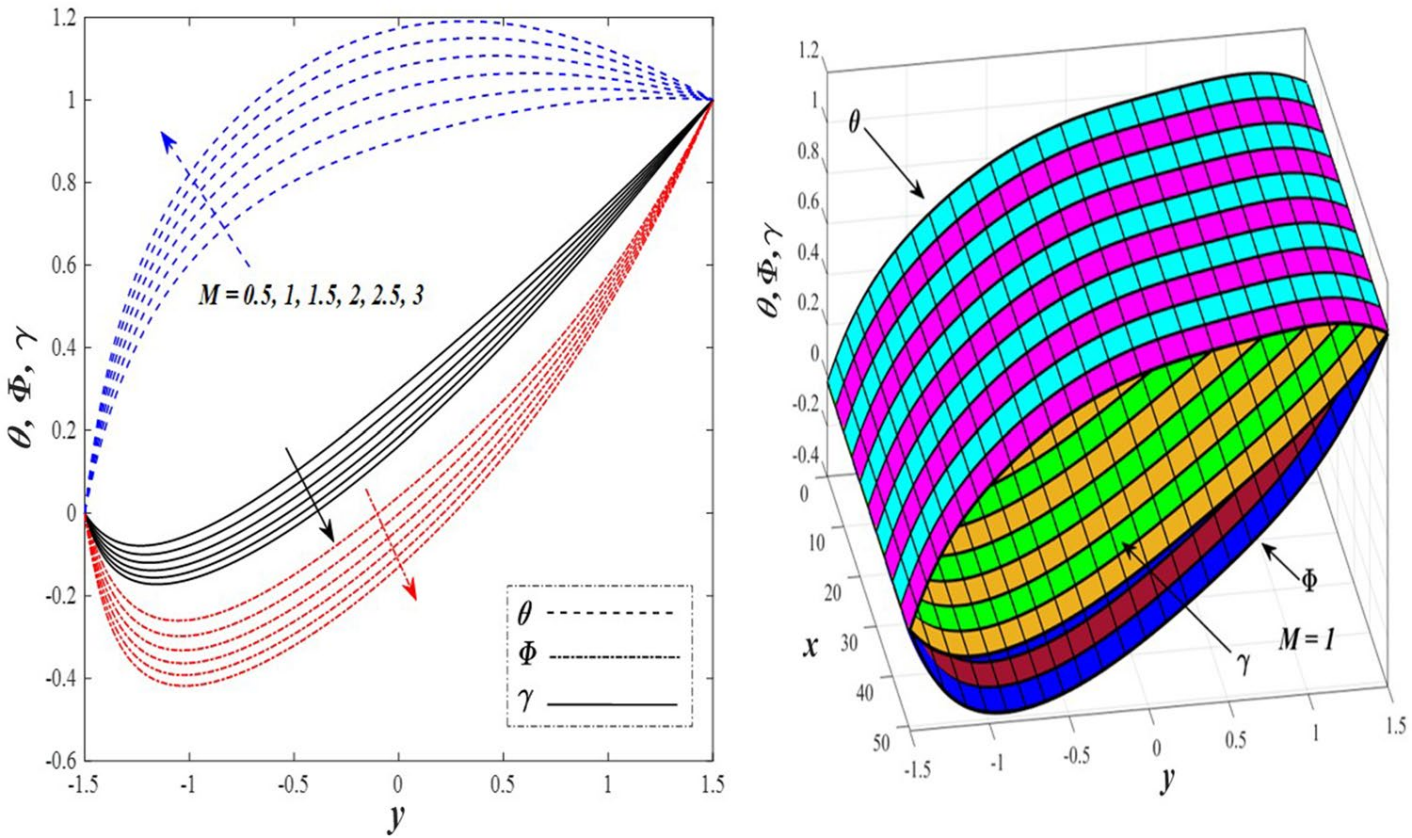
Effect of *M* on the distributions of temperature, solutal concentration, and nanoparticles volume fraction (2D and 3D).

**Figure 17:**
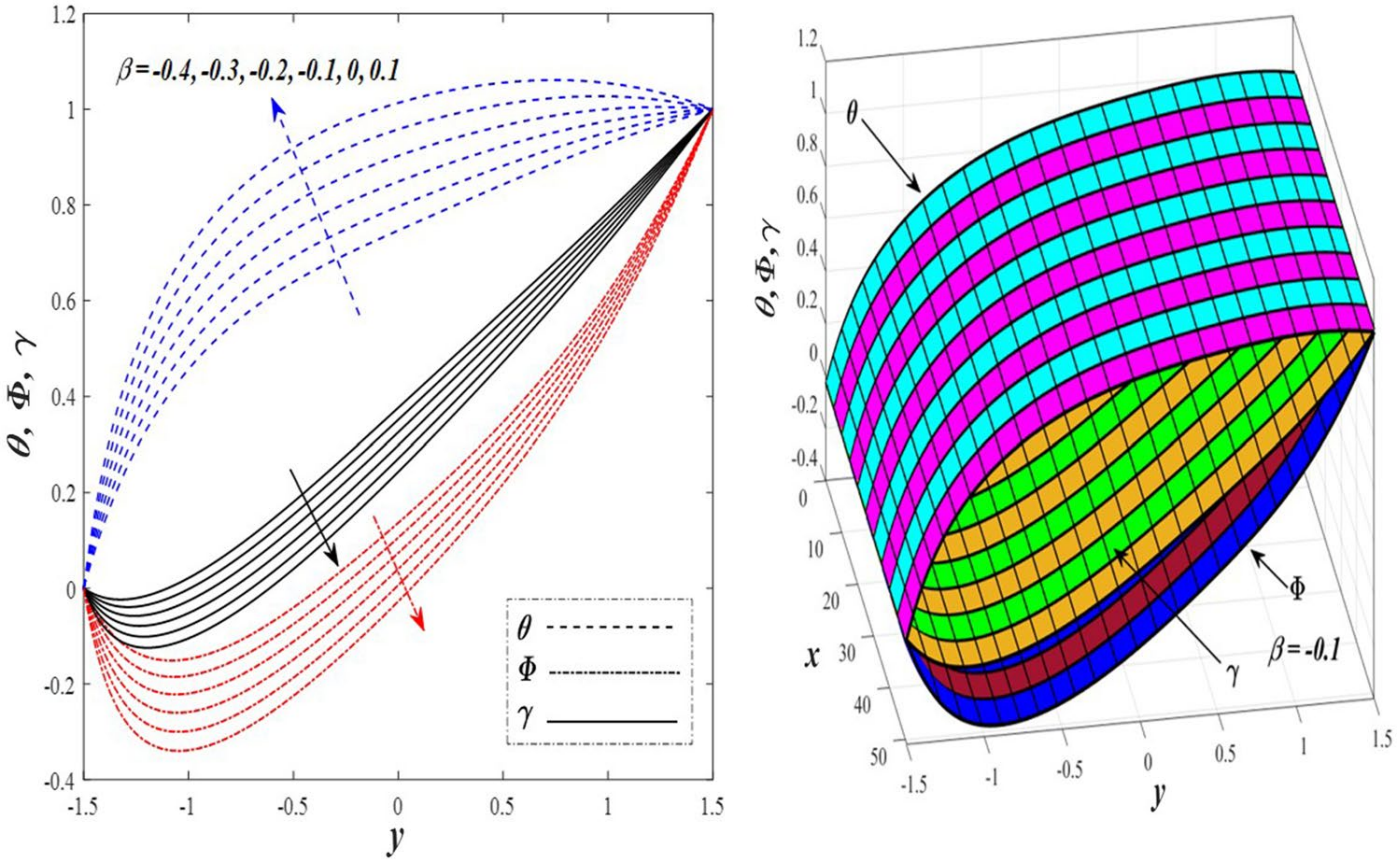
Effect of *β* on the distributions of temperature, solutal concentration, and nanoparticles volume fraction (2D and 3D).

**Figure 18:**
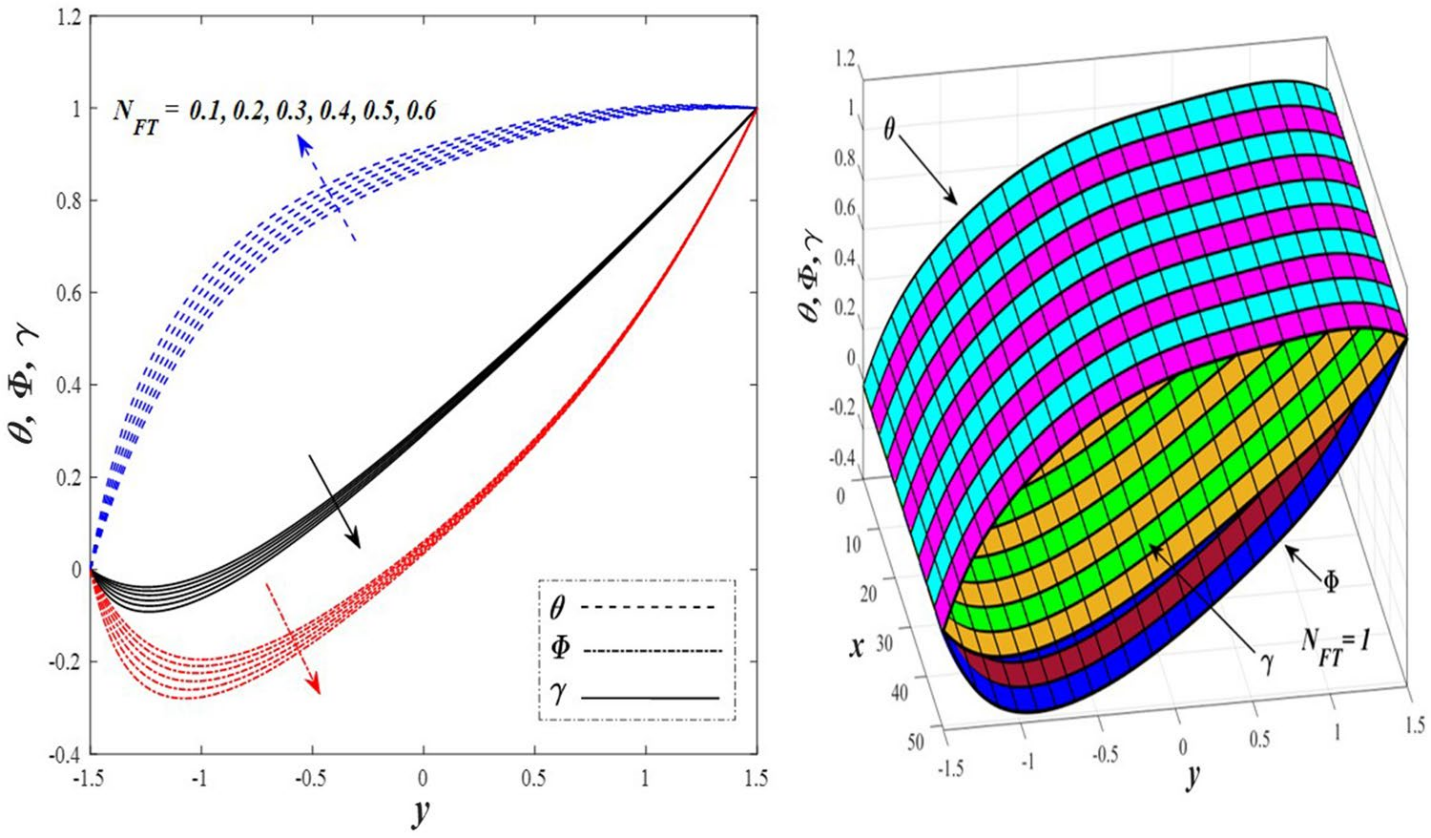
Effect of *N*_*FT*_ on the distributions of temperature, solutal concentration, and nanoparticles volume fraction (2D and 3D).

**Figure 19:**
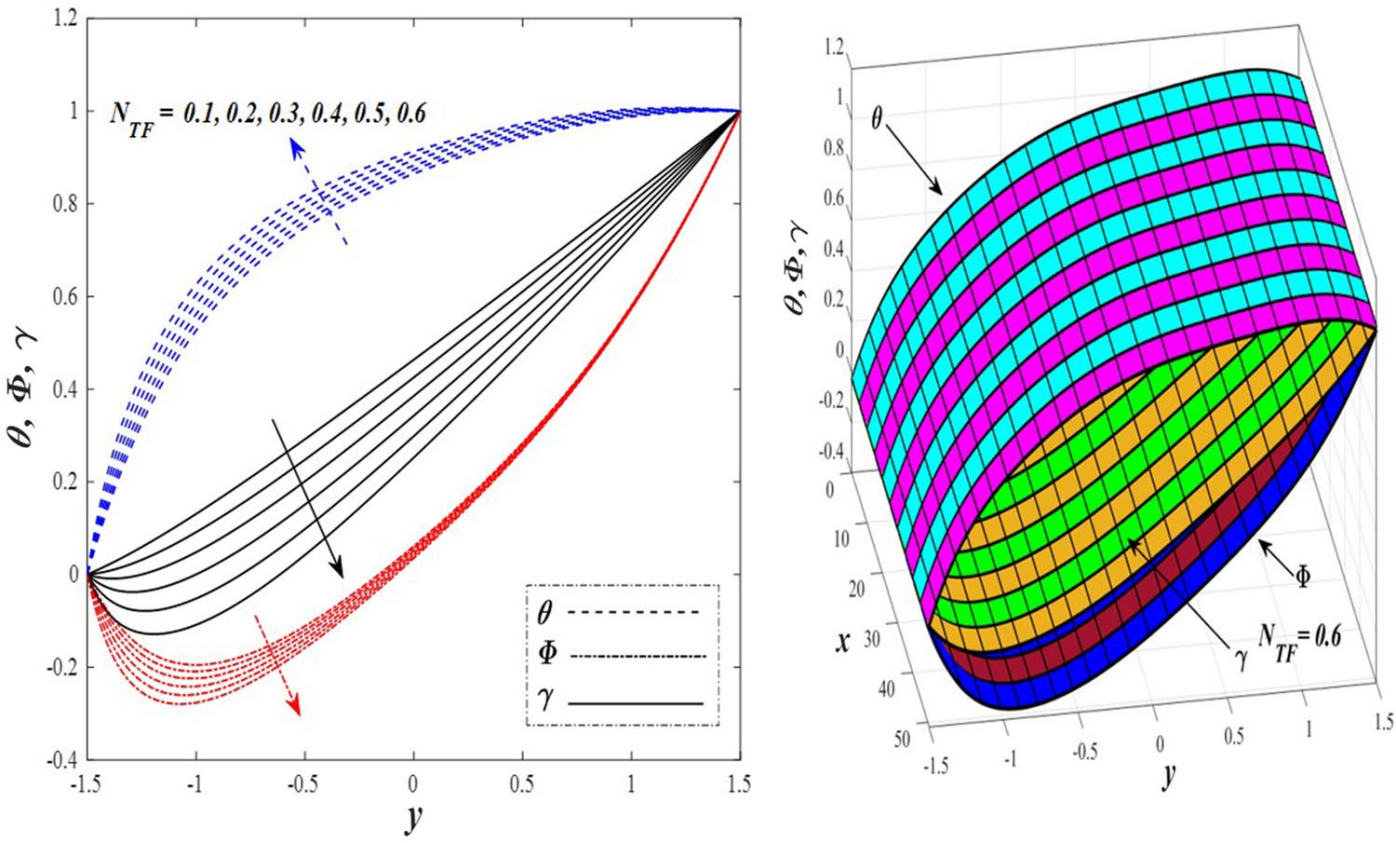
Effect of *N*_*TF*_ on the distributions of temperature, solutal concentration, and nanoparticles volume fraction (2D and 3D).

**Figure 20:**
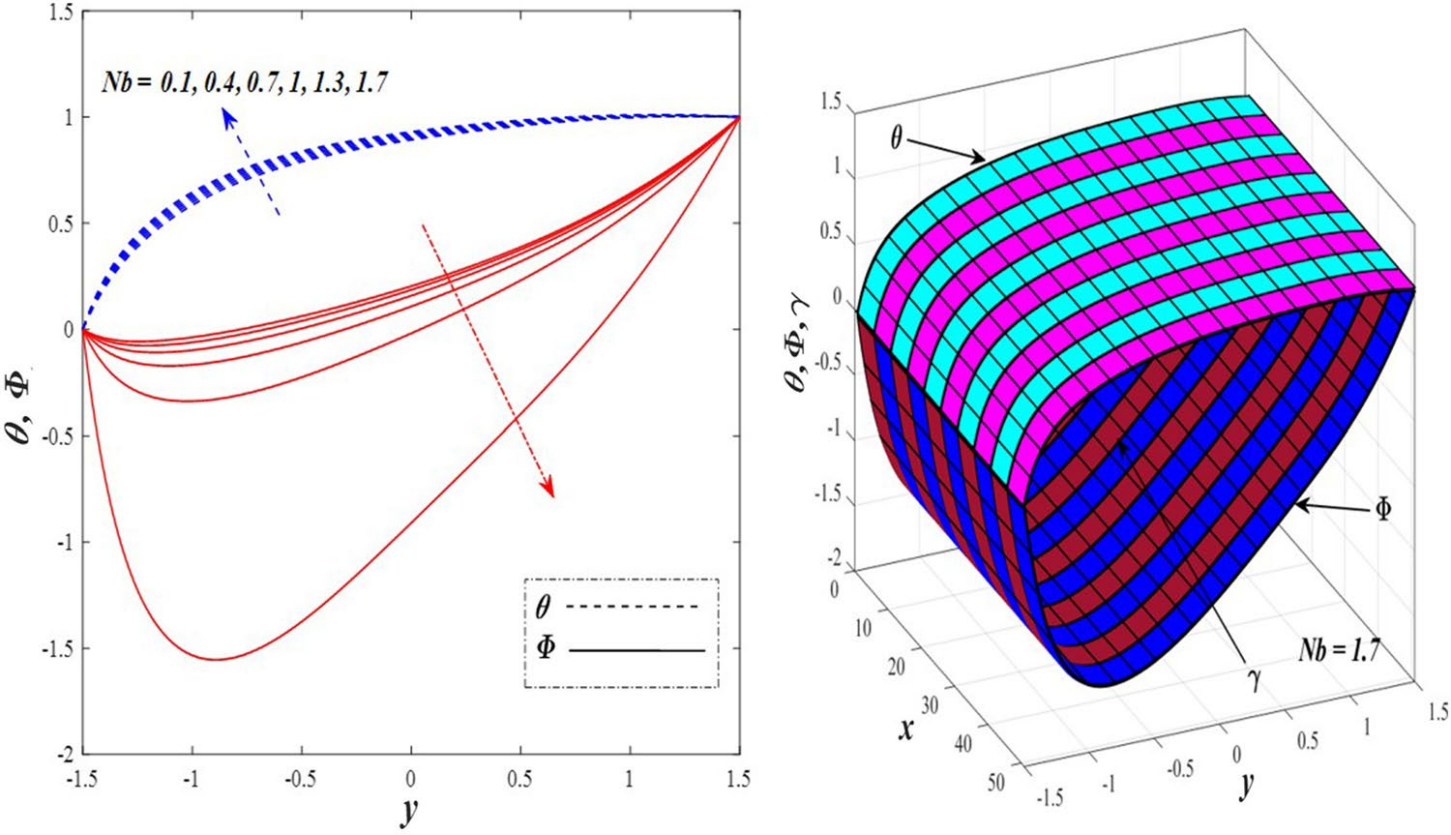
Effect of *Nb* on the distributions of temperature and nanoparticles volume fraction (2D and 3D).

Take a look at Figs. 9 and 10 (2D and 3D) to learn how the non-linear thermal radiation parameter $0.1\le R\le 1.7$ and the temperature ratio parameter $0.1\le {\theta }_{w}\le 0.6$ affect the distributions of temperature, solutal concentration, and nanoparticles volume fraction. It was found that increasing the values of the previous two parameters caused a noticeable negative change in the distribution of temperature and an increase in the distributions of both solutal concentration and nanoparticles volume fraction. Physically, the inverse relationship between the thermal conductivity coefficient and these two parameters, on the one hand, and the occurrence of thermal diffusion far from the system, on the other, explains why there was a significant decrease in the temperature distribution under the influence of the various increasing values taken by the non-linear thermal radiation coefficient and also the temperature ratio coefficient it was assumed that there would be an enhancement in the temperature distribution under the influence of the various increasing values taken by these two parameters. This also caused an increase in the interfacial distances between the basic fluid particles, with a concentration of nanoparticles higher inside the channel and a concentration of nanoparticles lower near the channel walls. This also explains the support in the distribution of the solvent concentration and the volume fraction of the nanoparticles. Figures 11 and 12 (2D and 3D) depict the effects of heat Grashof number $1\le {Gr}_{t}\le 6$ and solutal Grashof number $1\le {Gr}_{c}\le 6$ on the temperature $\theta$, solutal concentration $\gamma$, and nanoparticles volume fraction $\Phi$ distributions, respectively. It was found that these two numbers had a positive effect on the temperature distribution, while they had a negative effect on the distributions of both solutal concentration and nanoparticles volume fraction. Physically, The relative relationship between the thermal buoyancy force of the liquid and the viscous hydrodynamic force of the liquid is determined by the heat Grashof number $1\le {Gr}_{t}\le 6$ , and accordingly, the enhancement in the temperature distribution resulting from the influence of the different increasing values of the thermal Grashof number was mainly caused by the dominance and control of the thermal buoyancy force over the hydrodynamic viscous force for the fluid, this means a higher temperature in exchange for lower viscosity, on the one hand. On the other hand, this also causes an increase in the movement of the basic fluid molecules, and thus a decrease in the interstitial distances between those molecules, and this means a decrease in the solutal concentration distribution, in addition to a decrease in the concentration of nanoparticles inside the fluid, and this is also a decrease in the nanoparticles volume fraction. It should be noted that the solutal Grashof number $1\le {Gr}_{c}\le 6$ follows the same approach as the heat Grashof number $1\le {Gr}_{t}\le 6$. To concentrate on the impacts of the nanoparticle's Grashof number ${1\le Gr}_{p}\le 6$ and the Brinkman number $1\le Bn\le 6$ on the way of behaving of the distributions of temperature $\theta$, solutal focus $\gamma$, and nanoparticle volume division Φ, dear reader, you ought to take a gander at Figs. 13 and 14 (2D and 3D), you can see that these two numbers concur in their positive effect on the temperature distribution $\theta$ while they concur in their adverse consequence on the solutal concentration distribution $\gamma$, and nanoparticles volume fraction distribution $\Phi$. Physically, the Grashof number of the nanoparticle,${1\le Gr}_{p}\le 6$ has a significant impact on the behavior of the nanoparticle and its relationship to the temperature distribution. As a result, the positive effect of the temperature distribution under the influence of this number is to increase the thermal buoyancy of the nanoparticles within the fluid at the expense of the fluid viscosity. Additionally, this has a negative impact on the distribution of the solvent concentration because of the shorter distances between the underlying fluid particles. The activity of the thermal conductivity coefficient, which in turn works on the transfer of temperature from the channel walls to inside the fluid because the Brinkman number $1\le Bn\le 6$ expresses the heat transfer process from the wall inside the system and this also causes an increase in the movement of the basic fluid molecules and nanoparticles and a decrease in the interfacial distances between them, is the primary reason for the enhancement of the temperature distribution in contrast to the decrease in the other two distributions under the influence of the various increasing values of the Brinkman number. Increasing the various values of the thermophoresis parameter $0.1\le Nt \le 2.1$ supports and enhances the temperature distribution $\theta$ in exchange for reducing the distributions of the solutal concentration $\gamma$ and the nanoparticle volume fraction $\Phi$ this behavior is depicted in Fig. 15 (2D and 3D). Physically, thermophoresis is a thermal phenomenon that occurs in a specific medium with a temperature difference. It causes matter particles to move from one region of the medium with a higher temperature to one with a lower temperature until the temperature is the same in all of the medium's regions. This phenomenon is controlled by a thermophoresis coefficient $0.1\le Nt \le 2.1$. It is strengthening and increasing values have led to an increase in the fluid temperature as a result of the movement of fluid particles at a high temperature, the explanation for the reduction in the distributions of the solutal and the nanoparticles volume fraction is the scattering and precariousness that happens to the essential liquid atoms and nanoparticles inside the liquid when the upsides of the thermophoresis coefficient are improved. The enhancement in the values of the magnetic field $0.5\le M\le 3$ is straightforwardly trailed by an upgrade in the temperature distribution θ as well as, a diminishing in the distributions of solutal concentration γ, and nanoparticles volume fraction Φ, this is obvious to you, dear peruser in Fig. 16 (2D and 3D). Physically, the presence of a drag force that prevents the development of momentum and results in a decrease in the flow inside the channel causes friction between the layers of the viscous fluid and the walls of the channel, resulting in an increase in the fluid's temperature, this is the primary reason for the improvement in the temperature distribution under the influence of the magnetic field parameter $0.5\le M\le 3$. Likewise, the reaction of nanoparticles and natural liquid particles inside the liquid to the strength of the attractive field made their fixations decline. It turned out, dear reader, that the effect is positive on the temperature distribution $\theta$ and negative on the other two distributions $\gamma ,\Phi$ when the heat generation/absorption parameter $-0.4\le \beta \le 0.1$ was plotted in Fig. 17 (2D and 3D) specifically to explain its effect on the three main distributions temperature $\theta$, solutal concentration $\gamma$, and nanoparticles volume fraction $\Phi$. Physically, the expansion in the field of temperature dissemination is communicated from heat absorption $(\beta <0)$ to heat generation $(\beta >0)$ by the normal active energy of the liquid atoms coming about because of their retention of thermal power, which prompts an expansion in the development and speed of the fluid particles inside in light of the fact that the temperature decides the movement energy related with the development of liquid particles and nanoparticles which likewise causes little distances between liquid particles. The effects of Soret parameter $0.1\le {N}_{\mathcal{F}T}\le 0.6$ and Dufour parameter $0.1\le {N}_{T\mathcal{F}}\le 0.6$ on the three main distributions of temperature, solutal concentration, and nanoparticles volume fraction are depicted in Figs. 18 and 19. An increase in the values of the previous two parameters resulted in an increase in the temperature distribution $\theta$, which in turn led to an improvement in thermal diffusion and thermal conductivity, as well as a decrease in the distribution of solutal concentration $\gamma$ and the nanoparticle volume fraction $\Phi$. Finally, in Fig. 20 the temperature distribution is shown to have a positive direct relationship with the Brownian motion coefficient $0.1\le Nb\le 1.7$, while the distribution of the nanoparticle's volume fraction is shown to have an opposite relationship with the same coefficient. Physically, the improvement of the Brownian motion coefficient $0.1\le Nb\le 1.7$ prompted the development in the distribution of temperature $\theta$ and a decrease in the nanoparticle's volume fraction distribution $\Phi$ because of the arbitrary development of the liquid and nanoparticles together, which is constrained by the Brownian motion coefficient it is by and large answerable for the development of irregular nanoparticles inside the fluid.

### Pressure gradient distribution

**Figure 21:**
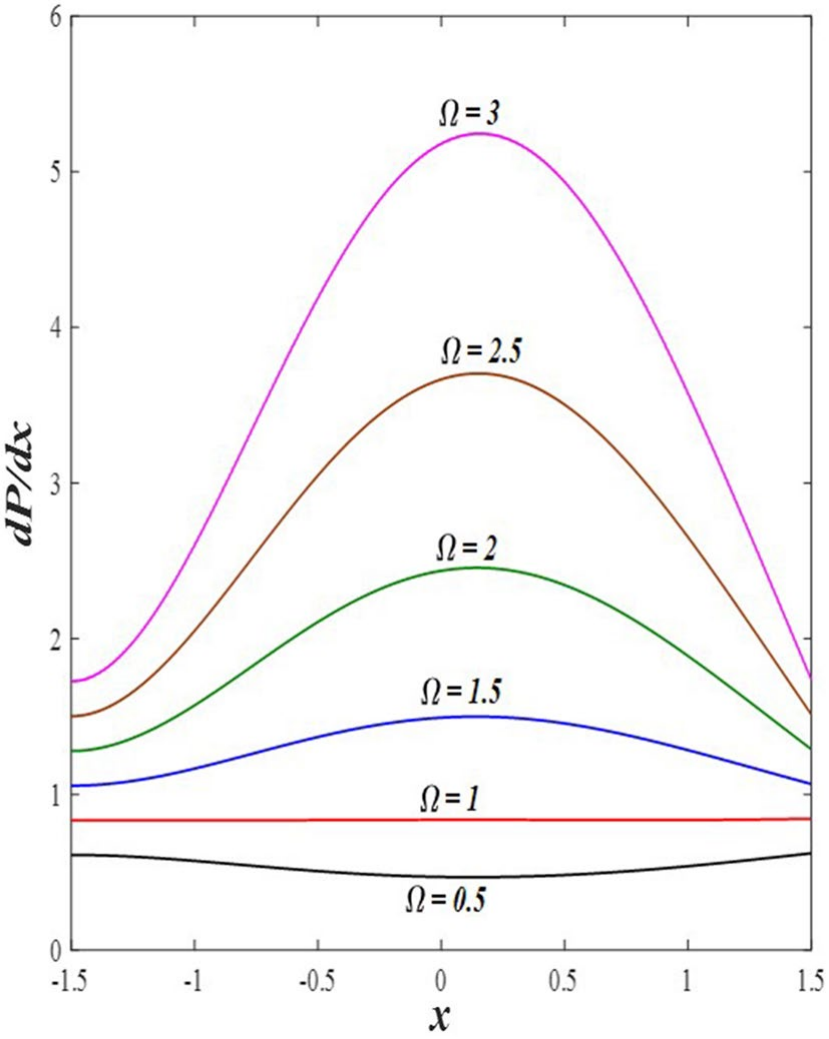
Pressure gradient distribution for different values of Ω.

**Figure 22:**
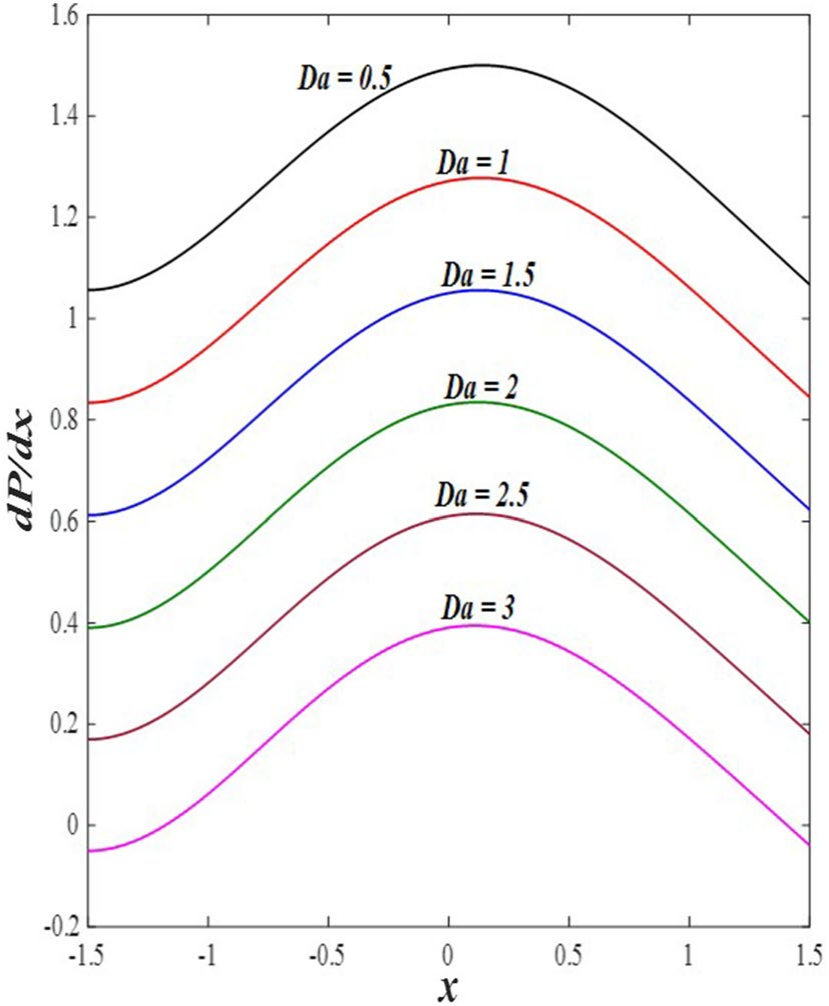
Pressure gradient distribution for different values of *Da*.

**Figure 23:**
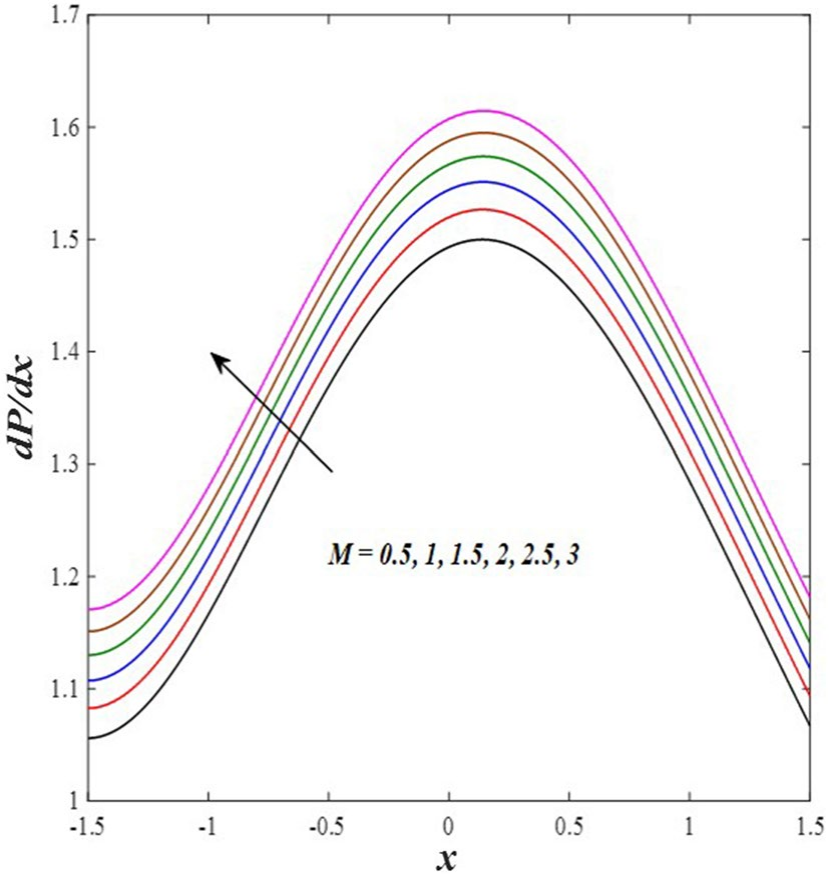
Pressure gradient distribution for different values of *M*.

**Figure 24:**
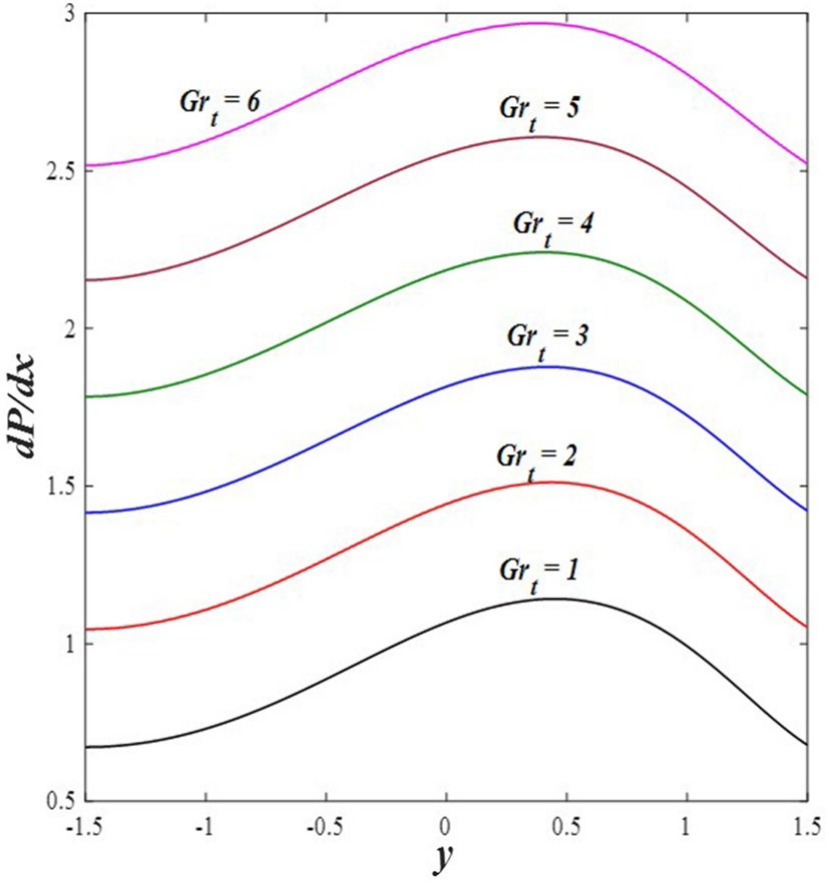
Pressure gradient distribution for different values of *Gr*_t_.

**Figure 25:**
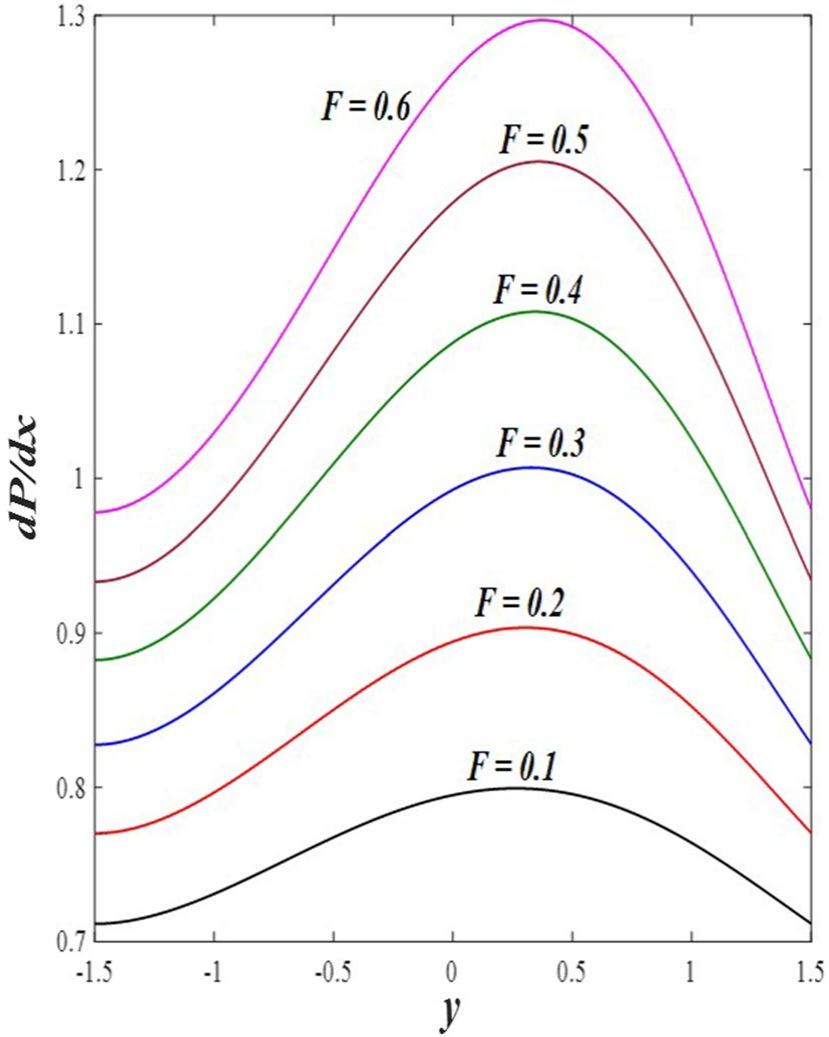
Pressure gradient distribution for different values of *F*.

The pressure gradient plays an important role in the movement of the fluid within the elastic channel, as it is like a machine that works to pump the fluid and its flow at different speeds. Figure 21 explains the effect of rotation $0.5\le \Omega \le 3$ on the distribution of pressure gradients. It has been observed that the pressure gradient increases significantly under the influence of the enhancement in rotation, and this works to support the movement of the fluid inside the channel due to rotation, which in turn resists the large viscosity that characterizes the fluid, it also works to increase the waves occurring in the channel walls, which move with the movement of the fluid inside the channel. Figure 22 shows the negative effect of the Darcy number $0.5\le Da\le 3$ on the distribution of pressure gradients so that the enhancement in the values of the Darcy number reduces the rate of pressure gradient, and the reason behind this is the strengthening of the resistive force resulting from the porous medium. On the other hand, it works to reduce the wave’s incident in the walls of the flexible channel. The support that occurred in the distribution of pressure gradients under the effects of the magnetic field parameter $0.5\le M\le 3$ and the heat Grashof number $1\le Gr\le 6$ can be seen in Figs. 23 and 24. The large response to the pressure gradient contributed to the increase in the rate of fluid pumping into the channel. Finally, Fig. 25 presents the features of the effect of the mean flow coefficient on the distribution of pressure gradients. When looking at the figure, it was noticed that the large response range and the positive direct relationship between this coefficient and this distribution, and this is clear from the remarkable spacing between the curves, if this indicates something, it indicates that both of them help to support the fluid flow inside the flexible channel and help in the regularity of the fluid movement, especially in overcoming the narrow areas of the channel. It is indicated that the reader should pay attention to the fact that the rate of ripples occurring in the walls of the flexible channel during the movement of the fluid inside it is directly proportional to the distribution of pressure gradients.

### Magnetic force, and induced magnetic field distributions

**Figure 26:**
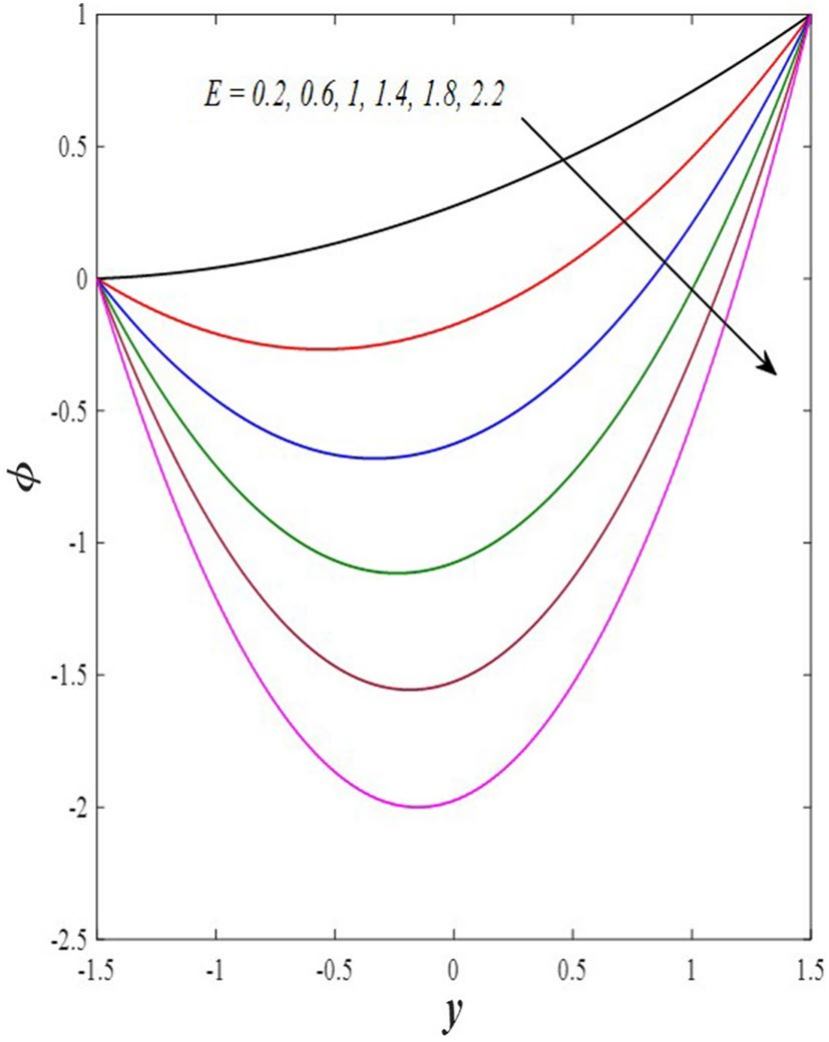
Magnetic force distribution for different values of *E*.

**Figure 27:**
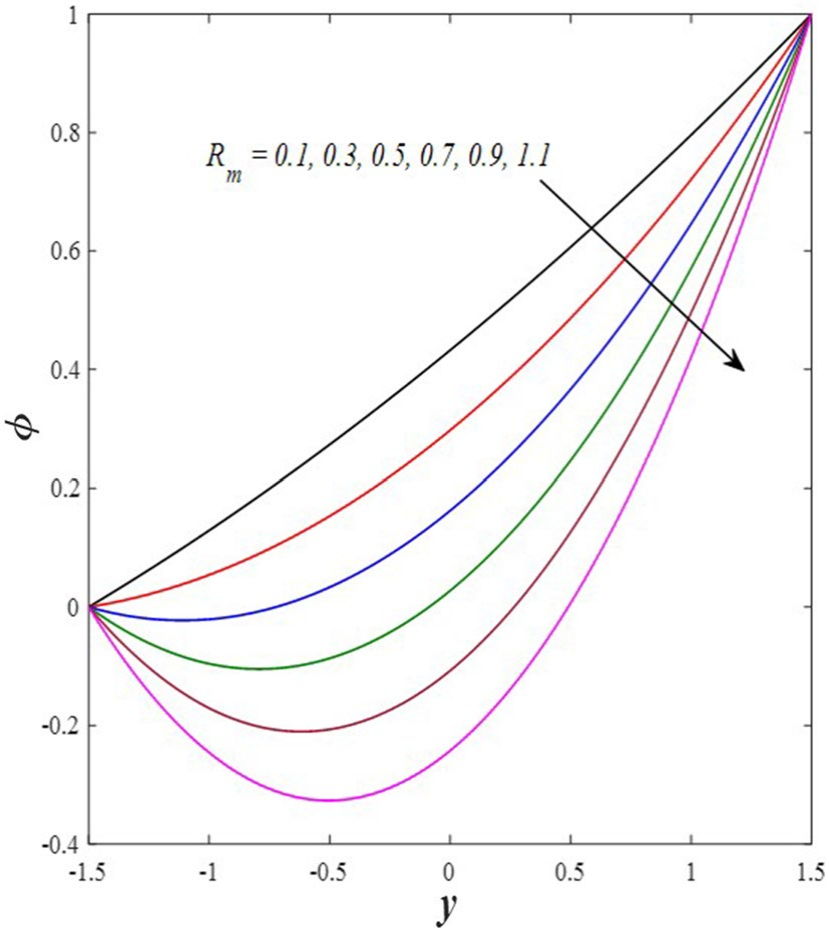
Magnetic force distribution for different values of *R*_*m*_.

**Figure 28:**
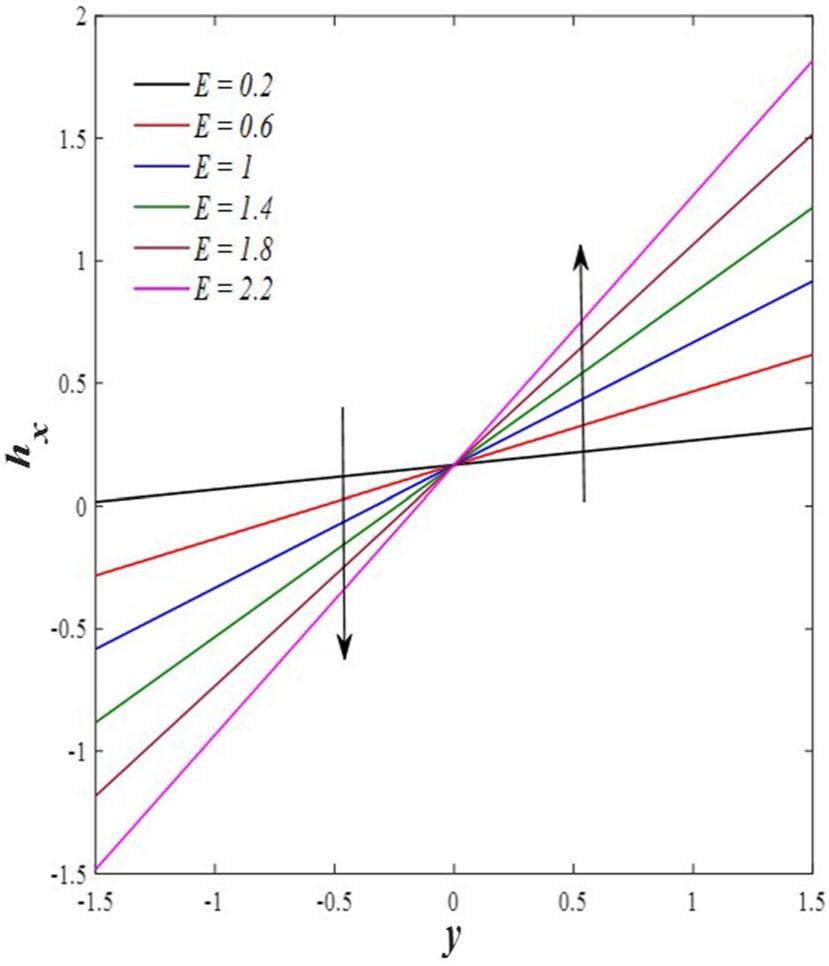
Induced magnetic field distribution for different values of *E*.

**Figure 29:**
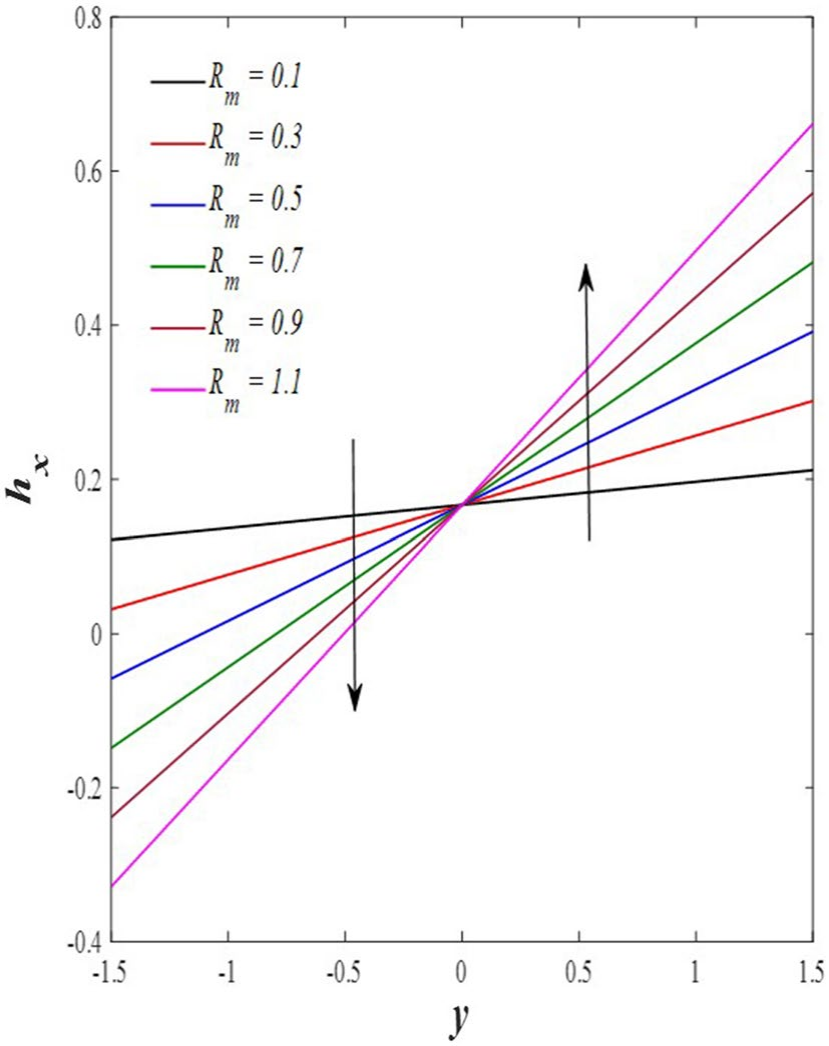
Induced magnetic field distribution for different values of *R*_*m*_.

**Figure 30:**
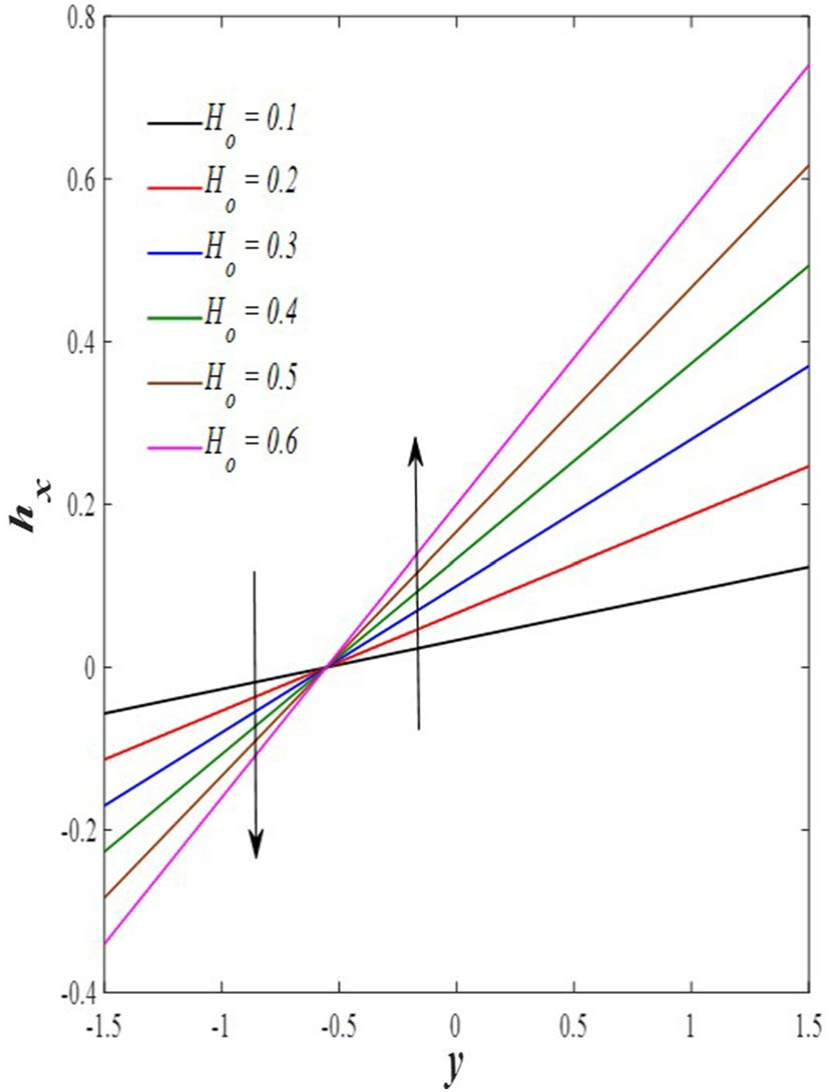
Induced magnetic field for different values of *H*_o_.

The magnetic force is one of the four known forces in nature and is considered the product of the electromagnetic force, or in other words, it is the result of the electromagnetic force, and the main cause of the magnetic force is the movement of charges. For example, when two bodies contain the same charge and have the same direction of motion, a magnetic attraction force is generated between them, while if the direction of motion is opposite, then a magnetic repulsion force is generated. Accordingly, Figs. 26 and 27 studies the impact of electric field strength coefficient $0.2\le E\le 2.2$, and the electric Reynolds number ${0.1\le R}_{m}\le 1.1$ on the distribution of the magnetic force $\phi$. The distribution of the magnetic force that was directed on the movement of the fluid inside the channel under the influence of the two previous parameters becomes at its lowest value if you look at the two figures in order. This indicates that the fluid movement within the channel is subjected to slight values of magnetic force. In contrast, the induced magnetic field is defined in electrical physics as the magnetic field produced by the passage of an electric current through a wire. It is directly responsible for the magnetic force produced when a magnet is brought close to a piece of metal, and its direction is perpendicular to the electric current’s. In the present study, when exposing the fluid that flows inside the channel to an induced magnetic field, it may exhibit a behavior that is recognized by influencing that field with three physical factors, which are, in order, electric field strength coefficient $0.2\le E\le 2.2$, electric Reynolds number ${0.1\le R}_{m}\le 1.1$ and finally the strength magnetic field ${0.1\le \mathcal{H}}_{o}\le 0.6$, this behavior appears in Figs. 28, 29, and 30, respectively, it becomes clear to you dear reader, from the first glance, that the distribution of the induced magnetic field ${h}_{x}$ becomes a decreasing function in the left side of the channel under the influence of the three parameters, respectively, in the interval $-1.5\le y\le 0$ for $E$ and ${R}_{m}$ parameters in Figs. 28 and 29, and interval $-1.5\le y\le -0.5$ concerning parameter ${\mathcal{H}}_{o}$ in Fig. 30, and soon a rapid change occurs in the behavior of the induced magnetic field, it becomes an increasing function in the right side of the channel in distance $0\le y\le 1.5$ for the first and second parameters ($E$ and ${R}_{m}$) and in distance $-0.5\le y\le 1.0$ for the third parameter ${(\mathcal{H}}_{o})$.

### Streamlines profiles and trapping phenomenon

**Figure 31:**
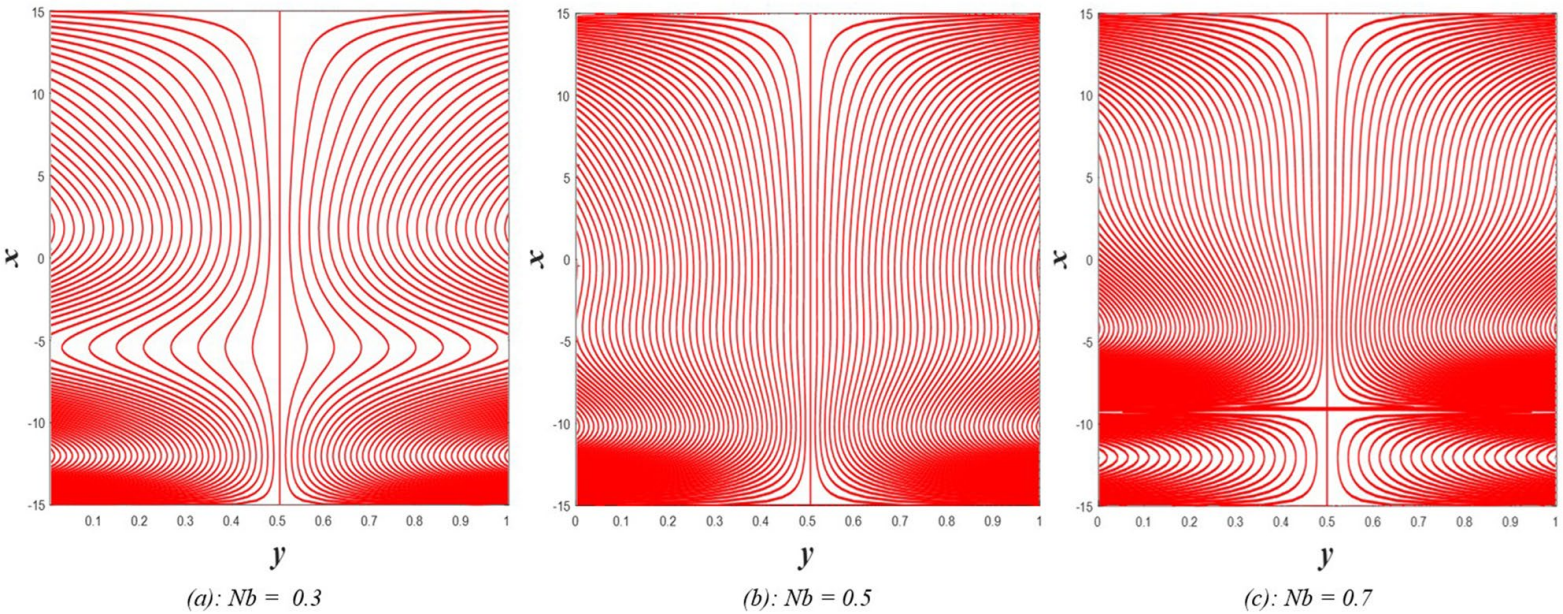
Streamlines profiles for different values of *Nb*.

**Figure 32:**
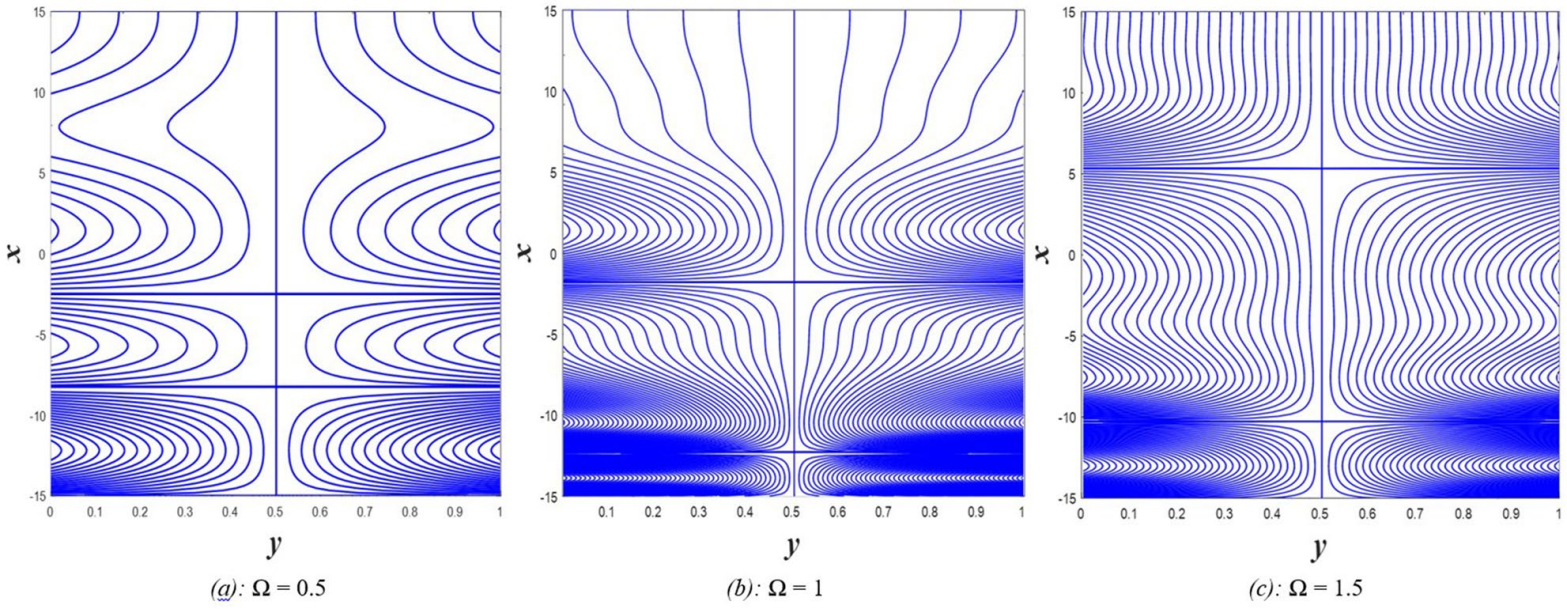
Streamlines profiles for different values of Ω.

**Figure 33:**
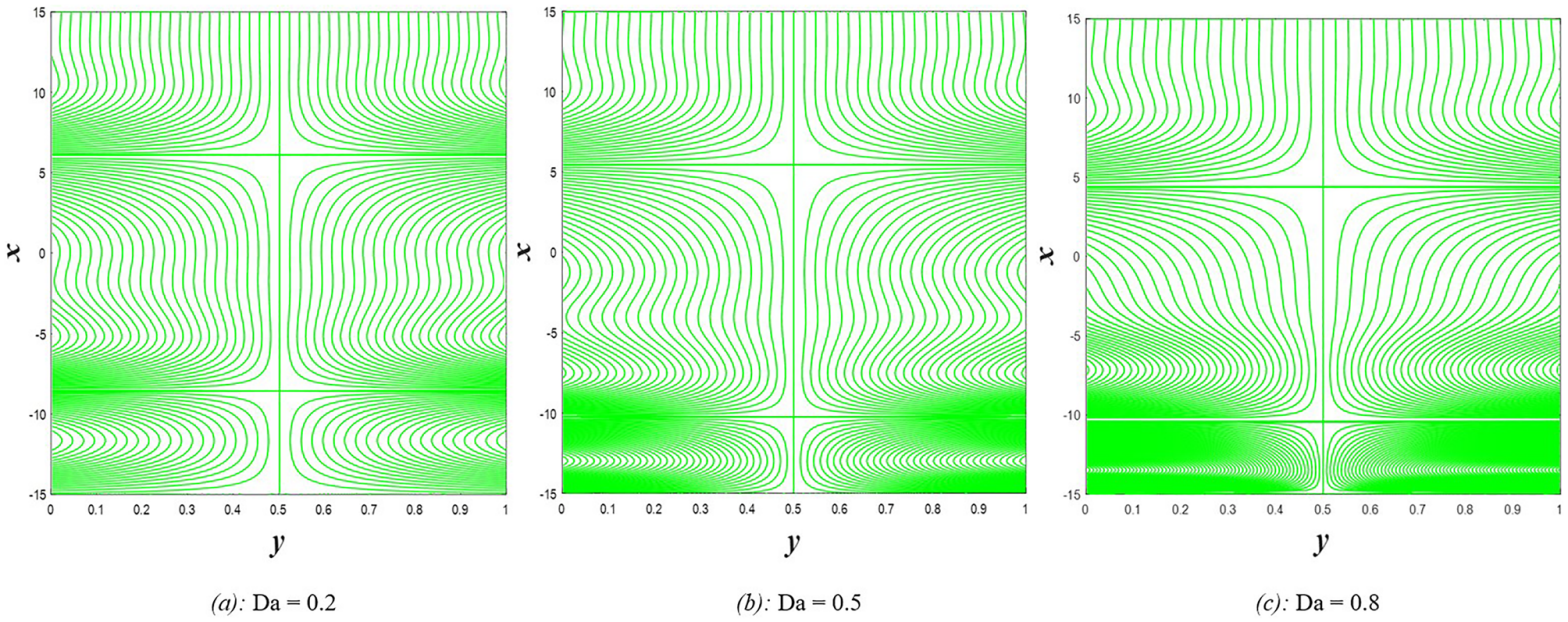
Streamlines profiles for different values of *Da*.

**Figure 34:**
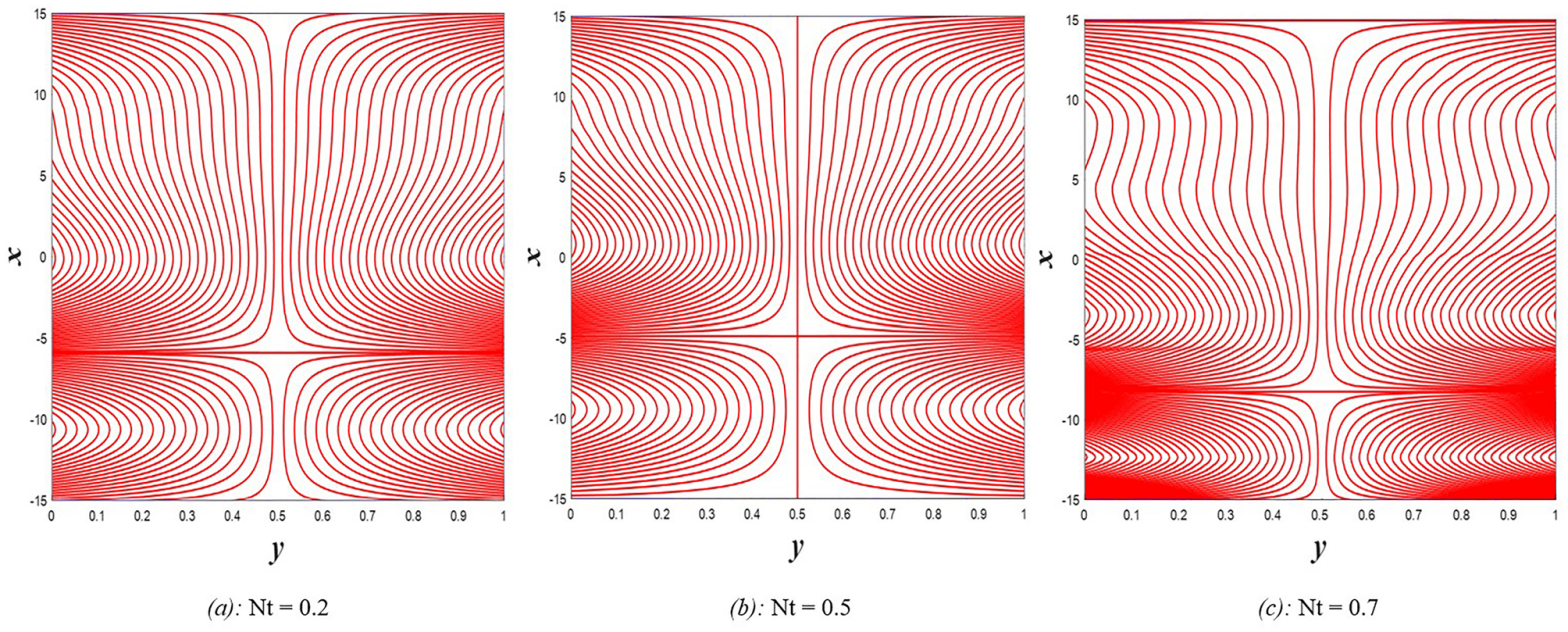
Streamlines profiles for different values of *Nt*.

**Figure 35:**
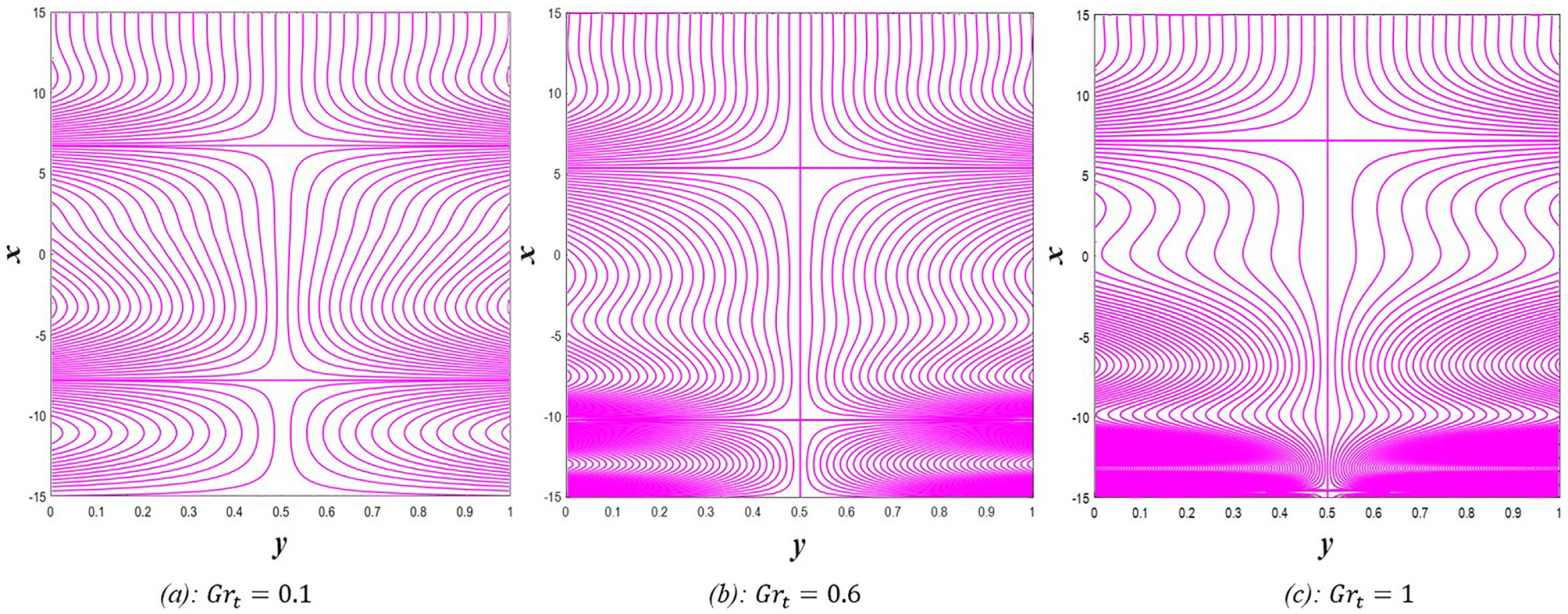
Streamlines profiles for different values of *Gr*_t_.

Trapping is a process that can be seen in the transfer of liquids and can be visualized by drawing streamlines, which is a characteristic of peristaltic motion. Streamlines profiles also play an important role in the study of trapping. A bolus is encapsulated by dividing a streamline when certain conditions are met, and it is carried along with the wave in the wave frame as the wave moves. This practice is referred to as trapping. As a result, the behavior of the fluid flow lines inside the flexible channel under investigation and its connection to the trapping phenomenon are depicted in some significant graphic figures as follows, Fig. 31a–c shows the behavior of streamlines and their impact on the phenomenon of trapping under the influence of different values taken by the Brownian motion coefficient $Nb\in \left\{0.3, 0.5, 0.7\right\}$. It was seen that the thickness of the streamlines increments fundamentally when the qualities taken are expanded by the boundary so more than one bolus starts to show up on the two sides of the channel, they are encircled by the streamlines restricted by the waves, and move with them at a similar wave speed. In the three figures, you can see how different each bolus is from the others in terms of shape. Figure 32a–c was plotted to explain how the behavior of fluid streamlines and the occurrence of the trapping phenomenon are affected by various rotational values of Ω ∈ {0.5,1,1.5}. It has been shown that the thickness of the streamlines is unpredictable, and it increments persistently with the presence of numerous boluses on the two sides of the channel. They observe the presence of pressures on the boluses at the bottom of the channel as a result of the approach of the flow lines in comparison to the top of the channel. They are caught in the ripples that are occurring in the streamlines and move along with them at the same speed. The regularity in the behavior of the streamlines of the fluid flowing through the channel and its effect on the occurrence of the phenomenon of trapping occurs in Fig. 33a–c under the influence of different values taken by the Darcy number $Da\in \left\{0.2, 0.5, 0.8\right\}$, you may notice the growth in the density of the streamlines when passing through the three figures, with no difference in their shapes, with the appearance of boluses on both sides of the channel responsible for the occurrence of the phenomenon of trapping, so that each bolus is trapped by the waves of the streamlines and moves with them at the same speed. Finally, Figs. 34a–c and 35a–c show the behavior of streamlines profiles, their density, and their relationship to the phenomenon of trapping under the influence of different values taken by each of the thermophoresis $Nt\in \left\{0.2, 0.5, 0.7\right\}$ and the heat Grashof number ${Gr}_{t}\in \left\{0.1, 1, 0.6\right\}$, respectively. The enhancement in the values of the two parameters led to a systematic increase in the density of the streamlines with the emergence of more than one bolus on both sides of the channel noting the occurrence of compressions on the boluses at the bottom of the channel as a result of the convergence of the streamlines compared to the boluses above the channel so that these boluses are trapped by ripples that move with it in the same speed. In general, it can be concluded that the phenomenon of trapping resulting from the state of the flow lines that describe the behavior of the fluid inside the channel from the bottom is different from the top so that the phenomenon of trapping increases significantly from the bottom to the top, causing irregular movement in general within the channel.

**Figure 36:**
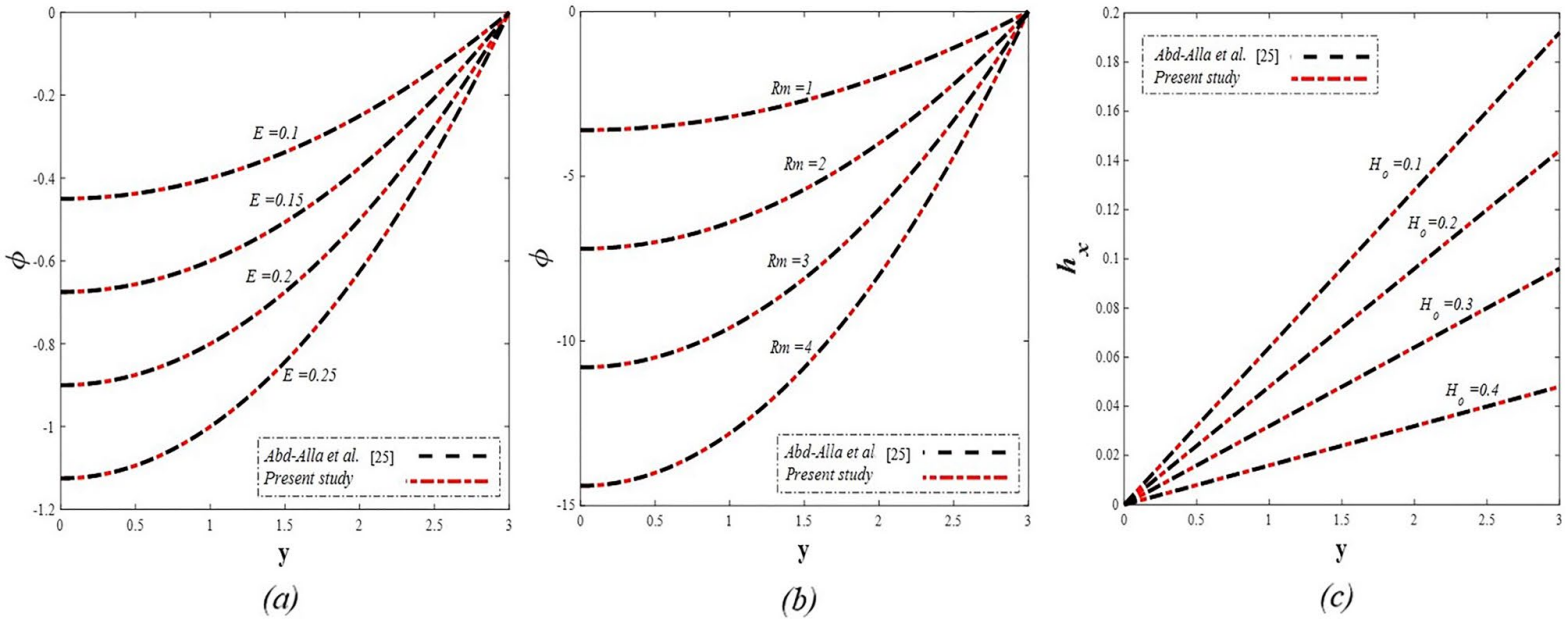
Comparison between Abdalla et al.^24^ and the present study.

The reader ought to focus on; the current work is due to Abdalla et al.^24^ in the absence of a porous medium, nonlinear thermal radiation, heat generation/absorption, viscous dissipation, and Joule heating, and there is no solutal concentration equation and nanoparticle volume fraction equation with the difference in the geometric shape of the channel and the boundary conditions. In contrast, the graphs in Fig. 36a–c show a comparison between the current study and Abd-Alla et al.’s^24^ investigation of the impact of the electric Reynolds number coefficient ${R}_{m}$ and the electric field strength coefficient $E$ on the magnetic force distribution $\phi$, as well as the impact of the strength magnetic field ${\mathcal{H}}_{o}$ on the induced magnetic field distribution ${h}_{x}$. This demonstrates the congruence and similarity between the two investigations, which lends considerable support to the numerical method that was used in obtaining the results of the current study.

## Conclusion

The subject of this study is the double-diffusion peristaltic flow of a non-Newtonian fourth-grade nanofluid through a symmetric vertical elastic channel in the presence of a suitable porous medium. The flow process is also subject to the strength of the induced magnetic field and is affected by rotation, initial stress, nonlinear thermal radiation, heat generation/absorption, viscous dissipation, and Joule heating. The flow process is controlled by a system of nonlinear partial differential equations with boundary conditions that have been transformed into a system of nonlinear ordinary differential equations with a new form of boundary conditions using the long wavelength approximation and ignoring the wave number and the lower Reynolds number. The new system of ordinary differential equations was mathematically solved numerically using the fourth-order Runge–Kutta strategy with the shooting technique in MATLAB code. Finally, graphs were made using MATLAB software to examine the influence of the relative polymorphism of the important physical parameters due to the constant focus on the main distributions namely axial velocity, temperature, solutal concentration, nanoparticle volume fraction, magnetic forces, induced magnetic field, and streamlines profiles. The following is a list of the most important findings of the study:

- Under the influence of the rotation $\Omega$, the initial stress coefficient ${P}^{*}$, the heat Grashof number ${Gr}_{t}$, and the solutal Grashof number ${Gr}_{c}$, the axial velocity distribution $u$ changes from increasing to decreasing.
- Under the influence of the Darcy number $Da$, the nanoparticles Grashof number ${Gr}_{p}$, and the mean flow coefficient $F$, the axial velocity distribution $u$ shifts from a decreasing function to an increasing function.
- The heat Grashof number ${Gr}_{t}$, the solutal Grashof number ${Gr}_{c}$, the nanoparticles Grashof number ${Gr}_{p}$, the Brinkman number $Bn$, the thermophoresis $Nt$, the magnetic field $M$, the heat generation/absorption $\beta$, the Brownian motion $Nb$, the Soret parameter ${N}_{\mathcal{F}T}$, and the Dufour parameter ${N}_{T\mathcal{F}}$ all have a positive impact on the temperature distribution $\theta$.
- The temperature distribution is a decreasing function under effects of the nonlinear thermal radiation $R$ and the temperature ratio ${\theta }_{w}$ work on the decrease of this distribution.
- The distribution of solutal concentration $\gamma$ and nanoparticles volume fraction $\Phi$ were negatively impacted by the heat Grashof number ${Gr}_{t}$, the solutal Grashof number ${Gr}_{c}$, the nanoparticles Grashof number ${Gr}_{p}$, the Brinkman number $Bn$, the thermophoresis $Nt$, the magnetic field $M$, the heat generation/absorption $\beta$, the Brownian motion $Nb$, the Soret parameter ${N}_{\mathcal{F}T}$, and the Dufour parameter ${N}_{T\mathcal{F}}$.
- The solutal concentration $\gamma$ and nanoparticle volume fraction $\Phi$ distributions were positively impacted by the nonlinear thermal radiation $R$ and the temperature ratio coefficient ${\theta }_{w}$.
- The pressure gradients distribution $\frac{\partial P}{\partial x}$ turned into a rising function affected by the rotation $\Omega$, the heat Grashof number ${Gr}_{t}$, the magnetic field $M$, and the mean flow $F$, while it turned into a negative function affected by the Darcy number $Da$.
- The magnetic forces distribution $\phi$ decreases under the influence of the electric field strength $E$ and the electric Reynolds number ${R}_{m}$.
- The behavior of the induced magnetic field distribution ${h}_{x}$ changes between decreasing and increasing under the influence of the electric field strength $E$, the electric Reynolds number ${R}_{m}$, and the magnetic field strength ${H}_{o}$.

## Data Availability

The datasets used and/or analyzed during the current study available from the corresponding author on reasonable request.

## Abbreviations

${\overline{A} }_{i}$: The Rivlin–Ericksen tensors
$a$: Indicates the channel half width
$\overrightarrow{B}$: The magnetic flux density
$Bn$: The Brinkman number
$b$: The wave amplitude
$\widetilde{C}$: The nanoparticles volume fraction
${C}_{o}$: The initial nanoparticles volume fraction
$c$: The wave speed
${c}_{P}$: The specific heat
$\overrightarrow{D}$: The displacement field
${D}_{B}$: The Brownian diffusion coefficient
${D}_{T}$: The thermophoresis diffusion
${D}_{T\mathcal{F}}$: The Dufour diffusivity
${D}_{\mathcal{F}T}$: The Soret diffusivity
$Da$: The Darcy number
$\overrightarrow{E}$: The electric field intensity
$E$: The electric field strength
$Ec$: The Eckert number
$F$: The dimensionless time mean flow rate
${Gr}_{t}$: The heat Grashof number
${Gr}_{p}$: The nanoparticles Grashof number
${Gr}_{c}$: The solutal Grashof number
$g$: The gravitational acceleration
$\overrightarrow{\mathcal{H}}$: The magnetic field
${\mathcal{H}}_{o}$: Constant magnetic field
${\mathcal{H}}^{+}$: The total magnetic field
$Hr$: The Hartman number
${h}_{x}$: The induced magnetic field
$\overline{I }$: The identity tensor
$\overrightarrow{J}$: The current density
$${K}_{p}$$: The permeability.
${k}^{*}$: The mean absorption coefficient
$k$: The thermal conductivity.
${J}_{he}$: The Joule heating
$M$: The magnetic field parameter
$Nb$: The Brownian motion parameter
$Nt$: The thermophoresis parameter
$$P$$: The pressure
${N}_{\mathcal{F}T}$: The Soret parameter
${N}_{T\mathcal{F}}$: The Dufour parameter
$Pr$: The Prandtl number
${P}^{*}$: The Initial stress parameter
${Q}_{o}$: The constant of heat conduction
$\overrightarrow{q}$: The velocity vector
${\overrightarrow{q}}_{r}$: The radiation heat flux
$R$: The thermal radiation parameter
$Re$: The Reynolds number
${R}_{m}$: The magnetic Reynolds number
$\overline{{S }_{i}}$: The extra stress
${\widetilde{S}}_{xx},{\widetilde{S}}_{xy},{\widetilde{S}}_{yy}$: The extra stress tensors
$S$: The Strommer number
$\widetilde{T}$: The fluid temperature
${D}_{s}$: The solutal diffusivity
${T}_{o}$: The Initial temperature
$$tr$$: The trace
$$\widetilde{t}$$: The time
$\widetilde{U},\widetilde{V},\widetilde{u},\widetilde{v}$: The velocity components in the fixed and wave frames
$\widetilde{X,}\widetilde{Y},\widetilde{x},\widetilde{y}$: The fixed frame and the wave frame
$$\lambda$$: The wavelength
${\mu }_{e}$: The magnetic permeability
$\varsigma$: The magnetic diffusivity
$${\varepsilon }^{*}$$: The porosity
${\mu }_{app}$: The apparent viscosity
$\sigma$: The Electrical conductivity
${\rho }_{f}$: The fluid density
${\rho }_{p}$: The density of nanoparticles
$\widetilde{\tau }$: The superscript is the matrix transpose
$$\overrightarrow{\Omega }$$: The rotation
$\nabla$: The differential operator
${\theta }_{w}$: The temperature ratio parameter
$\delta$: The wave number
$${\alpha }_{i}$$: Material constants
$${\beta }_{i}$$: Material constants
$${\gamma }_{i}$$: Material constants
$d/dt$: The material time derivative
$\partial P/\partial x$: The pressure gradient
$\alpha$: The amplitude ratio
$\beta$: The heat generation/absorption parameter
${\left(\rho {c}_{P}\right)}_{f}$: The heat capacity of the fluid
$\mu$: The dynamic viscosity
${\left(\rho {c}_{P}\right)}_{p}$: The effective heat capacity of the nanoparticles
$${\omega }_{3}$$: The vortex
$\gamma$: The non-dimensional solutal concentration function
$\psi$: The stream function
$\overline{\mathbf{T} }$: The Cauchy stress tensor
$\theta$: The non-dimensional temperature function
$\widetilde{\xi }$: The time-mean flow
$\vartheta$: The dimensionless time-mean flow
${\beta }_{\mathcal{F}}$: The volumetric solutal expansion
${\beta }_{T}$: The thermal expansion coefficient
$\zeta$: The wave frame
$\phi$: The non-dimensional magnetic force function
$\Phi$: The non-dimensional concentration of nanoparticles function
${\sigma }^{*}$: The Stefan–Boltzmann constant
$\Delta y$: The step size
$\widetilde{\mathcal{F}}$: The solutal (species) concentration
${\mathcal{F}}_{o}$: The initial solutal (species) concentration
